# Poxvirus A52 protein subverts autophagy flux by blocking autophagosome–lysosome fusion to promote viral replication

**DOI:** 10.1371/journal.ppat.1014137

**Published:** 2026-04-13

**Authors:** Kang Niu, Yongxiang Fang, Yining Deng, Ziyue Wang, Shijie Xie, Junda Zhu, Baifen Song, Wenxue Wu, Zhizhong Jing, Chen Peng

**Affiliations:** 1 National Key Laboratory of Veterinary Public Health and Safety, College of Veterinary Medicine, China Agricultural University, Beijing, China; 2 Lanzhou Veterinary Research Institute, Chinese Academy of Agricultural Sciences, Lanzhou, China; University of Zurich, SWITZERLAND

## Abstract

Many poxviruses are significant zoonotic pathogens threatening public health. Autophagy, a regulated process vital for cellular homeostasis, can participate in defense against virus invasion. However, the relationship between poxviruses and host cell autophagy is not fully understood. This study shows that vaccinia virus (VACV) induces autophagy but blocks autophagosome-lysosome fusion. Modified vaccinia virus Ankara (MVA), an attenuated VACV strain that cannot replicate in most mammalian cells, fails to do so. Both pharmacological inhibition of early autophagy via 3-MA treatment and genetic ablation of ATG3 and ATG7 led to a significant enhancement of MVA replication. The VACV protein A52 inhibits autolysosome formation by disrupting interactions between SNAP29, STX17, and VAMP8, which is crucial for autophagic flux. Importantly, A52 also promotes the degradation of SNAP29, thereby aiding viral replication. Furthermore, SNAP29 is a newly identified host restriction factor for MVA, as its suppression enables MVA replication in human cells. These findings elucidate how poxviruses modulate autophagy for their own replication and further explain MVA’s restriction in human cells.

## Author summary

Many poxviruses are zoonotic pathogens that threaten public health. Autophagy is a delicately regulated process within cells. Nevertheless, the relationship between poxviruses and host cell autophagy remains ambiguous. Here, we show that VACV induces autophagy but blocks the fusion of autophagosome with lysosome, while MVA is unable to accomplish such blockade. Moreover, we identify the viral protein responsible for the inhibition and characterize its mode of action. In addition, VACV A52 also promotes the degradation of SNAP29 via the proteasomal pathway. Importantly, we discover SNAP29 as a new host restriction factor for MVA that is targeted by viral A52. Our findings provide further molecular explanation for the safety of MVA and reveal a novel biological function for poxvirus A52.

## 1. Introduction

Poxviruses, including mpox (MPXV), lumpy skin disease virus (LSDV) and sheeppox virus, continue to pose a significant threat to human and animal health after the successful eradication of smallpox through extensive vaccination efforts [1,2]. Mpox has resulted in infections in over 100,000 individuals across more than 120 countries and over 220 deaths among confirmed cases [3]. LSDV, a member of the *Capripoxvirus* genus, leads to significant economic loss in the cattle industry in affected countries due to decreased milk production and increased mortality rates resulting from its infection [4]. Vaccinia virus (VACV) is the most well-studied prototype poxvirus, and the vaccine strain used to eliminate smallpox. Investigating how poxviruses exploit host cell machinery and employ viral strategies to evade host immune responses is crucial for the development of future vaccines and therapeutic interventions. Modified vaccinia virus Ankara (MVA), an FDA-approved vaccine strain for smallpox and mpox, was generated through repeated passages of its parental strain chorioallantois vaccinia virus Ankara (CVA) in chicken embryo fibroblasts (CEF) [5]. MVA has lost its ability to replicate in most mammalian cells, including almost all human cells [6]. The underlying mechanism is complicated and has been partially elucidated by recent studies [7–10]. Nevertheless, the complex nature of MVA’s restrictions necessitate further investigation.

Autophagy, an evolutionarily conserved, multi-step intracellular biological process [11,12], is involved in a wide array of physiological and pathological processes, including the defense against viral infections [13–18]. Nevertheless, its exact role in controlling viral infections remains ambiguous, as both beneficial and detrimental effects have been observed across different types of viruses. Macroautophagy includes three critical steps: 1) initiation of nucleation; 2) extension of phagocytic vesicle structure to seal the cytoplasmic components and the formation of complete autophagosomes; 3) the fusion of autophagosomes with lysosomes to form autolysosomes, which then leads to degradation of encapsulated contents [19–21]. All these steps are tightly regulated, and reports have shown various strategies employed by viral pathogens to hijack or prohibit all or part of these processes [18]. Emerging evidence has shown that many viruses have evolved mechanisms to evade or inhibit one or multiple steps of the autophagic pathway for their own benefit [22–24]. For example, the interaction between the herpes simplex virus 1 (HSV-1) ICP34.5 and cellular BECN1 facilitates the inhibition of autophagy and increases viral replication [25]; the human cytomegalovirus (HCMV) encodes a homolog of ICP34.5, namely TRS1, which also inhibits autophagy through BECN1 interaction to promote viral replication [26]; the K7 protein of Kaposi’s sarcoma virus (KSHV) promotes Rubicon-BECN1 interaction, which results in the inhibition of VPS34’s enzyme activity and enhances viral survival [27]; the vFLIP protein of KSHV inhibits autophagy by blocking the binding of ATG3 and MAP1LC3B during autophagosome elongation [28].

On the other hand, viruses can exploit autophagic machinery for their replication. The double membrane compartment formed during autophagy can provide a physical platform for viruses to replicate by concentrating on viral intermediate products, and protection of viral components from immune surveillance and degradation. E.g., treatment of cells with an autophagy inducer rapamycin (rapa.) increased the replication of poliovirus, while silencing of autophagic factors reduced viral replication [29]. In addition, electron microscopic analysis of poliovirus-infected cells showed DMVs (double-membrane vesicles) resembling autophagosomes provided a scaffold for viral RNA replication. Moreover, autophagy is also known to be employed by many viruses for non-lytic egress of viral particles [30]. Previous reports showed deletion of several autophagy factors (ATG3, ATG5, BECN1) failed to dampen VACV’s replication in mouse embryonic fibroblast (MEF) cells [31,32]. Using VACV as a model poxvirus, Melanie Krause demonstrated that overexpression of xenophagy receptors SQSTM1, NDP52, and Tax1 Bp1 inhibited poxviral infection. While NDP52 and Tax1 Bp1 were degraded, SQSTM1 initially localized to cytoplasmic virions before relocating to the nucleus. This nuclear translocation depended on SQSTM1’s NLS2 domain and was associated with VACV kinase-mediated phosphorylation of SQSTM1 at T269/S272. These findings indicate that VACV manipulates SQSTM1 early in infection to evade degradation, highlighting poxviruses’ multi-layered regulation of autophagy to promote cytoplasmic replication [33]. VACV fine-tunes mTOR through F17, temporarily exploiting autophagy-related mechanisms while blocking autophagic degradation [34]. This multi-layered regulation allows the virus to exploit beneficial aspects of autophagy while evading its antiviral effects, highlighting the complexity of poxvirus manipulation of host metabolism and autophagy. However, the exact role of autophagy during poxvirus infection in human cells and strategies employed by poxviruses to manipulate autophagy, remain enigmatic.

Through a screening for small-molecule antivirals, we identified several autophagic inhibitors that exhibited modest inhibitory capability on VACV’s replication. Further investigation suggested manipulation of autophagy affected poxvirus’ replication as pharmaceutical inhibition of autophagy at late stage of infection led to enhanced replication of MVA. Interestingly, in comparison to MVA, VACV-WR was able to inhibit the final step of autophagy, namely the formation of autolysosomes. We next identified that VACV protein A52 was the primary factor responsible for the inhibition of autolysosome formation. Our data further characterized the mode of action by which A52 blocked autolysosomes formation and identified its cellular target SNAP29. As insertion of A52 into MVA partially enhanced its replication in human cells, this effect was reminiscent of the improvement observed upon pharmacological inhibition of autophagy at late stages of infection. Moreover, the ectopic expression of SNAP29 inhibited the replication of MVA or a strain of VACV that lacks A52, suggesting the host restriction role of SNAP29 for MVA in human cells. Our findings demonstrate the importance of autophagic modulation in poxvirus replication, report a novel restriction pathway for MVA in human cells, and characterize the molecular mechanism by which VACV blocks the fusion of lysosomes with autophagosomes to promote its replication.

## 2. Results

### 2.1 Differential effects of autophagy inhibitors and autophagy-related genes on poxvirus replication

**Fig 1:**
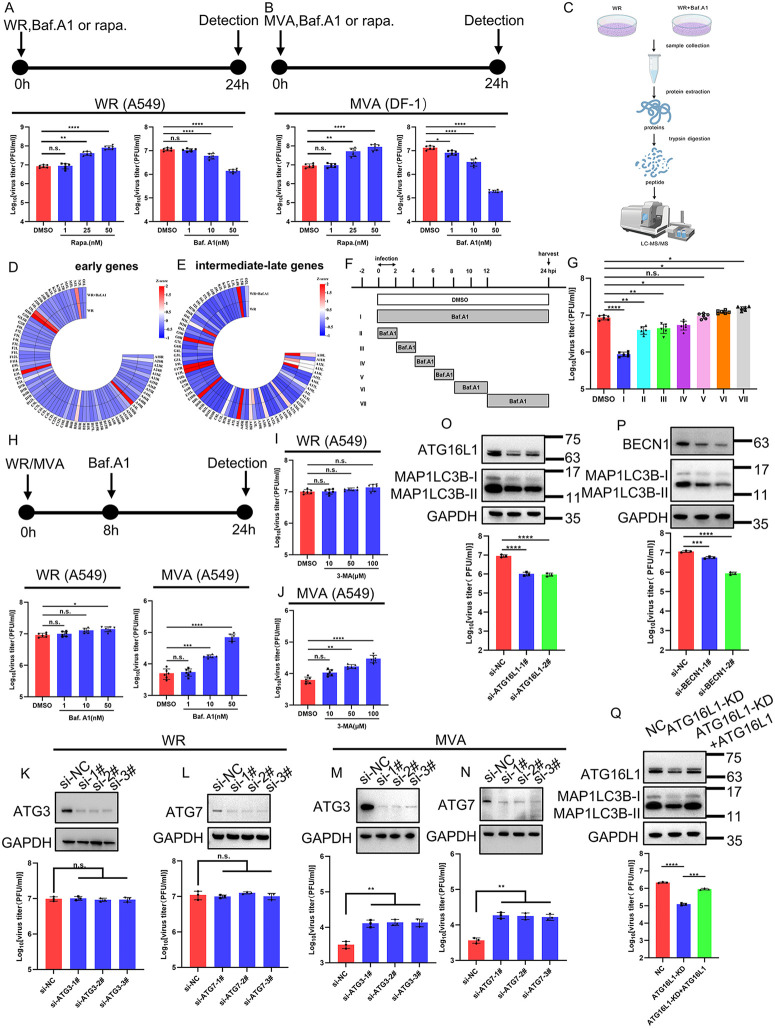
The impact of pharmacological autophagy modulators and key autophagy-related genes on poxvirus replication. Human A549 **(A)** or DF-1 cells **(B)** were infected in triplicate with VACV-WR or MVA at 3 PFU/cell in the presence of rapa. or baf. A1 at the indicated concentrations. Virus yield at 24 hpi was determined on BS-C-1 (VACV-WR) or DF-1 (MVA) cells. **(C)** A549 cells were infected with 3 PFU/cell VACV-WR for 2 h, and then cells were treated with baf. A1 (0.1μM) or not, and samples were collected at 24 hpi. The proteins from the samples were extracted and were enzymatically digested, and then the VACV proteins were quantified by LC-MS/MS, followed by proteomic analysis. Flowchart was created with BioGDP.com. **(D)** Heatmap analysis of early viral proteins of VACV-WR. **(E)** Heatmap analysis of intermediate-late proteins of VACV-WR. **(F)** Strategy of the time-of-drug-addition assay. All samples were infected with the VACV-WR between 0 h and 2 h, and the course of baf. A1 addition was set into seven intervals (I–VII). **(G)** Viral titers of VACV-WR were quantified via plaque assay. **(H)** A549 cells were infected with 3 PFU/cell VACV-WR or MVA for 8 h, followed by the addition of baf. A1 at indicated concentrations. Virus yield at 24 hpi was determined on BS-C-1 (VACV-WR) or DF-1 (MVA) cells. **(I-J)** A549 cells were pretreated with DMSO or 3-MA (10, 50, 100 μM) for 2 h and then were infected with 3 PFU/cell VACV-WR or MVA for 24 h. Viral titers of VACV-WR or MVA were quantified on BS-C-1 or DF-1 cells. **(K-N)** A549 cells were transfected with si-NC as a negative control, siATG3 or siATG7 for 48 h and were subsequently infected with VACV-WR **(K-L)** or MVA **(M-N)** at 3 PFU/cell for 24 h. Viral titers of VACV-WR or MVA were quantified on BS-C-1 or DF-1 cells. **(O-P)** A549 cells were transfected with si-NC as a negative control, siATG16L1 or siBECN1 for 48 h and were subsequently infected with VACV-WR at 3 PFU/cell for24 h, and virus titers were determined by a plaque assay on BS-C-1 cells. **(Q)** WT A549 cells (NC), A549 cells stably expressing shATG16L1 (ATG16L1-KD) and A549 cells stably expressing shATG16L1 cells transfected with KD-resistant ATG16L1 (ATG16L1-KD + ATG16L1) were infected with VACV-WR at 3 PFU/cell for 24 h.Virus titers were determined by a plaque assay on BS-C-1 cells. Data in Fig 1A, 1B, and 1G-Q represent the mean values ± SD of three independent biological experiments (N = 3 biological replicates). P-values were calculated using the one-way ANOVA. Statistics: n.s., not significant, p > 0.05; *P < 0.05; **P < 0.01; ***P < 0.001; ****P < 0.0001.

To determine the impact of pharmacological modulation of autophagy on poxvirus replication, A549 or DF1 cells were infected with VACV-Western Reserve (WR) or MVA at 3 PFU/cell, respectively, in the presence or absence of various dosages of autophagy agonist rapamycin (rapa.), or autophagic degradation inhibitor bafilomycin A1 (baf. A1). Viruses were harvested 24 hours post infection (hpi) for titration. While autophagy agonist rapa. promoted the replication of VACV-WR and MVA in a dose-dependent manner, baf. A1 effectively suppressed replication of both viruses (Fig 1A and 1B). Importantly, across all concentrations tested, no significant reduction in cell viability was observed (S1A–S1D Fig). To explore if pharmaceutical modulation of autophagy affected viral mRNA abundance and DNA replication, A549 cells were infected with VACV-WR in the presence of rapa. or baf. A1, and total RNA and DNA were harvested for mRNA and viral genomic DNA quantification using quantitative Realtime-PCR (qRT-PCR) [35]. Cytosine arabinoside (AraC) was included as a positive control as it was able to ablate viral post-replicative gene expression [36]. To our surprise, mRNA levels of selected viral early (E3L), intermediate (D13L) and late genes (A3L), as well as viral DNA replication were not significantly affected by rapa. or baf. A1 treatment, although AraC abolished both viral DNA replication and post-replicative gene transcription (S1F–S1I Fig). Next, to assess the impact of baf. A1 on global viral protein levels, A549 cells were infected with VACV-WR (3 PFU/cell) for 2 h, followed by treatment with baf. A1 or no treatment. Samples were collected at 24 hpi, and proteins were extracted, enzymatically digested, and analyzed using liquid chromatography–mass spectrometry (LC–MS/MS), followed by proteomic analysis (Fig 1C). Compared with the WR-infected control, the baf. A1 + WR group showed no significant change in early protein levels. While the majority of the post-replicative (intermediate and late) proteins also were unchanged upon baf. A1 treatment, several late proteins—including A3, I7, A17, G7, H3, A14, A10, A12, and G6—were significantly reduced (S1 Table and Fig 1D and 1E). Interestingly, proteins that were decreased by baf. A1 treatment (A3, I7, A17, G7, H3, A14, A10, A12, G6) are mainly involved in virus assembly, maturation, membrane formation, protein processing, and virus morphogenesis. To assess the specific stage of the viral replication cycle targeted by baf. A1, we performed a time-of-addition assay in A549 cells infected with VACV-WR. Cells were infected with VACV-WR at 3 PFU/cell and baf. A1 was added at 50 nM at different time points during the experiment (illustrated in Fig 1F). We observed that the viral titer was strongly inhibited when baf. A1 was present throughout the experiment (I), or slightly inhibited during the 0–2 h (II), 2–4 h (III) and 4–6 h (IV) time periods of viral infection. However, the change in viral titer was not significant when baf. A1 was added after 6 hpi. Interestingly, the viral titer increased when baf. A1 was present during the 8–12 h (VI) and 12–24 h (VII) stages of viral infection (Fig 1G). As the entry of VACV was dependent on the endosomal pathway, which requires decrease of pH. We suspected the observed inhibitory effect was due to the disruption of virus entry as baf. A1 was known to affect pH through disrupting ATPase activity. To further examine the effect of baf. A1 at the post-entry steps of virus replication, A549 cells were infected with WR or MVA virus at 3 PFU/cell for 8 h, and then treated with baf. A1 (to exclude its influence on the viral entry phase). Samples were then collected at 24 hours post-infection for quantification. The results showed that lower concentrations (1 nM, 10 nM) of baf. A1 did not negatively affect the replication of VACV-WR, but mildly promoted its replication at 50 nM (Fig 1H). Surprisingly and interestingly, baf. A1 dose-dependently promoted the replication of MVA in A549, a cell line non-permissive for MVA (Fig 1H). Next, we exploited the effect of other autophagy inhibitor on virus replication including 3-MA, an early-stage autophagy inhibitor. VACV-infected A549 cells were treated with 3-MA at various dosages and virus yield was monitored. The results were similar to that observed with baf.A1 in which 3-MA promoted the replication of MVA in a dose-dependent manner but failed to affect the replication of VACV-WR (Fig 1I and 1J). Importantly, none of the tested concentrations resulted in a significant decrease in cell viability (S1E Fig).

As small-molecule chemicals may present off-target effects, we next verified our observation by suppressing various factors important for autophagy regulation, including ATG3, ATG7, ATG12, BECN1 and ATG16L1 [37]. Human A549 cells were transfected with siRNA targeting the above-mentioned genes as well as si-NC as a negative control for 48 h prior to VACV-WR infection and viral progenies were collected at 24 hpi and quantified by the method described above. As shown in Figs 1K, 1L and S1J, transfection of siATG3, siATG12 and siATG7 effectively reduced the levels of corresponding proteins, but failed to influence viral titers. Interestingly, knockdown of ATG3 and ATG7 promoted the replication of MVA in A549 cells (Fig 1M and 1N). In addition, suppression of ATG16L1 and BECN1 significantly inhibited viral replication (Fig 1O and 1P). Notably, double knockdown of ATG7 with ATG16L1 or with BECN1 produced phenotypic effects on viral titers similar to those observed with single knockdown of ATG16L1 or BECN1 (S1K–S1N Fig), suggesting that ATG16L1 and BECN1 influence viral replication independently of ATG7. Next, we generated a cell line stably expressing shATG16L1, as well as its revertant control expressing a KD-resistant ATG16L1 and further examined viral replication within these cells. The titers of VACV-WR were reduced by approximately 10-fold change (Fig 1Q) when ATG16L1 was stably knocked down compared to that in the control cells, but the reduction was compensated in the revertant control, in which the expression of ATG16L1 was rescued (Fig 1Q). In summary, in permissive cell lines (A549 and DF1), rapamycin promoted the replication of both WR and MVA, whereas baf. A1, when present throughout the infection, inhibited their replication. The effect of baf. A1 on VACV-WR replication was dependent on the timing of addition. Treatment with 3‑MA, as well as knockdown of ATG3 and ATG7, significantly enhanced MVA’s replication in its non‑permissive cell line A549, while exerting only minimal effects on the replication of WR.

### 2.2 Infection of VACV-WR or MVA leads to the accumulation of MAP1LC3B-II and autophagosomes

**Fig 2:**
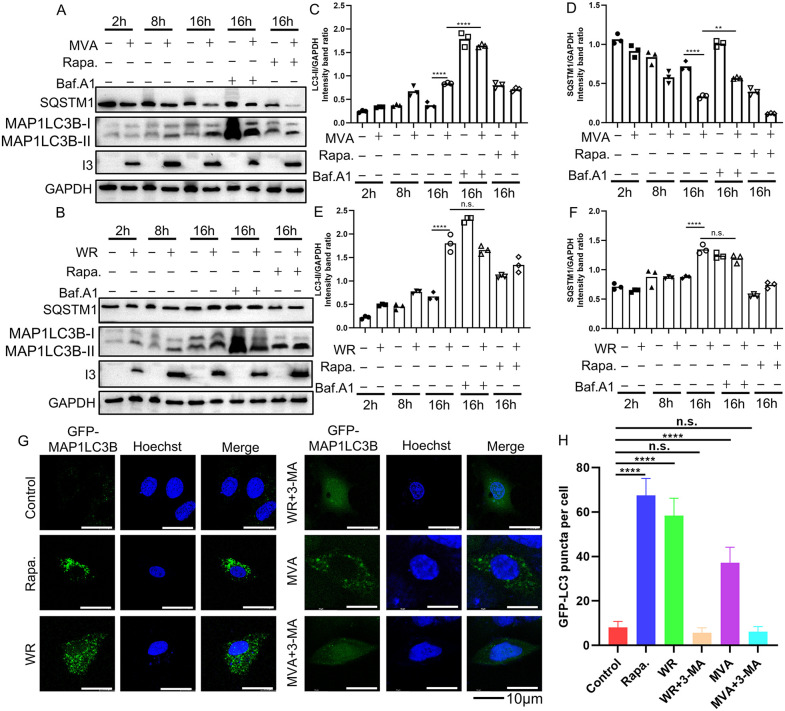
VACV infection promoted activation of autophagy. A549 were infected with MVA **(A)** or VACV-WR **(B)** at 3 PFU/cell in the presence or absence of rapa. (100nM) or baf. A1 (0.1μM), and cell lysates were collected at indicated time points and subjected to Western blotting analysis with anti-SQSTM1, anti-MAP1LC3B, anti-I3, and anti-GAPDH antibodies. The quantification of MAP1LC3B-II and SQSTM1 was shown in Fig 2C-F. **(G)** A549 cells stably expressing GFP-MAP1LC3B on coverslips were infected with VACV-WR, MVA at 3 PFU/cell or treated with 3-MA (1mM) or rapa. (100nM) and cells were fixed at 12 hpi, stained with Hoechst and observed with a fluorescent confocal microscope. **(H)** Numbers of GFP-MAP1LC3B puncta (representing autophagosomes) were quantitated with Image J. Three independent biological replicates were performed, with 15 cells quantified per replicate. Scale bars represent 10 μm. Data in Fig 2A, 2B, and 2G are representative of three independent experiments (N = 3 biological replicates). Statistics: n.s., not significant, p > 0.05; **P < 0.01; ****P < 0.0001 by one-way ANOVA.

We next aimed to investigate if infection with VACV-WR resulted in changes in cellular autophagic regulation. As the conversion of microtubule-associated protein 1A/1B-light chain 3-I (MAP1LC3B-I) to MAP1LC3B-II and the autophagic degradation of SQSTM1, a main cargo degraded by autolysosomes, are considered classic molecular markers for autophagic flux, we first monitored the status of these two markers in A549 cells infected with VACV-WR or MVA at 3 PFU/cell. In both MVA and VACV-WR-infected cells, the conversion from MAP1LC3B-I to MAP1LC3B-II was observed upon infection since 8 hpi (Fig 2A and 2B). Interestingly, although MVA infection led to a discernible decrease of SQSTM1 (Fig 2A), the level of SQSTM1 in VACV-WR-infected cells was comparable to that of the mock cells, and remained unchanged throughout the infection (Fig 2B). Treatment of MVA-infected cells by Baf. A1 enhanced the conversion from MAP1LC3B-I to MAP1LC3B-II and led to an accumulation of SQSTM1. Whereas rapa. treatment did not significantly affect MAP1LC3B lipidation, it led to further degradation of SQSTM1 in MVA-infected cells. The effect of baf. A1 and rapa. was not obvious in VACV-WR-infected cells. The quantification of MAP1LC3B-II and SQSTM1 was shown in Fig 2C-2F. These data suggest that VACV-WR blocks the autophagic flux but not MVA.

We next verified the observation by using an alternative assay, in which a GFP-labeled MAP1LC3B was stably expressed in A549 cells and the formation of green puncta in cells indicated accumulation of autophagosomes [38]. Although MAP1LC3B puncta were observed at a very low level in untreated control cells, the treatment of rapa., infection with VACV-WR or MVA led to observable accumulation of MAP1LC3B puncta (Fig 2G). In addition, treatment with 3-MA significantly inhibited accumulation of MAP1LC3B puncta in VACV-WR and MVA infected cells. Numbers of GFP-MAP1LC3B puncta (representing autophagosomes) were quantitated with Image J in Fig 2H.

Taken together, these data demonstrated that VACV infection resulted in the conversion of MAP1LC3B-I to MAP1LC3B-II, degradation of SQSTM1 and the accumulation of autophagosomes. Importantly and surprisingly, whereas VACV-WR was able to inhibit the degradation of SQSTM1, MVA was unable to accomplish such inhibition.

### 2.3 VACV-WR, but not MVA, is able to block the fusion of autophagosome with lysosome

**Fig 3:**
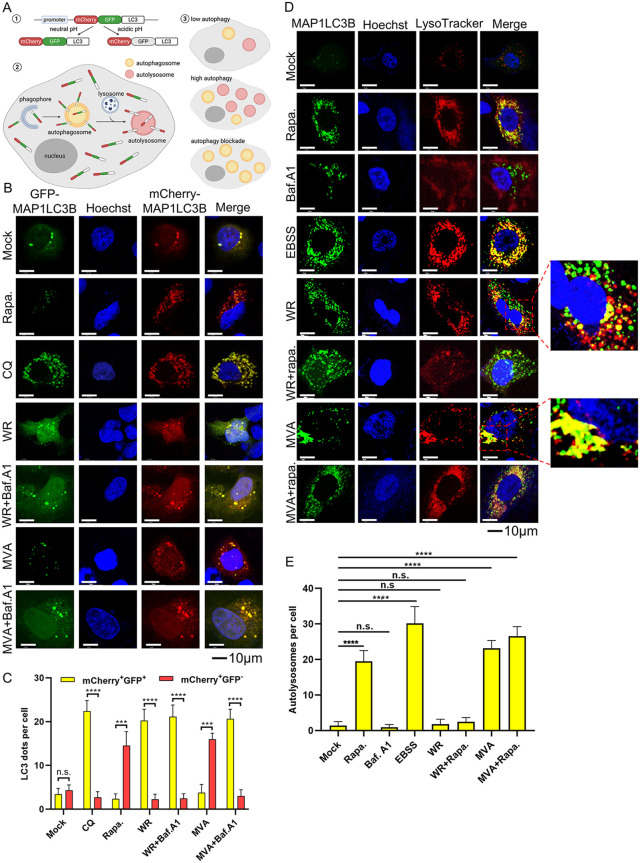
VACV-WR and MVA differed in their ability to inhibit autolysosome formation. **(A)** Diagram illustrating the working principle of the reporter plasmid mCherry-GFP-MAP1LC3B. **(B)** A549 cells grown on coverslips were transfected with the mCherry-GFP-MAP1LC3B plasmid for 24 h and then were mock-infected, infected with VACV-WR or MVA at 3 PFU/cell at the presence or absence of baf. A1(50nM), or treated with CQ (40μM) or rapa. (100nM) for 12 h. Cells were fixed, stained with Hoechst and analyzed with a fluorescent confocal microscope. Scale bars indicate 10 μm. **(C)** The graph shows the quantification of autophagosomes. Three independent biological replicates were performed, with 15 cells quantified per replicate. And bars represent mean values ± SD of three independent biological replicates. **(D)** A549 cells stably expressing MAP1LC3B were infected with VACV-WR, MVA at 3 PFU/cell or treated with baf. A1 or rapa. as described above. Lysotracker was added to live cells to stain lysosomes. **(E)** Images were taken as described above and quantification of colocalization was analyzed using Image J software. Three independent biological replicates were performed, with 15 cells quantified per replicate. Data in Fig 3C and 3E represent the mean values ± SD of three independent biological replicates (N = 3 biological replicates). Statistics: n.s., not significant, p > 0.05; ****P < 0.0001 by two-sided Student’s t test or one-way ANOVA.

To further elucidate the difference between VACV-WR and MVA in autophagy regulation at a finer resolution, we employed an assay to differentiate complete and incomplete autophagy during virus infection [38]. In this assay, a reporter plasmid containing a tandemly connected mCherry-GFP-MAP1LC3B was used to transfect A549 cells 24 h prior to VACV-WR or MVA infection. GFP is sensitive to and can be dampened in an environment with acidic pH, such as that in the lysosomes, whereas the mCherry is not [38]. When autophagosomes are not fused with lysosomes, which suggests incomplete autophagy, both green and red puncta can be observed and appear as yellow puncta in merged images. In contrast, when autophagosomes fuse with lysosomes to complete autophagy, green puncta would rapidly disappear and only red puncta are observed (illustrated in Fig 3A) [38]. A549 cells transfected with the reporter plasmid were infected with VACV-WR or MVA at 3 PFU/cell and cells were fixed at 12 hpi and subjected to confocal microscopic analysis. In mock-infected cells, both green and red puncta were observed at a very low level (Fig 3B). In rapa. treated cells, the level of green signal remained low while red signal overwhelmed, suggesting that the fusion of lysosomes with autophagosomes occurred. However, when chloroquine (CQ) was added, the fusion of autophagosomes with lysosomes was inhibited as both green and red signals were detected and appeared as yellow in merged images (Fig 3B), indicating incomplete autophagy. Importantly, in VACV-WR-infected cells, both green and red signals were captured (Fig 3B), indicating incomplete autophagy. Interestingly and remarkably, in MVA-infected cells, the pattern of red/green signal was similar to that of rapa. -treated cells, demonstrating successful fusion of autophagosomes with lysosomes, which dampened the GFP signals (Fig 3B). Importantly, in MVA + baf.A1 and WR + baf.A1 groups, both green and red signals were captured (Fig 3B), indicating that baf. A1 was able to inhibit the fusion of autophagosomes with lysosomes (impaired lysosomal degradation). We performed quantitation of the data by counting green and red puncta using Image J and the results were shown in Fig 3C. Moreover, we repeated this experiment in HeLa cells and observed similar results (S3A and S3B Fig).

We next investigated the co-localization of lysosomes and autophagosomes, which is a prerequisite for the fusion of autophagosomes with lysosomes. A549 cells that stably express MAP1LC3B were treated with the indicated drugs and subsequently infected with VACV-WR or MVA for 12 h prior to fixation and staining. LAMP1 was used to stain lysosomes. In agreement with our previous observation, rapa. and EBSS, which are autophagy agonists, promoted colocalization of autophagosomes and lysosomes (S3C Fig). The same phenomenon was observed in MVA-infected cells (S3C Fig). However, in VACV-WR-infected cells, lysosomes were not found to be colocalized with MAP1LC3B (S3C Fig), indicating the blockage of the fusion of the two. Quantitation of autolysosomes were shown in S3D Fig. These observations were verified in live cells in which lysotracker (Fig 3D and 3E), a permeable lysosome marker, was used to label the presence of lysosomes.

Taken together, our results demonstrated that VACV-WR showed the capability to block the fusion of autophagosomes with lysosomes while MVA was unable to exert such blockage. These findings led us to hypothesize that VACV-WR may encode a gene(s), whose product(s) may be responsible for blocking autophagosome-lysosome fusion, which was lost in MVA during its repeated passages in CEF cells.

### 2.4 VACV A52 inhibits autophagosome-lysosome fusion

**Fig 4:**
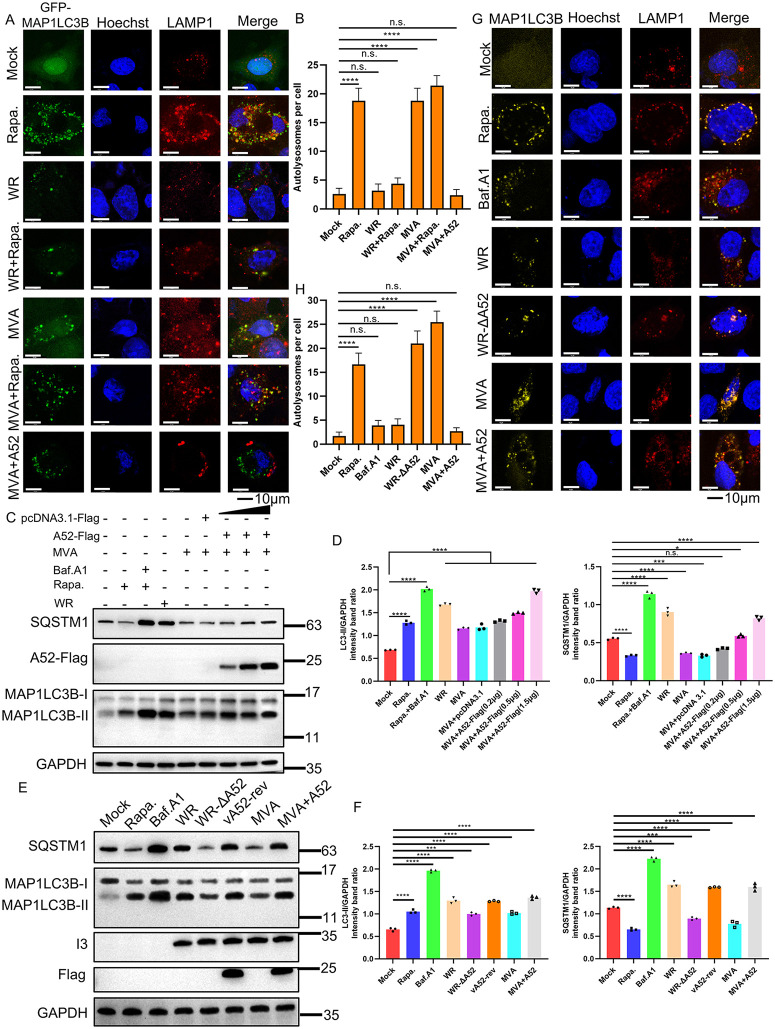
The effect of A52 on the fusion of autophagosome with lysosome. **(A)** A549 cells stably expressing GFP-MAP1LC3B were left untreated, infected with WR or MVA at 3 PFU/cell in the presence or absence of rapa. (100nM) or transiently transfected with a plasmid encoding a Flag-tagged A52 (1500 ng) prior to MVA infection. At 12 hpi, cells were then fixed, permeabilized, blocked, and stained with primary antibodies to LAMP1 and followed by fluorescent conjugated secondary antibodies. Hoechst was used to stain nucleus. Scale bars represent 10 μm. Numbers of autolysosomes were quantitated in Fig 4B. Three independent biological replicates were performed, with 15 cells quantified per replicate. **(C)** Human A549 cells were transfected with a vector encoding A52-Flag at concentrations of 0.1, 0.5, and 1.5 μg/mL for 24 h and then infected with MVA at 3 PFU/cell for 12 h. WR-infected cells and cells treated with baf. A1 and rapa. were included as controls. Cell lysates were analyzed using SDS-PAGE followed by Western blotting analysis with anti-SQSTM1, anti-Flag, anti-MAP1LC3B, or anti-GAPDH antibodies. The quantification of MAP1LC3B-II and SQSTM1 was shown in Fig 4D. **(E)** Human A549 cells were infected with WR, WR-ΔA52, vA52-rev, MVA or MVA + A52 at 3 PFU/cell or treated with baf. A1 (0.1μM) or rapa. (100nM) for 12 h. Cell lysates were analyzed using SDS-PAGE and Western blotting with anti-SQSTM1, anti-I3, anti-MAP1LC3B, or anti-GAPDH antibodies. The quantification of MAP1LC3B-II and SQSTM1 was shown in Fig 4F. **(G)** A549 cells were infected with WR, WR-ΔA52, MVA, MVA + A52 at 3 PFU/cell or treated with baf. A1 (0.1μM) or rapa. (100nM) for 12 h. Cells were then fixed, permeabilized, blocked, and stained with primary antibodies to LAMP1 or MAP1LC3B and followed by fluorescent conjugated secondary antibodies. Hoechst was used to stain nucleus. Scale bars represent 10 μm. Numbers of autolysosomes were quantitated in Fig 4H. Three independent biological replicates were performed, with 15 cells quantified per replicate. Data in Fig 4D and 4F are shown as dots, and the bar represents the mean value. Data in Fig 4B and 4H represent the mean values ± SD of three independent experiments (N = 3 biological replicates). Data in Fig 4A-H are representative of three independent experiments. Statistics: n.s., not significant, p > 0.05; *P < 0.05; **P < 0.01; ***P < 0.001; ****P < 0.0001 by one-way ANOVA.

To identify the gene(s) responsible for the inhibition of the fusion, we compared the genomes of VACV-WR and MVA for the identification of candidate genes. As point mutations were found throughout the genome in MVA in comparison to VACV-WR, we decided to prioritize the genes that are either completely absent or those that exhibit more than 50% deletions. A total of 20 genes were found to be either completely absent or largely truncated (more than 50% deleted) in MVA in comparison to VACV-WR. These genes were codon-optimized, synthesized and then cloned into a mammalian cell expression vector pcDNA3.1. As not all genes cloned were expressed successfully, only those that expressed effectively were further investigated. To identify the autophagosome-lysosome fusion inhibitor, A549 cells that stably express GFP-MAP1LC3B were transfected with the above genes and then infected with MVA at 3 PFU/cell. The infected cells were then fixed and observed directly for GFP or stained with an anti-LAMP1 antibody. A positive target would be a viral protein that disrupts the colocalization of GFP-MAP1LC3B and LAMP1, as observed in VACV-WR-infected cells (Figs 4A and S3A). In addition, all candidate genes were screened for their ability to block SQSTM1 degradation upon MVA infection (S3B Fig and S3C). Using these assays, we screened 20 VACV proteins and identified WR178 (designated A52 in VACV-Copenhagen strain) as a positive candidate. The results showed A52 was able to disturb the colocalization of MAP1LC3B and LAMP1 observed in MVA-infected cells (Fig 4A). Numbers of autolysosomes were quantitated in Fig 4B. Moreover, although MVA infection led to degradation of SQSTM1, transfection of A52 was able to avert this phenomenon in a dose-dependent manner (Fig 4C). The quantification of MAP1LC3B-II and SQSTM1 was shown in Fig 4D.

To further investigate the biological function of A52, we replaced the open reading frame (ORF) of A52 in VACV-WR with a cassette containing a GFP to generate a recombinant virus that lacks A52, designated VACV-WR-ΔA52. A revertant control in which A52 was inserted back into its original locus was also generated (vA52-rev). In addition, a p11 (synthetic poxvirus promoter)-driven-A52 ORF was inserted into the intergenic region between ORF069 and ORF070 in MVA to generate a recombinant MVA expressing A52, which was designated MVA + A52. Both recombinant viruses were plaque-purified and successful deletion or insertion was confirmed with Sanger sequencing. Experiments described in Fig 4E and 4G were performed to monitor the autophagic degradation of SQSTM1 and co-localization between MAP1LC3B and lysosomes upon infection with these recombinant viruses. Compared to WR, the level of SQSTM1 was much lower in cells infected with WR-ΔA52, indicating WR-ΔA52’s ability to block SQSTM1 degradation was compromised by depleting A52. Importantly, infection with the revertant strain vA52-rev significantly increased the level of SQSTM1. Moreover, insertion of A52 into MVA was able to increase the level of SQSTM1 upon MVA’s infection (Fig 4E). The quantification of MAP1LC3B-II and SQSTM1 was shown in Fig 4F.

Next, we observed the subcellular localization of endogenous MAP1LC3B and lysosomes in A549 cells infected with WR, WR-ΔA52, MVA or MVA + A52. The results indicated that A52 was essential for disrupting the colocalization between endogenous MAP1LC3B and lysosomes as both VACV-WR-ΔA52 and MVA were unable to complete such disruption (Fig 4G). Numbers of autolysosomes were quantitated in Fig 4H. In summary, our results demonstrated that VACV A52 was likely associated with the inhibition of the fusion of autophagosomes with lysosomes.

### 2.5 A52 interacts with human SNAP29

**Fig 5:**
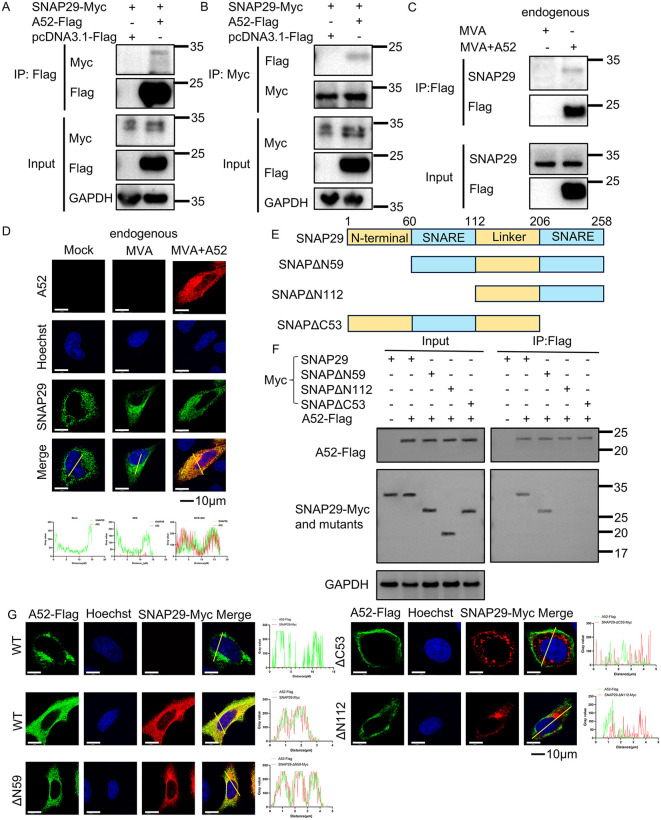
A52 is associated with human SNAP29. Human A549 cells were co-transfected with plasmids encoding Myc-tagged SNAP29 and Flag-tagged A52 for 36 h. Cell lysates were pre-cleared with control magnetic beads and then incubated with Flag-conjugated **(A)** or Myc-conjugated **(B)** beads at 4˚C for 18 h. Beads were extensively washed, and proteins were eluted with SDS loading buffer and analyzed using SDS-PAGE and Western blotting described above. **(C)** A549 cells were mock-infected or infected with MVA + A52 at 3 PFU/cell for 24 h. Cell lysates were pre-cleared with control magnetic beads or Flag-conjugated beads at 4˚C for 18 h. Beads were extensively washed, and proteins were eluted with SDS loading buffer and analyzed using SDS-PAGE and Western blotting with anti-SNAP29, anti-Flag antibodies. **(D)** A549 cells grown on coverslips were infected with MVA or MVA + A52 at 3 PFU/cell for 12h. Cells were then fixed, permeabilized, blocked, and stained with primary antibodies to SNAP29 or Flag and followed by fluorescent conjugated secondary antibodies. Hoechst was used to stain nucleus. The panels below show the fluorescence intensity profile of SNAP29 (green) and A52 (red) measured along the line drawn by Image J. Scale bars represent 10 μm. **(E)** A series of SNAP29 truncation mutants were constructed and illustrated. **(F)** A549 cells were co-transfected with vectors encoding Myc-tagged SNAP29 or its mutants, and a Flag-tagged A52 for 36 h. Cell lysates were pre-cleared with control magnetic beads or Flag-conjugated beads at 4˚C for 18 h followed by extensive washing. Proteins were eluted with SDS loading buffer and resolved by SDS-PAGE followed by Western blotting analysis using primary antibodies for Flag, Myc and GAPDH. Data in Fig 5A, 5B, 5C, 5D, and 5F are representative of three independent experiments. **(G)** A549 cells grown on coverslips were transfected with vectors encoding Myc-tagged SNAP29 or its mutants, and a Flag-tagged A52 for 24 h. Cells were then fixed, permeabilized, blocked, and stained with primary antibodies to Myc or Flag and followed by fluorescent conjugated secondary antibodies. Hoechst was used to stain nucleus. The right panels show the fluorescence intensity profile of A52-Flag (green) and Myc-tagged SNAP29 or its mutants (red) measured along the line drawn by Image J. Scale bars represent 10 μm. Data in Fig 5A, 5B, 5C, 5D, 5F and 5G are representative of three independent experiments (N = 3 biological replicates).

Next, we aimed to elucidate the molecular mechanism by which A52 blocks the fusion of autophagosomes with lysosomes. Synaptosomal-associated protein 29 (SNAP29) is a member of the SNARE (soluble NSF attachment protein receptor) family and is a pivotal factor that mediates the fusion of autophagosomes with lysosomes. Through interacting with other SNARE proteins such as syntaxin17 (STX17) and VAMP8, SNAP29 brings the membranes of the autophagosome and lysosome into close proximity for the fusion to occur. We hypothesized that A52 blocked the fusion through interacting with SNAP29. To test this hypothesis, the interaction between A52 and SNAP29 was first examined by a co-immunoprecipitation (co-IP) assay using ectopically expressed A52 and SNAP29. A52 was detected in the protein complex pulled down with antibodies specific for SNAP29-Myc (Fig 5A) and the reciprocal co-IP (Fig 5B) also revealed a positive association between the two proteins. To minimize artifacts, the A52 protein expressed by the recombinant virus MVA + A52 was employed as a bait for endogenous SNAP29 in A549 cells. Co-IP analysis confirmed that the endogenous SNAP29 was indeed associated with viral A52 (Fig 5C). Next, we attempted to verify if A52 colocalized with SNAP29 during virus infection. A549 cells were infected with MVA + A52 at 3 PFU/cell and cells were fixed at 12 hpi and stained for A52 with anti-Flag antibody and endogenous SNAP29 with a monoclonal antibody for human SNAP29. Both proteins were observed in the cytoplasm, and the merged image showed a colocalization pattern of the two (Fig 5D).

Human SNAP29 contains two SNARE motifs (aa 60–112 and aa 206–258) divided by a linker region [39]. To map the region responsible for its interaction with A52, we constructed three SNAP29 truncation mutants (Fig 5E) and examined their interaction with A52 using a co-IP analysis described above. The results displayed that deletion of either of the SNARE motifs from SNAP29 completely sabotaged its interaction with A52, while removal of the N-terminal region had no such effect (Fig 5F). In addition, confocal microscopic analysis further confirmed that the truncated mutants of SNAP29 that abolished the protein-protein interaction failed to colocalize with A52 in both A549 (Fig 5G) and HeLa cells (S4A Fig). Next, we generated a truncated version of A52 in which only its BCL-2 like domain was preserved and analyzed its interaction with SNAP29. We found BCL-2 was still able to associate with SNAP29 in a co-IP analysis (S4B Fig).

To further examine if overexpression of SNAP29 was able to overcome the disruption of MAP1LC3B with lysosomes by A52. A549 cells were transfected with SNAP29-Myc at a concentration of 1.5μg/mL for 24h, and then the cells were infected with indicated viruses including WR, WR-ΔA52, vA52-rev, MVA, MVA + A52 at 3 PFU/cell for 12h. Subsequently, the colocalization of MAP1LC3B and lysosomes was observed using confocal microscopy. In the WR-ΔA52 and MVA infection groups, autophagosome–lysosome fusion, as indicated by colocalization of MAP1LC3B and LAMP1, was readily observed and was not affected by SNAP29 overexpression. In contrast, in the WR, vA52-rev, and MVA + A52 infection groups—conditions in which A52 was expressed—colocalization of MAP1LC3B and LAMP1 was markedly disrupted; importantly, this defect was rescued by SNAP29 overexpression (S4C Fig). In MVA-infected A549 cells, colocalization of MAP1LC3B and LAMP1 was observed, whereas suppression of SNAP29 by siRNA disrupted this colocalization (S4D Fig).

Altogether, these data demonstrated SNAP29 may associate with viral A52 through its SNARE motifs and overexpression of SNAP29 was able to alleviate the disruption of MAP1LC3B-lysosome colocalization by A52.

### 2.6 A52 disrupts the association of SNAP29 and its binding partners STX17 and VAMP8

**Fig 6:**
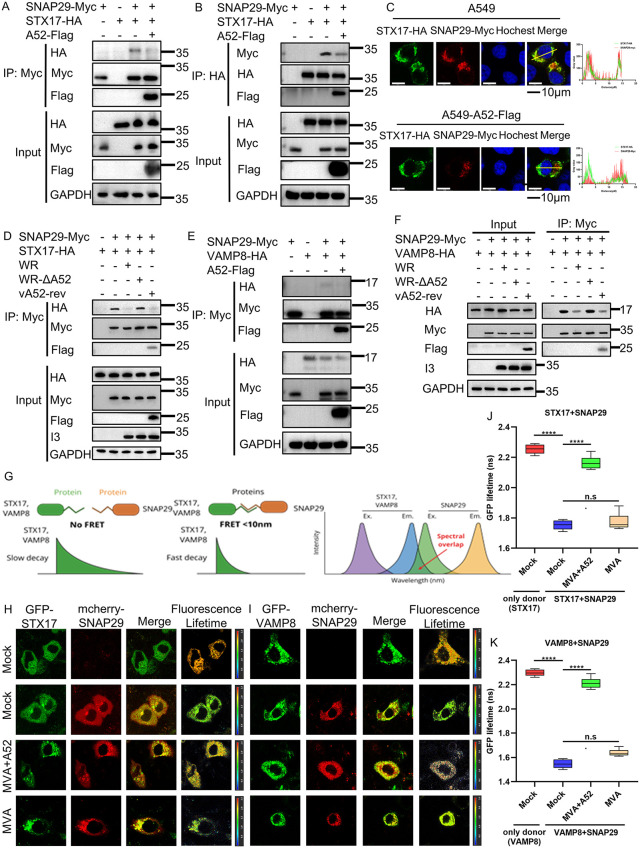
A52 hampered the interaction between SNAP29 and its binding partners STX17 and VAMP8. Human A549 cells were co-transfected with plasmids encoding Myc-tagged SNAP29, HA-tagged STX17, and Flag-tagged A52 for 36 h. Cell lysates were pre-cleared with control magnetic beads and then with Myc- **(A)** or HA-conjugated **(B)** beads at 4˚C for 18 h. Beads were extensively washed and proteins were eluted with SDS loading buffer and resolved by SDS-PAGE and Western blotting analysis. **(C)** A549 cells on coverslips were co-transfected with SNAP29-Myc, STX17-HA and A52-Flag for 24 h. Cells were then fixed, stained with anti-Myc, anti-HA antibodies and Hoechst and images were taken with a fluorescent confocal microscope. The right panels show the fluorescence intensity profile of STX17-HA (green) and Myc-tagged SNAP29 (red) measured along the line drawn by Image J. Scale bars represent 10 μm. **(D)** A549 cells were co-transfected with SNAP29-Myc and STX17-HA for 24 h, and then infected with WR, WR-ΔA52, and vA52-rev for 12 h. Cell lysates were precleared with control magnetic beads and then incubated with Myc-conjugated beads at 4˚C for 18 h. Beads were extensively washed and proteins were eluted with SDS loading buffer and resolved by SDS-PAGE and Western blotting analysis. **(E)** A549 cells were co-transfected with SNAP29-Myc, VAMP8-HA, and A52-Flag for 36 h. Cell lysates were processed as described in (D). **(F)** A549 cells were co-transfected with SNAP29-Myc and VAMP8-HA for 24 h, and then infected with WR, WR-ΔA52, and vA52-rev for 12 h. Cell lysates were processed as described in (D). **(G)** Schematic Diagram of the FRET (Fluorescence Resonance Energy Transfer). **(H)** A549 cells were transfected with GFP-STX17 (donor) and mCherry-SNAP29 (Acceptor) for 24 h, and then were infected with 3 pfu/cell of MVA or MVA + A52 virus for 12 h. FRET-FLIM analysis was performed to assess changes in the donor fluorescence lifetime. Cells co-transfected with GFP-STX17 and mCherry-SNAP29 served as the FRET positive control, while cells transfected with GFP-STX17 alone served as the negative control. The last column displays the fluorescence lifetime of the donor GFP-STX17, pseudo-color coded from low (dark blue) to high (yellow/red). **(I)** A549 cells were transfected with GFP-VAMP8 (donor) and mCherry-SNAP29 (Acceptor) for 24 h, and then were infected with 3 pfu/cell of MVA or MVA + A52 virus for 12 h. FRET-FLIM analysis was performed to assess changes in the donor fluorescence lifetime. Cells co-transfected with GFP-VAMP8 and mCherry-SNAP29 served as the FRET positive control, while cells transfected with GFP-VAMP8 alone served as the negative control. The last column displays the fluorescence lifetime of the donor GFP-VAMP8, pseudo-color coded from low (dark blue) to high (yellow/red). **(J)** Box plots showing median GFP lifetimes in different conditions within the plate: red: only donor (GFP-STX17-GFP); blue: GFP-STX17 (donor) + mCherry-SNAP29 (Acceptor); green: GFP-STX17 (donor) + mCherry-SNAP29 (Acceptor)+MVA + A52; orange: GFP-STX17 (donor) + mCherry-SNAP29 (Acceptor)+MVA. **(K)** Box plots showing median GFP lifetimes in different conditions within the plate: red: only donor (GFP-VAMP8); blue: GFP-VAMP8 (donor) + mCherry-SNAP29 (Acceptor); green: GFP-VAMP8 (donor) + mCherry-SNAP29 (Acceptor)+MVA + A52; orange: GFP-VAMP8 (donor) + mCherry-SNAP29 (Acceptor)+MVA. Data in Fig 6A-F, 6H-I are representative of three independent experiments (N = 3 biological replicates). Statistics: n.s., not significant, ****P < 0.0001 by one-way ANOVA.

The formation of autolysosomes, a critical step in autophagy, is a delicately regulated process that involves the interplay among several cellular components including SNAP29, STX17, VAMP8 and VPS39. SNAP29 serves as an adaptor that binds to STX17 on autophagosomes and VAMP8 on lysosomes, which brings the two vesicles into close proximity for fusion to occur [39]. To investigate if A52 blocks the interaction of SNAP29 with STX17 and VAMP8, the association between the two pairs was first examined in the presence and absence of viral A52 by co-IP analysis. Although ectopically expressed SNAP29 was capable of pulling down STX17 in A549 cells, this interaction was mitigated by the presence of A52 (Fig 6A). Reciprocal co-IP using STX17 as the bait exhibited similar results (Fig 6B). The quantification results of STX17‑HA and SNAP29‑Myc are shown in S5A and S5B Fig, respectively. In addition, results from the confocal microscopic analysis using transfected STX17 and SNAP29 exhibited that the existence of A52 abolished the colocalization between the former two proteins (Fig 6C). To verify if A52 expressed from VACV-WR was able to perform the same function, A549 cells co-transfected with SNAP29-Myc and STX17 were infected with VACV-WR, VACV-WR-ΔA52, or vA52-rev and the association between SNAP29 and STX17 was determined by a co-IP analysis described above. When baiting with SNAP29, the amount of STX17 co-IPed was greatly reduced when cells were infected with WR. However, the reduction was much less prominent in VACV-WR-ΔA52-infected cells and was recovered in vA52-rev-infected cells (Fig 6D). S5C Fig shows the quantification results of STX17‑HA. The difference observed was not due to any difference in virus input as the abundance of viral I3 was comparable between the cells infected with VACV-WR, VACV-WR-ΔA52 or vA52-rev. As SNAP29 also binds to VAMP8, we next examined if A52 was able to interfere with this interaction. As shown in Fig 6E, the presence of A52 evidently attenuated the association between SNAP29 and VAMP8. The quantification results for VAMP8‑HA are presented in S5D Fig. To investigate the effect of A52 expression at physiological levels under authentic viral infection conditions on the interaction between SNAP29 and VAMP8, A549 cells transfected with SNAP29-Myc and VAMP8-HA were infected with VACV-WR, VACV-WR‑ΔA52, or vA52-rev, followed by co‑IP to analyze the SNAP29‑VAMP8 interaction. When baiting with SNAP29, the level of VAMP8 was significantly reduced in VACV-WR‑infected cells. In contrast, no such reduction was observed in VACV-WR‑ΔA52‑infected cells. Importantly, VAMP8 levels were also markedly decreased in cells infected with vA52-rev (Fig 6F). The quantitative results for VAMP8-HA are shown in S5E Fig. Next, we employed FRET (fluorescence resonance energy transfer) analysis to examine whether virus-expressed A52 within an authentic viral context could reproduce the previously observed phenotype. FRET occurs when the emission spectrum of a fluorescent donor molecule (STX17‑GFP or VAMP8‑GFP) overlaps with the excitation spectrum of an acceptor molecule (SNAP29‑mCherry). Upon donor excitation, energy is transferred non-radiatively to the acceptor, resulting in acceptor emission and a concomitant decrease in donor fluorescence intensity. The efficiency of FRET is highly dependent on the spatial proximity of the donor–acceptor pair, occurring effectively within distances <10 nm and decreasing sharply with increasing separation (Fig 6G). Compared to cells transfected only with GFP-STX17 (negative control), cells co-transfected with GFP-STX17 and mCherry-SNAP29 (FRET positive control) exhibited significant FRET signals, with the fluorescence lifetime of the donor GFP-STX17 decreased from 2.29 ns to 1.71 ns, indicating an interaction between STX17 and SNAP29. Additionally, we found that in GFP-STX17/mCherry-SNAP29 co-transfected cells infected with MVA, the fluorescence lifetime of GFP-STX17 was 1.73 ns. Notably, when co-transfected cells were infected with the recombinant MVA + A52 virus, the fluorescence lifetime of GFP-STX17 recovered to 2.24 ns (Fig 6H). This suggests that MVA + A52 inhibited the association between STX17 and SNAP29, whereas MVA alone did not. Similarly, by monitoring changes in the fluorescence lifetime of the donor VAMP8-GFP, we demonstrated that A52 was able to inhibit the interaction between VAMP8 and SNAP29 (Fig 6I). The fluorescence lifetimes of the donors (GFP‑STX17 and GFP‑VAMP8) were shown in Fig 6J–6K. In summary, the presence of A52 correlated with impaired association of SNAP29 with STX17 and VAMP8.

Apart from the SNARE complex, homotypic fusion and protein sorting (HOPS) complex is also known to mediate the fusion of autophagosomes with lysosomes. To analyze if A52 impairs the HOPS complex during autophagy, the interaction between VPS39 and Rab7 was resolved in the presence and absence of A52 using co-IP analysis. As shown in S5F Fig, the interaction between the two HOPS components was weakened when A52 was introduced. The quantitative results for Rab7-Myc are shown in S5G Fig. In addition, results from the confocal microscopic analysis using transfected VPS39 and Rab7 exhibited that the existence of A52 abolished the colocalization between the former two proteins (S5H Fig). These data collectively demonstrate that A52 disrupts the association of SNAP29 with both the SNARE and HOPS complexes. However, it should be noted that this evidence does not directly establish functional impairment of these complexes, representing a limitation in interpreting the mechanistic consequences of this interaction.

To assess whether the observed phenotypes were confounded by nonspecific cellular stress, we examined the activation status of the unfolded protein response (UPR), a canonical indicator of cellular stress. Transfection of A52 did not elevate the mRNA levels of ER stress markers GRP78, GRP94, or CHOP, whereas the positive control, thapsigargin (TG), strongly induced all three transcripts (S5I–S5K Fig). These data suggested that A52 did not induce UPR or ER stress.

### 2.7 VACV-A52 promotes the degradation of SNAP29 via the proteasomal pathway

**Fig 7:**
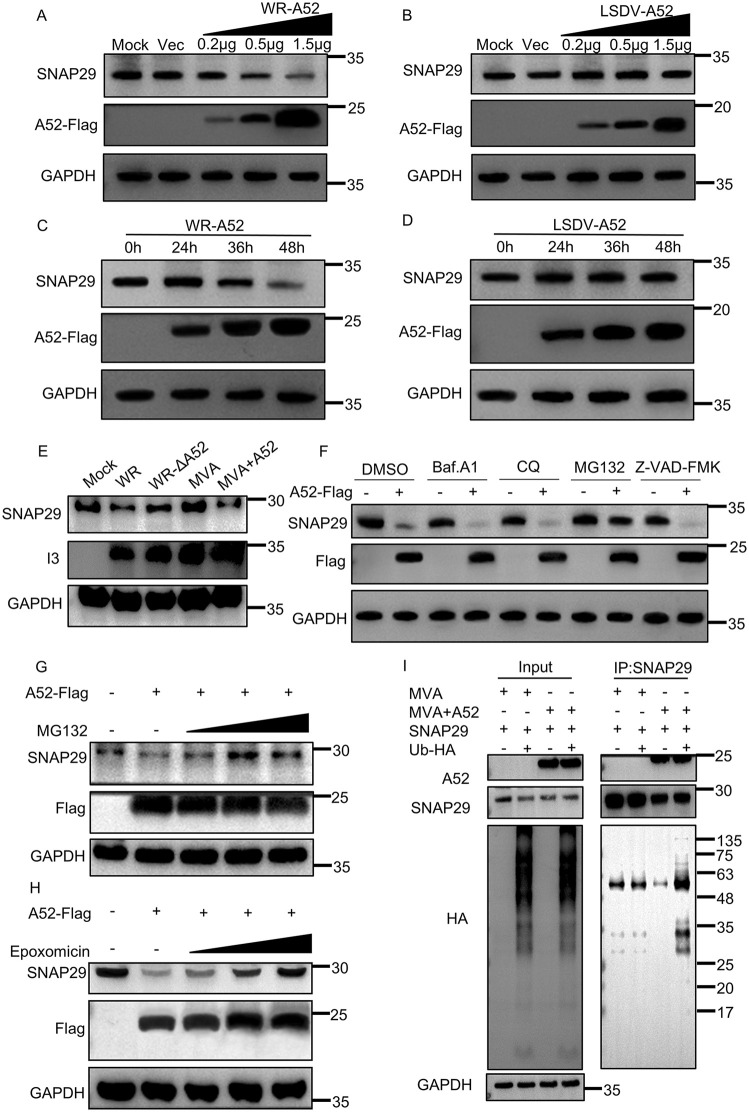
VACV-A52 facilitated proteasome-mediated SNAP29 degradation. A549 cells were transfected with vectors expressing VACV-WR-A52 **(A)** or LSDV-A52 **(B)** at concentrations of 0.2, 0.5, and 1.5 μg/mL for 36 h. Cell lysates were resolved by SDS-PAGE followed by Western blotting analysis with anti-SNAP29, anti-Flag, anti-GAPDH primary antibodies. A549 cells were transfected with plasmids encoding A52 from VACV **(C)** or LSDV **(D)** at 1.5 μg/mL and total proteins were collected at 0, 24, 36, and 48 h post transfection. Protein samples were resolved with SDS-PAGE and Western blotting analysis with primary antibodies for human SNAP29, Flag or GAPDH. **(E)** A549 cells were infected with VACV-WR, VACV-WR-ΔA52, MVA or MVA + A52 for 24 h, and cell lysates were resolved by SDS-PAGE followed by Western blotting analysis with anti-SNAP29, anti-I3, anti-GAPDH antibodies. **(F)** A549 cells were transfected with the A52-Flag or empty vector plasmids for 24 h, and then the cells were treated with Baf. A1 (0.1 μM), CQ (40 μM), MG132 (5 μM) or Z-VAD-FMK (10 μM) for 12 h. DMSO was included as a negative control. Western blotting analysis was conducted to detect endogenous SNAP29 synthesis using protocols described above. **(G-H)** A549 cells were transfected with A52-Flag for 24 h in the presence or absence of MG132 or Epoxomicin at various concentrations. After 12 h, cell lysates were analyzed using SDS-PAGE and Western blotting analysis using anti-SNAP29, anti-Flag, anti-GAPDH antibodies. **(I)** A549 cells were infected with MVA or MVA + A52 at 3 PFU/cell. After 2 h, cells were washed with PBS and then transfected with an empty vector or vector encoding HA-tagged Ubiquitin for 36 h. Cell lysates were then precleared with control magnetic beads and then incubated with SNAP29-conjugated beads at 4˚C for 18 h. Beads were extensively washed and proteins were eluted with SDS-loading buffer and resolved by SDS-PAGE followed by Western blotting analysis with primary antibodies for Flag (A52), SNAP29 or HA (ubiquitin). Data in Fig 7A-I are representative of three independent experiments (N = 3 biological replicates).

As A52 was capable of disrupting the interaction between SNAP29 and its binding partners, STX17 and VAMP8, during autophagy, we sought to investigate whether A52 impacts the stability of SNAP29 during viral infection. For this purpose, the abundance of cellular endogenous SNAP29 was determined by Western blotting analysis when increasing amounts of VACV-A52 or LSDV-A52 were transfected into A549 cells. The endogenous levels of SNAP29 decreased as the amount of VACV-A52 increased (Fig 7A) but remained relatively steady when LSDV-A52 was transfected (Fig 7B). A time-dependent analysis also showed the reduction of SNAP29 in response to only VACV A52, but not LSDV A52 (Fig 7C and 7D). To inspect if SNAP29 was reduced when A52 is expressed by virus, A549 cells were infected with VACV-WR, VACV-WR-ΔA52, MVA or MVA + A52 and the level of endogenous SNAP29 was determined by Western blotting analysis. As shown in Fig 7E, the amount of SNAP29 observed correlated with the presence of A52 and was more abundant in the cells infected with viruses lacking A52 (Fig 7E).

To investigate the nature of the reduction of SNAP29, A549 cells were transfected with a Flag-tagged A52 in the presence or absence of a series of chemical inhibitors targeting different protein degradation pathways, including baf. A1, CQ, MG132 and Z-VAD-FMK. Total protein lysates were collected 36 h post transfection and the level of SNAP29 was resolved by Western blotting analysis using a monoclonal antibody for endogenous SNAP29. In agreement with the previous observation, introduction of A52 resulted in an observable reduction of endogenous SNAP29. While the treatment of baf. A1, CQ and Z-VAD-FMK had minimal effect on the level of SNAP29, the addition of MG132 remarkably ameliorated the depression of SNAP29 caused by A52 (Fig 7F), insinuating that the reduction of SNAP29 might be the result of protein degradation via the proteasomal pathway. A dose-dependent experiment was performed and the results further confirmed that the addition of MG132 led to the rescue of A52-mediated SNAP29 degradation (Fig 7G). An alternative proteasome inhibitor, epoxomicin, exhibited a similar dose-dependent rescue of the level of SNAP29 (Fig 7H). To further corroborate this conclusion, the ubiquitination of SNAP29 was inspected in cells infected with MVA or MVA + A52 (Fig 7I). Compared with MVA, the ubiquitination level of SNAP29 was greater in cells infected with MVA + A52 (Fig 7I). These results demonstrated that A52 enhanced the ubiquitination of SNAP29 and the subsequent degradation via a proteasome-dependent mechanism.

### 2.8 SNAP29 is a host restriction factor for VACV lacking A52

**Fig 8:**
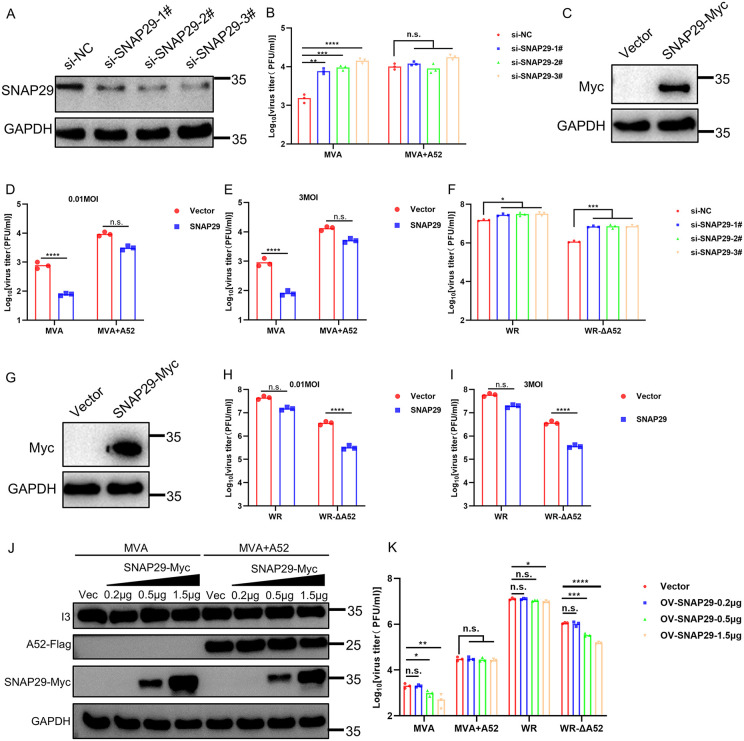
SNAP29 inhibits the replication of MVA in A549 cells. Human A549 cells were transfected with siNC or siSNAP29 **(A)** for 48 h and then infected in triplicates with MVA or MVA + A52 **(B)** and WR or WR-ΔA52 **(F)** at 3 PFU/cell. Viruses were harvested at 24 hpi and virus titers were determined by plaque assay on DF-1 cells or BS-C-1 cells. Human A549 cells were transfected with SNAP29-Myc **(C)** for 24 h and then infected in triplicates with MVA or MVA + A52 at 0.01 **(D)** or 3 PFU/cell **(E)**. Viruses were collected at 48 or 24 hpi and titers were determined by plaque assay on DF-1 cells. Human A549 cells were transfected with SNAP29-Myc **(G)** for 24 h and then infected in triplicates with WR or WR-ΔA52 at 0.01 **(H)** or 3 PFU/cell **(I)**. Viruses were collected at 48 or 24 hpi and virus titers were determined by plaque assay on BS-C-1 cells. **(J-K)** A549 cells were transfected with SNAP29-Myc at concentrations of 0.2, 0.5, or 1.5 μg/mL for 24 h and were infected in triplicate with MVA, MVA + A52, VACV-WR or VACV-WR-ΔA52 at 3 PFU/cell for 24 h and virus titers were determined by plaque assay on DF-1 or BS-C-1.(Data in Fig 8B, 8D, 8E, 8F, 8H, 8I, and 8K are shown as dots, and the bar represents the mean value. Data in Fig 8A-K are representative of three independent experiments (N = 3 biological replicates). Statistics: n.s., not significant, p > 0.05; *P < 0.05; **P < 0.01; ***P < 0.001; ****P < 0.0001 by two-sided Student’s t test.

MVA is replication-deficient in the majority of mammalian cells and almost all human cells [6]. Previous reports illustrated that zinc-finger antiviral protein (ZAP) and FAM111A contributed to the restriction of MVA in human cells [9,10]. As our data demonstrated that VACV-WR was capable of blocking autophagosome-lysosome fusion while MVA was unable to do so, we hypothesized that the inability to block the formation of autolysosome might also contribute to the restriction of MVA in human cells. To test this hypothesis, we manipulated the synthesis of SNAP29 by small interfering RNA (siRNA) and examined the replication of MVA and recombinant MVA + A52 in these cells compared to the control cells, in which siNC was transfected. Transfection of different pairs of siSNAP29 led to distinct reduction in SNAP29 protein in A549 cells (Fig 8A) and resulted in an increase of MVA titers up to ~10 folds (Fig 8B). Nevertheless, the effect of SNAP29 depression was much less prominent for the replication of MVA + A52 as the recombinant virus expressing A52 already showed a ~ 10 folds enhancement in virus replication (Fig 8B). To ensure the effect on virus replication was due to SNAP29 but not the off-target effect of siSNAP29, A549 cells were transfected with SNAP29 (Fig 8C) prior to infection with MVA or MVA + A52 and viral titers were monitored. Ectopic expression of SNAP29 greatly reduced the replication of MVA (~15 folds) but had a slight effect on that of MVA + A52 (~3 folds) (Fig 8D and 8E).

Next, we analyzed the consequence of SNAP29 suppression on the replication of VACV-WR and VACV-WR-ΔA52. The loss of A52 resulted in a ~ 10 folds decline in virus titer, but the reduction was rescued by suppressing SNAP29 expression via siSNAP29 (Fig 8F). In addition, ectopically expressed SNAP29 caused a 15 folds decrease of the titers of VACV-WR-ΔA52 but had an insignificant impact on the replication of VACV-WR (Fig 8G–8I).

To confirm the effect of SNAP29 on viral replication, a dose-dependent transfection of SNAP29 was performed in A549 cells infected with the viruses indicated above (Fig 8J). The results confirmed a decrease of viral replication for those that lack A52 in response to increased levels of SNAP29 (Fig 8K). In addition, SNAP29 dose-dependently reduced the replication of MVA but had no effect on that of MVA + A52 (Fig 8K). Overall, these data demonstrated that SNAP29 is one of the host restriction factors for viruses that lack A52 but had minimal effect on viruses that express A52.

## 3. Discussion

**Fig 9:**
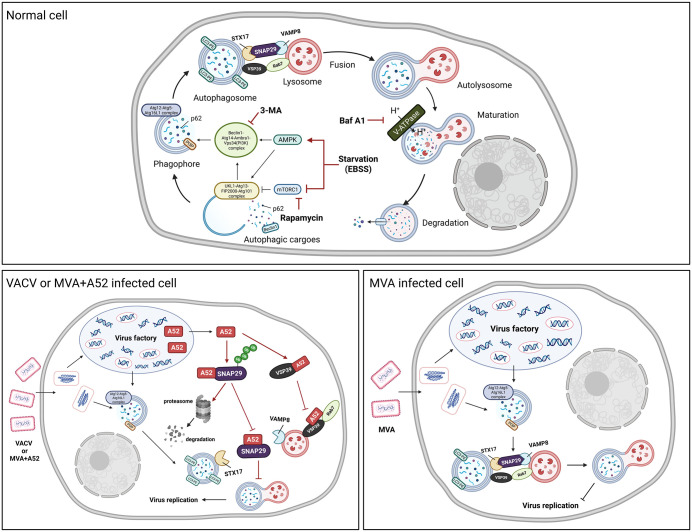
Mechanism of A52 in modulating the fusion of autophagosome and lysosome. The figure was *created in BioRender. Deng, Y. (2026)* *https://BioRender.com/e0uikcg*.

Autophagy is a core cellular process that maintains homeostasis by degrading damaged components and invading pathogens [13]. Viruses frequently manipulate this pathway to promote their own replication, making autophagy a critical interface in host–virus interactions. In this study, we demonstrate that modulation of autophagy significantly influences VACV replication and identify a molecular mechanism by which the VACV protein A52 inhibits autophagosome–lysosome fusion, resulting in incomplete autophagy (summarized in Fig 9).

Previous studies have suggested that VACV replication can be largely independent of canonical autophagy initiation factors, such as ATG5 and BECN1 [32], and that VACV selectively subverts xenophagy through mechanisms involving SQSTM1 phosphorylation and mTOR signaling regulation [33,34]. Consistent with these reports, we found that pharmacological inhibition of late-stage autophagy enhanced the replication of MVA in non-permissive human cells but had minimal effects on VACV-WR replication. These results support a model in which early autophagic processes may benefit poxvirus replication, whereas completion of autophagy becomes restrictive. Early autophagy may provide metabolic resources or dampen antiviral signaling, while prolonged or complete autophagic flux could degrade viral components or disrupt cellular conditions favorable for replication.

Genetic perturbation of autophagy-related genes further highlighted the selective and modular nature of poxvirus–autophagy interactions. Individual knockdown of ATG3, ATG7, or ATG12 did not affect viral replication, whereas depletion of ATG16L1 or BECN1 significantly reduced viral titers. Importantly, combined knockdown of ATG7 with ATG16L1 or BECN1 did not further decrease replication beyond that observed with ATG16L1 or BECN1 knockdown alone, indicating that VACV depends on specific autophagy modules rather than the entire pathway. This modular dependence is consistent with observations from other viruses, which selectively exploit discrete stages of autophagy for replication or immune evasion [40–54]. The differential effects observed for ATG12 and ATG16L1 may reflect their specialized roles within the ATG5–ATG12–ATG16L1 complex, which drives MAP1LC3B-I lipidation and autophagosome membrane elongation. It is conceivable that poxviruses encode viral factors capable of functionally compensating for the loss of certain ATGs, potentially mimicking ubiquitin-like conjugation activities [55–59]. While this hypothesis remains speculative, it provides a plausible explanation for the limited impact of ATG7 or ATG12 depletion on poxvirus replication.

Time-of-addition experiments with bafilomycin A1 revealed that inhibition of VACV replication occurred only when treatment coincided with viral entry, likely due to impaired endosomal acidification rather than autophagy inhibition per se. In contrast, late-stage bafilomycin A1 treatment restored MVA replication in non-permissive cells, phenocopying the effect of A52 expression. These findings suggest that VACV actively promotes autophagosome accumulation while preventing their fusion with lysosomes, thereby avoiding autophagic degradation. Our data identify A52 as the viral factor responsible for this blockade.

Mechanistically, these findings suggest that A52 may impair autophagosome–lysosome fusion by disrupting the SNARE complex (STX17–SNAP29–VAMP8) and HOPS–Rab7 tethering machinery, although direct causality remains to be established with further experimental evidence. Through confocal microscopy, co-immunoprecipitation, and FRET analyses, we demonstrate that A52 binds directly to SNAP29 via its SNARE motifs, impairing SNAP29’s interaction with STX17 and VAMP8 and promoting SNAP29 degradation via the proteasome. This dual targeting effectively blocks cargo degradation and leads to autophagosome accumulation. Similar strategies have been described for other viruses [60–65], underscoring autophagic flux inhibition as a conserved viral strategy.

A key finding of this study is the distinct difference between VACV-WR and MVA in their capacity to interrupt autophagosome–lysosome fusion. Comparative genomics and functional analyses identified A52 as a candidate mediator of this differential phenotype. Introduction of A52 into MVA disrupted the association between SNAP29 and its binding partners and was concomitant with enhanced MVA replication in human cells, underscoring a potential link between late-stage autophagy inhibition and poxvirus replication efficiency. Preliminary screening suggests that other viral proteins may also have effects; however, their precise functions and effect strengths require confirmation through more in-depth future studies.

Our results further establish SNAP29 as a host restriction factor for MVA. Overexpression of SNAP29 inhibited replication of A52-deficient viruses, whereas SNAP29 depletion enhanced replication of MVA and VACV-ΔA52 but had little effect on VACV-WR or MVA + A52. These findings place SNAP29 alongside previously identified MVA restriction factors, such as ZAP and FAM111A[9,10], and provide new insight into the molecular basis of MVA’s limited host range.

A52 is also known as a BCL-2–like immunomodulatory protein that suppresses NF-κB signaling downstream of multiple Toll-like receptors [66–68]. Our data extend A52’s functional repertoire by demonstrating a NF-κB–independent role in autophagy regulation. Infections with VACV-WR or MVA + A52 induced MAP1LC3B accumulation and SQSTM1 stabilization while preventing MAP1LC3B–LAMP1 colocalization, consistent with incomplete autophagy. Together, these findings suggest that A52 may contribute to viral replication through its association with disrupted autolysosome formation, although the precise mechanism warrants further investigation. In summary, this study identifies A52 as a conserved poxviral antagonist of autophagosome–lysosome fusion and establishes SNAP29 as a host restriction factor targeted by poxviruses. These findings highlight late-stage autophagy inhibition may be associated with poxvirus replication efficiency and host range. Future studies addressing the structural basis of A52 function and its potential interactions with additional fusion regulators may facilitate the development of targeted antiviral strategies.

### 3.1 Limitations of the study

1. The precise mechanism by which early autophagy promotes VACV replication remains unknown, and the data presented here do not provide definitive evidence.
2. The viral components that may compensate for the loss of ATG7, ATG12, or ATG3 were not identified in this study and require further investigation.
3. This study uses SQSTM1 levels as an indicator of autophagy activation. However, it is important to note that SQSTM1 is subject to complex, multi-layered regulation, including transcriptional and translational control, as well as degradation through non-autophagic pathways. Consequently, reliance on SQSTM1 levels alone is insufficient to accurately reflect autophagic flux, and this represents a limitation of the current study.
4. Co-immunoprecipitation and FRET-based proximity assays primarily reflect changes in protein association or spatial proximity, and do not by themselves demonstrate functional disruption of the SNARE complex. Therefore, additional functional evidence is required to substantiate impaired SNARE complex activity.
5. Although we have demonstrated that A52 does not activate the canonical UPR, we cannot completely rule out indirect effects mediated by other untested cellular stress pathways.

## 4. Materials and methods

### 4.1 Cell lines, plasmids, and viruses

A549 cells (Mingzhoubio, cat#MZ-0015, RRID:CVCL_0023), HeLa cells (Mingzhoubio, MZ-3329, RRID:CVCL_0030) and BS-C-1 cells (Mingzhoubio, cat#MZ-8048, RRID:CVCL_0607) were purchased from Ningbo Ming Zhou bio CO., Ltd. BHK-21[C-13] (Procell, cat#CL-0034, RRID:CVCL_1914), DF-1 (Procell, cat#CL-0279, RRID:CVCL_0570) were kindly provided by Procell Life Science&Technology Co., Ltd. Establishment of A549 cells stably expressing GFP-MAP1LC3B was performed as follows. A549 cells were infected with Lentivirus containing GFP-MAP1LC3B (GV616, Genechem, Shanghai, China) for 4 h and then culture medium was removed and supplemented with fresh medium. Cells were then treated with 2.5μg/mL puromycin continuously for 7 days. The expression of MAP1LC3B was identified by WB analysis and fluorescent confocal microscopic analysis. All cells were cultured at 37°C with 5% CO_2_ in Dulbecco’s modified Eagle’s medium (DMEM) supplemented with 10% (vol/vol) fetal bovine serum (FBS), 100 units of penicillin/mL, 100 µg/mL of streptomycin.

ORFs of SNAP29, STX17, VAMP8, VPS39 and Rab7 were cloned from A549 cDNA into a pcDNA3.1 vector (cat#V790-20, Invitrogen). Genes from VACV-WR were codon-optimized and cloned into the pcDNA3.1 vector. Ub-HA plasmid was purchased from MIAOLING PLASMID (cat#P37795).

VACV-WR and MVA were kindly provided by Bernard Moss from the National Institutes of Health.

### 4.2 Construction of recombinant MVA + A52, VACV-WR-ΔA52 and vA52-REV

Recombinant MVA + A52 was constructed by homologous recombination using fluorescent reporter genes (EGFP) for plaque selection. Briefly, a DNA cassette containing the A52 ORF regulated by an mH5 (modified H5) promoter followed by an EGFP ORF regulated by the P11 promoter was generated. The cassette containing mH5 + A52 + P11 + EGFP was inserted into the intergenic locus between MVA 069 and 070 by homologous recombination [8]. DF-1 cells were infected with parental MVA at 3 PFU/cell and then transfected with the DNA cassette described above. Viruses were harvested at 24 hpi and recombinant viruses were selected by selecting eGFP-positive clones. Positive clones were plaque-purified five times in DF-1 cells. Viral DNA was isolated from the clones with the DNeasy Blood & Tissue kit (Qiagen) and sequences were verified by PCR followed by Sanger sequencing. VACV-WR-ΔA52 recombinant virus was generated as follows. A P11-directed eGFP sequence was cloned into a plasmid and flanked by 500 bp up- and downstream of the A52 ORF. The 1552-bp PCR product was inserted into pcDNA-3.1 vector and was used to transfect HeLa cells infected with VACV-WR at 3 PFU/cell for 2 h. A recombinant virus lacking A52 was isolated by selecting eGFP-positive virus clones and clonally purified by repeated plaque isolation. The loss of the A52 gene was confirmed by PCR and Sanger sequencing.

vA52-REV was generated by homologous recombination using fluorescent reporter genes (mCherry) for plaque selection. Briefly, a DNA cassette containing the A52 ORF regulated by an mH5 (modified H5) promoter followed by an mCherry ORF regulated by the P11 promoter was generated. The cassette containing mH5 + A52 + P11 + mCherry was inserted into the intergenic locus between WR-ΔA52 177 and 179 by homologous recombination. HeLa cells were infected with WR-ΔA52 at 3 PFU/cell and then transfected with the DNA cassette described above. Viruses were harvested at 24 hpi and a recombinant virus complemented with A52 was selected by selecting mCherry-positive clones and clonally purified by repeated plaque isolation in BHK-21 cells.

### 4.3 Antibodies and chemicals

Rabbit anti-SNAP29 polyclonal antibody (12704–1-AP) was purchased from Proteintech. Rabbit anti-MAP1LC3B antibody (L7543) was purchased from Sigma. Rabbit anti-SQSTM1 (ab155686) was purchased from Abcam. Anti-LAMP1 antibody (#9091) was purchased from Cell Signaling Technology. Autophagy Antibody Sample Kit (4445T) was purchased from Cell Signaling Technology. Lyso-Tracker Red (C1046), anti-Flag mouse monoclonal antibody (AF2852), anti-Myc rabbit monoclonal antibody (AM933), anti-HA mouse monoclonal antibody (AF2858), BeyoMag Anti-Flag Magnetic Beads (P2115), BeyoMag Anti-HA Magnetic Beads (P2121) and BeyoMag Anti-Myc Magnetic Beads (P2118) were purchased from Beyotime. Rapamycin (Rapa.) (S1842) was purchased from Beyotime. MG132 (S2619), Epoxomicin (S7038), Bafilomycin A1 (baf. A1) (S1413) and 3-MA (3-Methyladenine) (S2767) were purchased from Selleck. Chloroquine diphosphate was kindly provided by Professor Kui Zhu from China Agricultural University.

### 4.4 Virus infection and plaque assay

A549 or DF-1 cells in 6-well plates were cultured to reach ~90% confluency. Medium was removed and replaced with inoculum containing various viruses in complete DMEM supplemented with 2.5% FBS to reach a titer of 0.01 or 3 PFU/cell. After 2 h incubation at 37˚C, the inoculum was removed, and cells were washed twice with 1 × PBS and then supplemented with fresh medium. Total viruses were harvested at 48 hpi or 24 hpi by cell scraping and repeated freeze-thaw cycles. Virus titers were determined by plaque assay in BS-C-1 or DF-1 cells described previously [9].

### 4.5 Quantitative realtime-PCR

A549 cells in 6-well plates were infected with viruses indicated in S1I Fig at 3 PFU/cell for 1 h in triplicate. Cells were washed twice with 1 × PBS and total DNA was extracted at 6 hpi with a DNeasy Blood/Tissue DNA mini kit (Qiagen) and total DNA yield was measured by a nanodrop spectrophotometer (Thermo Fisher Scientific). Equal amounts (1µg) of DNA from each sample were then serially diluted and subjected to quantitative realtime-PCR with gene-specific primers for VACV E11 and host GAPDH as the housekeeping control. The results of genomic DNA virus were normalized to that of GAPDH, and relative DNA abundance was calculated and shown in S1I Fig. A549 cells in 6-well plates were infected with viruses at 3 PFU/cell in triplicates. Total RNA was extracted at 2 or 8 hpi with RNAprep pure Cell/Bacteria kit (TIANGEN, DP430) according to the manufacturer’s instructions, and quantitated by a nanodrop spectrophotometer. Equal amounts (1µg) of RNA were reverse transcribed using a EasyScript First-Strand cDNA Synthesis SuperMix kit (TRANSGEN, AE301–02) and subjected to quantitative Realtime-PCR with gene-specific primers for virus E3, D13, A3 and host GAPDH as the housekeeping control.

### 4.6 Proteomic sample preparation and analysis

A549 cells were infected with VACV-WR at 3 PFU/cell for 2 hours. Subsequently, the cells were either treated with 0.1 μM bafilomycin A1 (Baf. A1) or left untreated. cells were harvested at 24 hpi, and total proteins were extracted. Protein samples were subjected to enzymatic digestion, and viral protein abundance was quantitatively assessed by liquid chromatography-tandem mass spectrometry (LC-MS/MS). Subsequent proteomic analysis was performed to evaluate VACV protein expression profiles under the respective experimental conditions. We performed the analysis using an Orbitrap Fusion Lumos mass spectrometer, with Spectronaut as the search engine and a false discovery rate (FDR) threshold set at 1%.

### 4.7 Confocal microscopy

Monolayers of A549 cells were grown on glass coverslips in 24-well plates and then infected with viruses indicated in S2C, 4A, 4G, and S3A Figs for the indicated time. Medium was removed and cells were fixed with ice-cold 4% paraformaldehyde for 20 min at room temperature, followed by incubation with 0.1% Triton X-100 for 20 min and blocked with 3% BSA diluted in 1 × PBS for 30 min. LAMP1 antibody (1:200) and MAP1LC3B antibody (1:200) were added and incubated with cells at 4 ºC overnight. Cells were then washed with 3% BSA three times, followed by incubation with secondary antibodies conjugated with Alexa Fluor 488 or Alexa Fluor 555 or Alexa Fluor 647 at 37 ºC in dark with agitation for 1 h. Hoechst was used to stain the nucleus for 5 min. Cells were washed multiple times before mounting on slides using ProLong Diamond Antifade reagent (Thermo Fisher Scientific). Images were taken on a Leica SP8 confocal microscope and images were processed with the Leica software (Leica Biosystems). The fluorescence intensity profile of images was quantified using Image J software. Three independent biological replicates were performed, with 15 cells quantified per replicate.

All image acquisition parameters were fixed and uniformly applied to all experimental conditions in every imaging-based experiment. Specifically, key acquisition parameters (including exposure time, gain, laser power, objective lens, scan resolution, etc.) were standardized before the experiment began, and no adjustments were made between different experimental groups or between biological replicates. This ensures that any differences observed in the image signals are due to genuine biological changes, rather than fluctuations in acquisition parameters. Specific parameters are as follows: Leica SP8; 100X oil immersion objective lens; Frame Size: 1024 × 1024; Speed: 400; Zoom Factor: 4.00; seq1: Wavelength: 405nm; Gain(V): 900; Offset [%]: 0; seq2: Wavelength:488; Gain(V): 900; Offset [%]: 0; seq3: Wavelength: 552nm; Gain(V): 500; Offset [%]: 0.

In this study, all image processing and quantitative analysis for the tandem fluorescent reporter system (mRFP-GFP-LC3) and LC3-LAMP1 co-localization experiments were performed using ImageJ (Fiji) software. The analysis parameters were pre-defined, fixed parameters that remained consistent across all experimental conditions. This parameter set was established prior to data collection and was rigorously applied to all experimental groups. The specific analysis steps are as follows:

Open the Analyze→Tools→ROI Manager, select the region of interest in the mCherry or GFP channel. Convert the image to 8‑bit (Image→Type→8‑bit) and adjust the threshold (1.61%, 47, 299) to identify punctate structures in each channel. Apply Process→Binary→Fill Holes followed by Watershed to separate adjacent puncta. Use Analyze Particles with the size set to 0.66–infinity to count the red or green fluorescent puncta in each channel.

To quantify yellow puncta (representing colocalized mCherry and GFP signals), the two binary images (mCherry and GFP) were combined using the “AND” operation. Puncta were defined as “yellow” only if the overlap between mCherry and GFP signals exceeded 50% in area.

To further eliminate potential subjective bias during image processing and quantitative analysis, all fluorescence images in this study were analyzed by two independent researchers using a blinded method (the analysts were unaware of the experimental group assignments of the samples). The quantitative results from the two researchers showed a high degree of consistency (Pearson correlation coefficient r > 0.95).

### 4.8 Co-immunoprecipitation assay

Human A549 cells were co-transfected with vectors encoding Myc-tagged SNAP29, Rab7, HA-tagged STX17, VAMP8 and VPS39 and Flag-tagged A52 for 36 h. Cells were first washed once with ice-cold 1 × PBS and lysed in wells with 150 µL IP lysis buffer with 1 × PMSF (Beyotime Biotechnology) on ice. Protein lysates were collected and centrifuged for 10 min at 12,000 × g at 4 ºC and then precleared with control magnetic beads for 2h. Supernantant was collected and incubated with HA beads (Beyotime), Myc-Beyotime) or Flag-Beads (Beyotime) for overnight at 4 °C with rotation. The beads were then washed 6× times with 500μL 1 × TBS buffer and the bound proteins were eluted by addition of 100μl 1 × SDS-PAGE loading buffer containing 0.05M DTT and by boiling for 15 min. Supernatant was taken for SDS-PAGE followed by Western blotting analysis.

### 4.9 Fluorescence resonance energy transfer-fluorescence lifetime imaging microscopy (FRET-FLIM) analysis

A549 cells were seeded 1 day before transfection in a four-chamber glass-bottomdish (SHANG HAI JING AN). A549 cells were transfected with GFP-STX17/GFP-VAMP8 (donor) and mCherry-SNAP29 (acceptor) for 24 h, and then were infected with 3 pfu/cell of MVA or MVA + A52 virus for 12 h. Cells co-transfected with GFP-STX17 and mCherry-SNAP29 served as the FRET positive control, while cells transfected with GFP-STX17 alone served as the negative control. Images were acquired using fluorescence lifetime imaging microscopy (Leica SP8). For imaging, a 60 × oil‑immersion objective was used, and images were acquired at a resolution of 512 × 512 pixels. FLIM measurements were conducted with an 80 MHz pulsed laser and detected using a HyD SMD single‑photon detector. The following excitation and detection settings were applied: GFP was excited at 488 nm and collected through a 525/50 nm bandpass filter; mCherry was excited at 561 nm and collected through a 610/60 nm bandpass filter. Data were analyzed with LAS X FLIM software. In the FLIM images, a uniform region with high signal‑to‑noise ratio was selected as the region of interest (ROI). Fluorescence lifetime decay curves were fitted using an n‑exponential reconvolution model, and the quality of fitting was evaluated based on the χ² value and the distribution of residuals. Changes in donor fluorescence lifetime across experimental groups were compared statistically. The last column displays the fluorescence lifetime of the donor GFP-STX17/GFP-VAMP8, pseudo-color coded from low (dark blue) to high (yellow/red).

### 4.10 Statistical analysis

All experiments were performed with triplicated samples for each group or treatment, and each experiment was independently repeated three times (N indicates genuine biological replicates, but not technical replicates, N = 3). Statistical significance among groups was assessed using two-way analysis of variance (ANOVA) in GraphPad Prism (version 6.0). A *P* value < 0.05 was considered statistically significant, whereas *P* > 0.05 was considered not significant. Circular heat maps were generated using ChiPlot (https://www.chiplot.online/).

## Supporting information

S1 Fig Differential effects of autophagy inhibitors and autophagy-related genes on VACV replication. (A-B) A549 cells were treated with rapa. or baf. A1 at the concentrations mentioned above for 24 h and cell viability was measured by an MTT assay. (C-D) DF-1 cells were treated with rapa. or baf. A1 at the concentrations mentioned above for 24 h and cell viability was measured by an MTT assay. (E) A549 cells were treated with 3-MA at the concentrations mentioned above for 24 h and cell viability was measured by an MTT assay. (F-I) A549 cells were infected with VACV-WR in the presence of AraC (40 mg/mL), rapa. (100nM) or baf. A1 (0.1μM) for 2 h or 8 h and total RNA and DNA were harvested for mRNA and viral genomic DNA quantification using quantitative realtime-PCR with VACV-specific primers.(J-N) A549 cells were transfected with si-NC, si-ATG12 (J), si-ATG16L1(K), si-BECN1(L), si-ATG7 + si-ATG16L1 (M), and si-ATG7 + si-BECN1 (N) for 48 h and then were infected with the VACV-WR at an MOI of 3. After 24 hpi, virus titers were determined by a plaque assay. P-values were calculated using the one-way ANOVA; ns, not significant; ***P < 0.001; ****P < 0.0001. All experiments were performed in triplicate (N = 3 biological replicates).
(TIF)

S2 Fig VACV, but not MVA infection can block the fusion of autophagosome with lysosome in Permissive cell. (A) HeLa cells were transfected with mCherry-GFP-MAP1LC3B for 24 h and then were mock infected or infected with WR, MVA at 3 PFU/cell or treated with CQ or rapa. for 12 h, and then cells were fixed, permeabilized, blocked, stained with Hoechst and analyzed with a fluorescent confocal microscope. Scale bar, 10 μm. (B) The graph shows the quantification of autophagosomes by taking the average number of dots in 15 cells. (C) A549 cells stably expressing MAP1LC3B on cover slips were infected with VACV-WR, MVA and at 3 PFU/cell or treated with baf. A1 (0.1μM) or rapa. (100nM). At 12 hpi, cells were then fixed, permeabilized, blocked, and stained with primary antibodies to LAMP1 and followed by fluorescent conjugated secondary antibodies. Hoechst was used to stain nucleus. Scale bars represent 10 μm. (D) Images were taken as described above and quantification of colocalization was analyzed by Image J software in 15 randomly selected cells. Data in S2A-S2D Fig are representative of three independent experiments (N = 3 biological replicates).
(TIF)

S3 Fig Identification of viral proteins responsible for inhibiting the fusion of autophagosomes and lysosomes. (A) Human A549 cells that stably express GFP-MAP1LC3B were transfected with plasmids of all candidate genes prior to MVA infection at 3 PFU/cell. At 12 hpi, cells were fixed, permeabilized, blocked, and stained with primary antibodies to LAMP1 and followed by fluorescent conjugated secondary antibodies. Hoechst was used to stain nucleus. Scale bar, 10 μm. (B-C) Human A549 cells were infected with 3PFU/cell WR or MVA, after 2 h, cells were transfected with plasmids of all candidate genes, and cell lysates were collected for Western blot analysis with anti-SQSTM1, anti-MAP1LC3B, anti-Flag, anti-GAPDH antibodies. Data in S3 A-S3C Fig are representative of three independent experiments (N = 3 biological replicates).
(TIF)

S4 Fig A52 interacts with human SNAP29. (A) HeLa cells grown on coverslips were transfected with vectors encoding Myc-tagged SNAP29 or its mutants, and a Flag-tagged A52 for 24 h. (The transfection doses of each plasmid were 0.5 μg). Cells were then fixed, permeabilized, blocked, and stained with primary antibodies to Myc or Flag and followed by fluorescent conjugated secondary antibodies. Hoechst was used to stain nucleus. Scale bars represent 10 μm. (B) A549 cells were co-transfected with vectors encoding Flag-tagged A52 or its mutants, and a Myc-tagged SNAP29 for 36 h. Cell lysates were pre-cleared with control magnetic beads or Flag-conjugated beads at 4˚C for 18 h followed by extensive washing. Proteins were eluted with SDS loading buffer and resolved by SDS-PAGE followed by Western blotting analysis using primary antibodies for Flag, Myc and GAPDH. (C) A549 cells were transfected with SNAP29-Myc at a concentration of 1.5μg/mL for 24h, and then the cells were infected with indicated viruses including WR, WR-ΔA52, vA52-rev, MVA, MVA + A52 at 3 PFU/cell for 12h. Cells were then fixed, permeabilized, blocked, and stained with primary antibodies to LAMP1 or MAP1LC3B and followed by fluorescent conjugated secondary antibodies. LAMP1 was used to stain lysosomes. MAP1LC3B was used to stain autophagosome. Hoechst was used to stain nucleus. Scale bars represent 10 μm. (D) A549 cells were transfected with siNC or siSNAP29 for 48 h and then infected in triplicates with MVA or MVA + A52 at 3 PFU/cell for 12h. Cells were then fixed, permeabilized, blocked, and stained with primary antibodies to LAMP1 or MAP1LC3B and followed by fluorescent conjugated secondary antibodies. LAMP1 was used to stain lysosomes. MAP1LC3B was used to stain autophagosome. Hoechst was used to stain nucleus. Scale bars represent 10 μm. Data in A-D are representative of three independent experiments (N = 3 biological replicates).
(TIF)

S5 Fig Quantitative densitometry for co-IPs and A52 inhibits the interaction between VPS39‑HA and Rab7‑Myc. (A)The quantification of STX17-HA. (B) The quantification of SNAP29-Myc. (C) The quantification of STX17-HA. (D-E) The quantification of VAMP8-HA. (F) A549 cells were co-transfected with Rab7-Myc, VPS39-HA and Flag tagged A52 from VACV. Cell lysates were pre-cleared with control magnetic beads and then incubated with HA-conjugated beads at 4˚C for 18 h. Beads were extensively washed, and proteins were eluted with SDS loading buffer and resolved by SDS-PAGE and Western blotting analysis. (G) The quantification of Rab-Myc. (H) A549 cells on coverslips were co-transfected with Rab7-Myc, VPS39-HA and A52-Flag for 24 h. Cells were then fixed, stained with anti-Myc, anti-HA antibodies and Hoechst and images were taken with a fluorescent confocal microscope. The right panels show the fluorescence intensity profile of VPS39-HA (green) and Myc-tagged Rab7 (red) measured along the line drawn by Image J. Scale bars represent 10 μm. (I-K) A549 cells were transfected with A52-Flag. Cells treated with TG (5 μM) for 4 h served as positive controls. Total RNA was harvested at 36 hours post-transfection, and the relative expression of GRP78 (I), GRP94 (J), CHOP (K) was detected by qPCR. Data in S5A-S5K Fig are representative of three independent experiments (N = 3 biological replicates).
(TIF)

S1 Table The main targets of Baf.A1. (DOCX)

S2 Table The dataset used to build images in this article. (XLSX)

S1 Data The raw data of western blot and IF in this study. (PDF)

S2 Data Proteomics. (XLSX)

S3 Data Sequencing results of different viral genes. (RAR)

## Abbreviations

Baf.A1: bafilomycin A1
3-MA: 3-methyladenine
ATG16L1: autophagy related 16 like 1
Rapa.: rapamycin
CQ: chloroquine
MAP1LC3B: microtubule associated protein 1 light chain 3B
hpi: hour post-infection
siRNA: small interfering RNA
SNAP29: synaptosome associated protein 29
SQSTM1: sequestosome 1
STX17: syntaxin 17
VAMP8: Vesicle associated membrane protein 8
VPS39: Vps39-like protein
Rab7: Ras-related protein Rab-7a
NC: negative control
VACV: vaccinia virus
MVA: modified vaccinia virus Ankara
Mpox: Monkeypox
