# Supplementary figures and images for: Poxvirus A52 protein subverts autophagy flux by blocking autophagosome–lysosome fusion to promote viral replication
Source: PLoS Pathog. 2026 Apr 13;22(4):e1014137. doi: 10.1371/journal.ppat.1014137 (PMC13075716; doi:10.1371/journal.ppat.1014137)

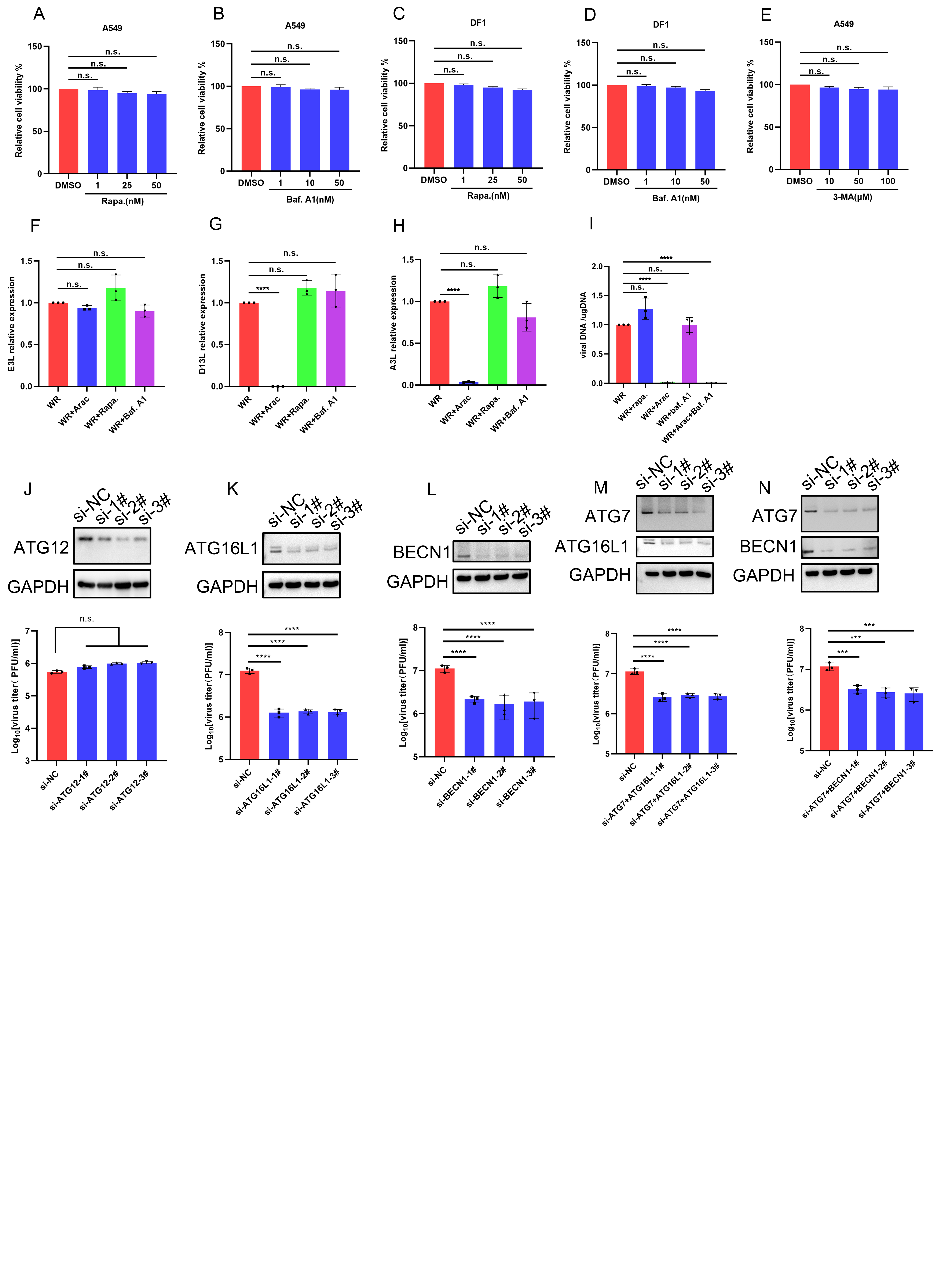

Supplement: S1 Fig — (A-B) A549 cells were treated with rapa. or baf. A1 at the concentrations mentioned above for 24 h and cell viability was measured by an MTT assay. (C-D) DF-1 cells were treated with rapa. or baf. A1 at the concentrations mentioned above for 24 h and cell viability was measured by an MTT assay. (E) A549 cells were treated with 3-MA at the concentrations mentioned above for 24 h and cell viability was measured by an MTT assay. (F-I) A549 cells were infected with VACV-WR in the presence of AraC (40 mg/mL), rapa. (100nM) or baf. A1 (0.1μM) for 2 h or 8 h and total RNA and DNA were harvested for mRNA and viral genomic DNA quantification using quantitative realtime-PCR with VACV-specific primers.(J-N) A549 cells were transfected with si-NC, si-ATG12 (J), si-ATG16L1(K), si-BECN1(L), si-ATG7 + si-ATG16L1 (M), and si-ATG7 + si-BECN1 (N) for 48 h and then were infected with the VACV-WR at an MOI of 3. After 24 hpi, virus titers were determined by a plaque assay. P-values were calculated using the one-way ANOVA; ns, not significant; ***P < 0.001; ****P < 0.0001. All experiments were performed in triplicate (N = 3 biological replicates). (TIF) [file ppat.1014137.s001.tif]

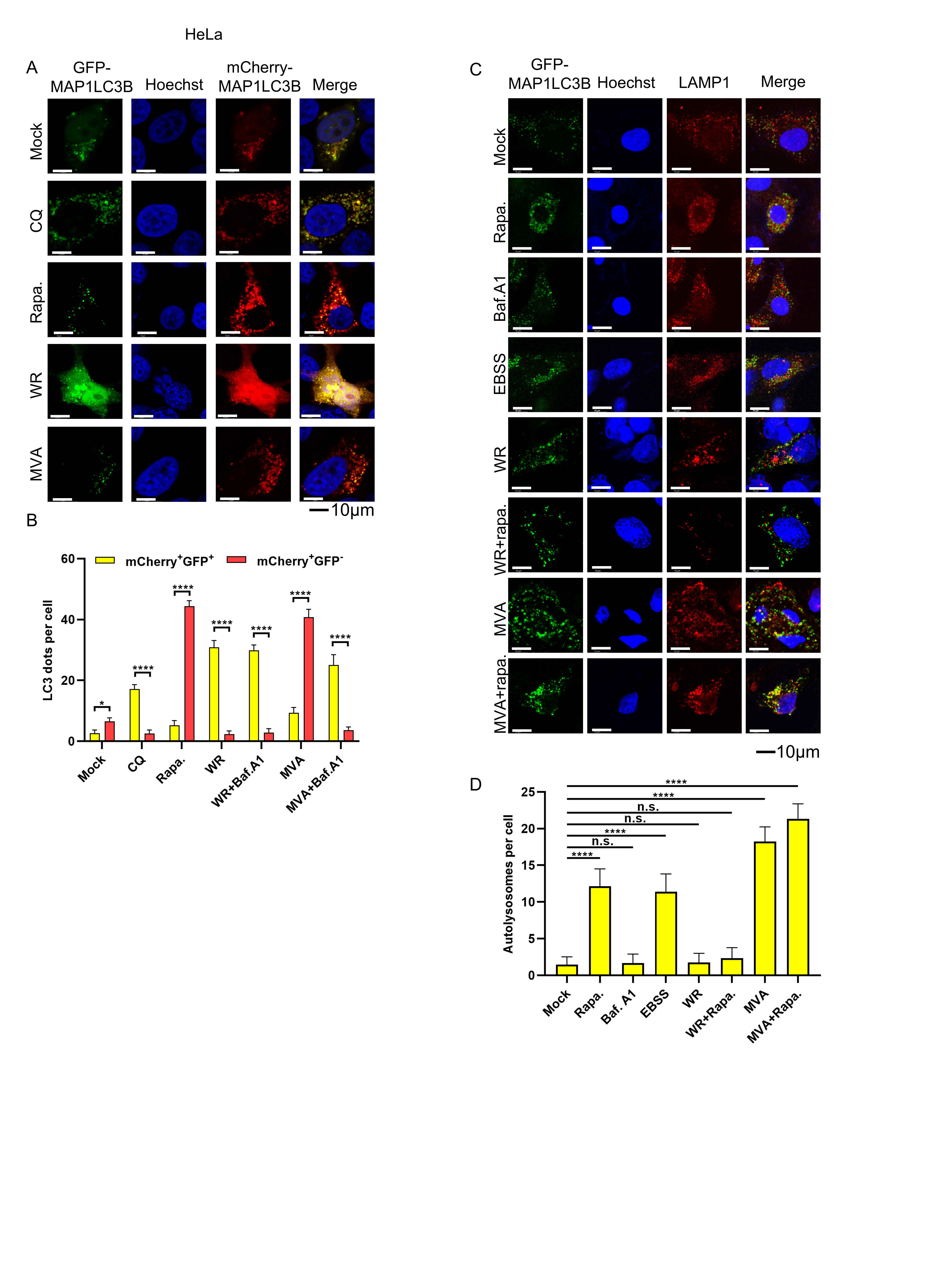

Supplement: S2 Fig — (A) HeLa cells were transfected with mCherry-GFP-MAP1LC3B for 24 h and then were mock infected or infected with WR, MVA at 3 PFU/cell or treated with CQ or rapa. for 12 h, and then cells were fixed, permeabilized, blocked, stained with Hoechst and analyzed with a fluorescent confocal microscope. Scale bar, 10 μm. (B) The graph shows the quantification of autophagosomes by taking the average number of dots in 15 cells. (C) A549 cells stably expressing MAP1LC3B on cover slips were infected with VACV-WR, MVA and at 3 PFU/cell or treated with baf. A1 (0.1μM) or rapa. (100nM). At 12 hpi, cells were then fixed, permeabilized, blocked, and stained with primary antibodies to LAMP1 and followed by fluorescent conjugated secondary antibodies. Hoechst was used to stain nucleus. Scale bars represent 10 μm. (D) Images were taken as described above and quantification of colocalization was analyzed by Image J software in 15 randomly selected cells. Data in S2A-S2D Fig are representative of three independent experiments (N = 3 biological replicates). (TIF) [file ppat.1014137.s002.tif]

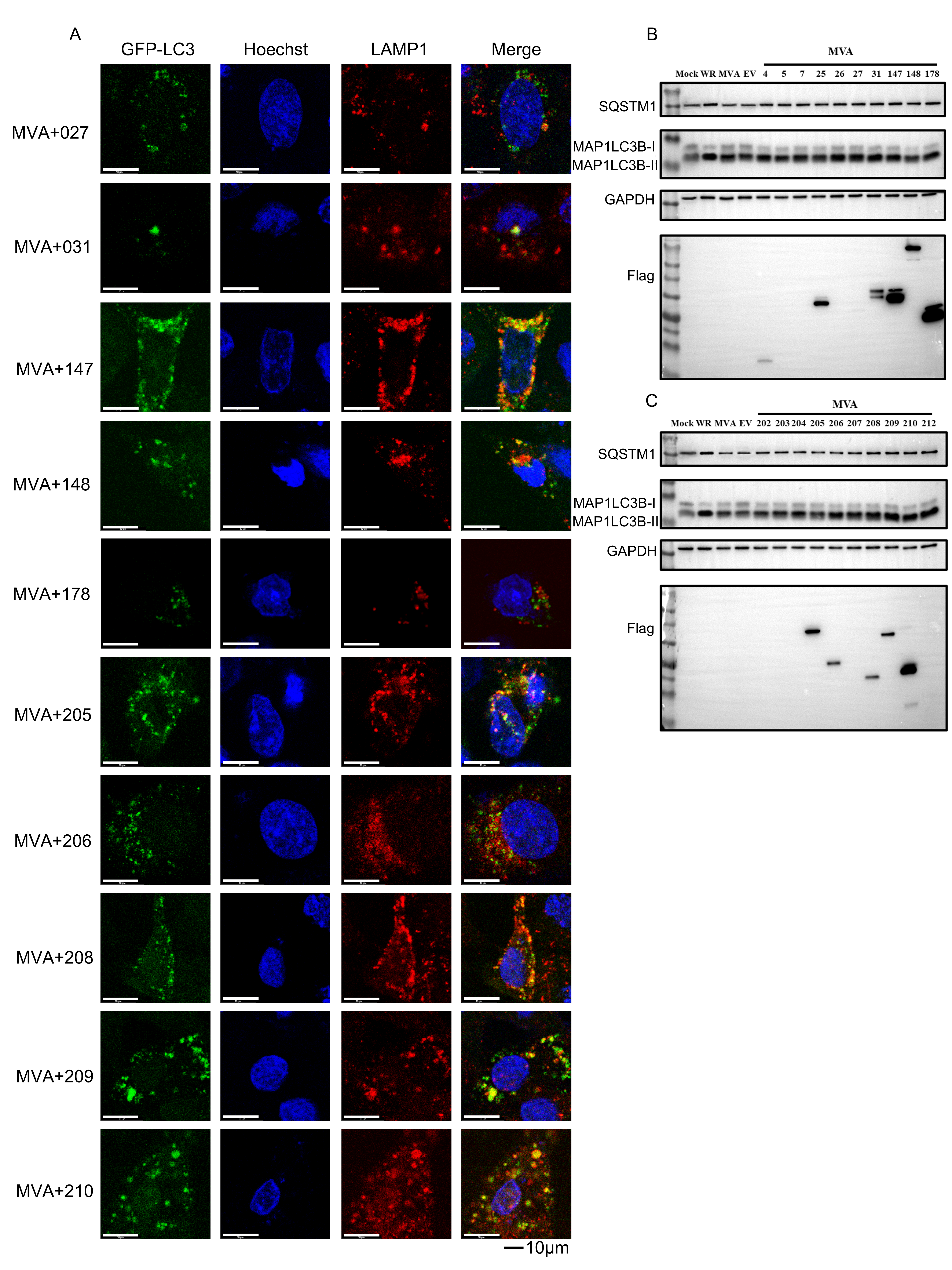

Supplement: S3 Fig — (A) Human A549 cells that stably express GFP-MAP1LC3B were transfected with plasmids of all candidate genes prior to MVA infection at 3 PFU/cell. At 12 hpi, cells were fixed, permeabilized, blocked, and stained with primary antibodies to LAMP1 and followed by fluorescent conjugated secondary antibodies. Hoechst was used to stain nucleus. Scale bar, 10 μm. (B-C) Human A549 cells were infected with 3PFU/cell WR or MVA, after 2 h, cells were transfected with plasmids of all candidate genes, and cell lysates were collected for Western blot analysis with anti-SQSTM1, anti-MAP1LC3B, anti-Flag, anti-GAPDH antibodies. Data in S3 A-S3C Fig are representative of three independent experiments (N = 3 biological replicates). (TIF) [file ppat.1014137.s003.tif]

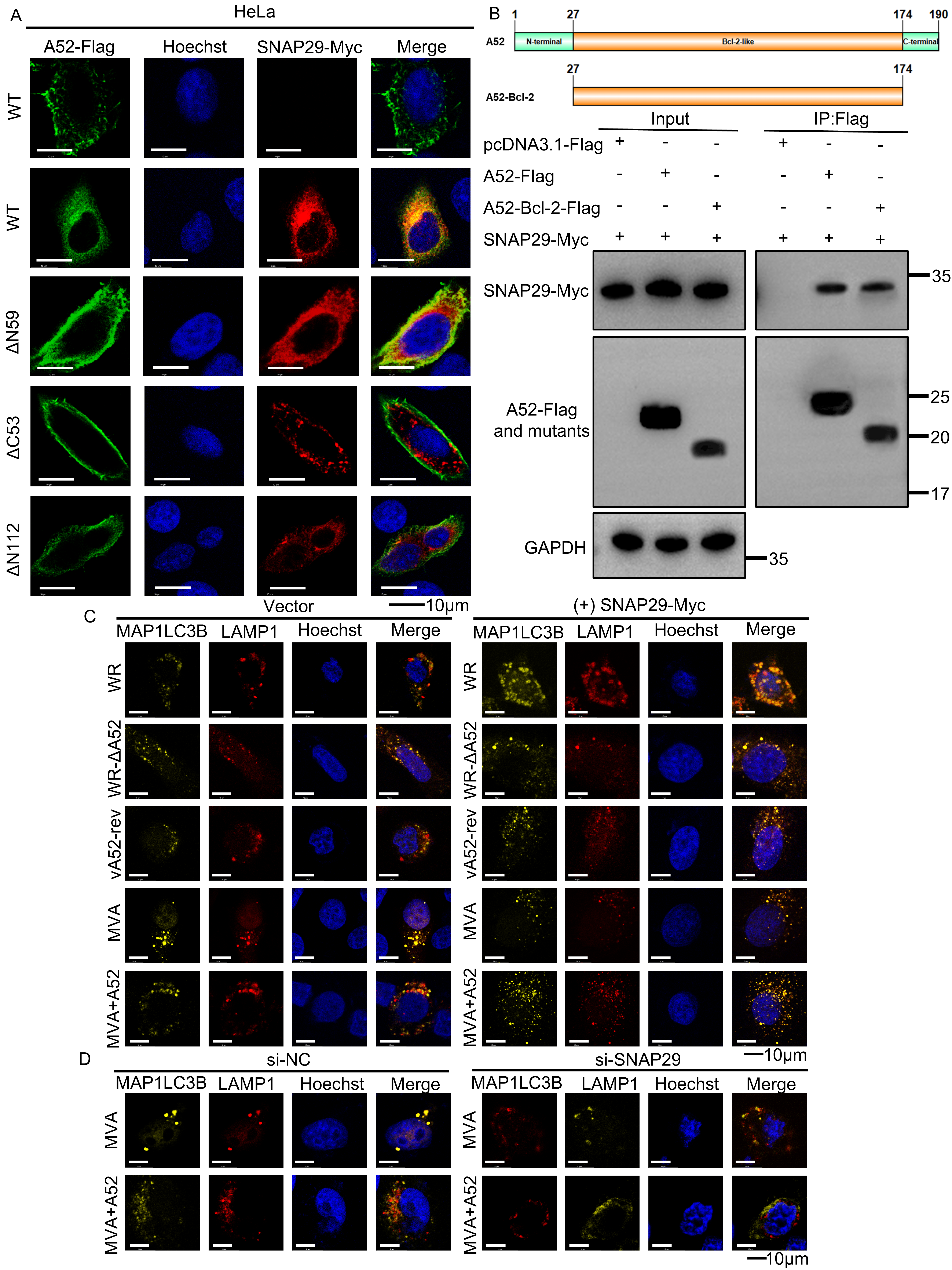

Supplement: S4 Fig — (A) HeLa cells grown on coverslips were transfected with vectors encoding Myc-tagged SNAP29 or its mutants, and a Flag-tagged A52 for 24 h. (The transfection doses of each plasmid were 0.5 μg). Cells were then fixed, permeabilized, blocked, and stained with primary antibodies to Myc or Flag and followed by fluorescent conjugated secondary antibodies. Hoechst was used to stain nucleus. Scale bars represent 10 μm. (B) A549 cells were co-transfected with vectors encoding Flag-tagged A52 or its mutants, and a Myc-tagged SNAP29 for 36 h. Cell lysates were pre-cleared with control magnetic beads or Flag-conjugated beads at 4˚C for 18 h followed by extensive washing. Proteins were eluted with SDS loading buffer and resolved by SDS-PAGE followed by Western blotting analysis using primary antibodies for Flag, Myc and GAPDH. (C) A549 cells were transfected with SNAP29-Myc at a concentration of 1.5μg/mL for 24h, and then the cells were infected with indicated viruses including WR, WR-ΔA52, vA52-rev, MVA, MVA + A52 at 3 PFU/cell for 12h. Cells were then fixed, permeabilized, blocked, and stained with primary antibodies to LAMP1 or MAP1LC3B and followed by fluorescent conjugated secondary antibodies. LAMP1 was used to stain lysosomes. MAP1LC3B was used to stain autophagosome. Hoechst was used to stain nucleus. Scale bars represent 10 μm. (D) A549 cells were transfected with siNC or siSNAP29 for 48 h and then infected in triplicates with MVA or MVA + A52 at 3 PFU/cell for 12h. Cells were then fixed, permeabilized, blocked, and stained with primary antibodies to LAMP1 or MAP1LC3B and followed by fluorescent conjugated secondary antibodies. LAMP1 was used to stain lysosomes. MAP1LC3B was used to stain autophagosome. Hoechst was used to stain nucleus. Scale bars represent 10 μm. Data in A-D are representative of three independent experiments (N = 3 biological replicates). (TIF) [file ppat.1014137.s004.tif]

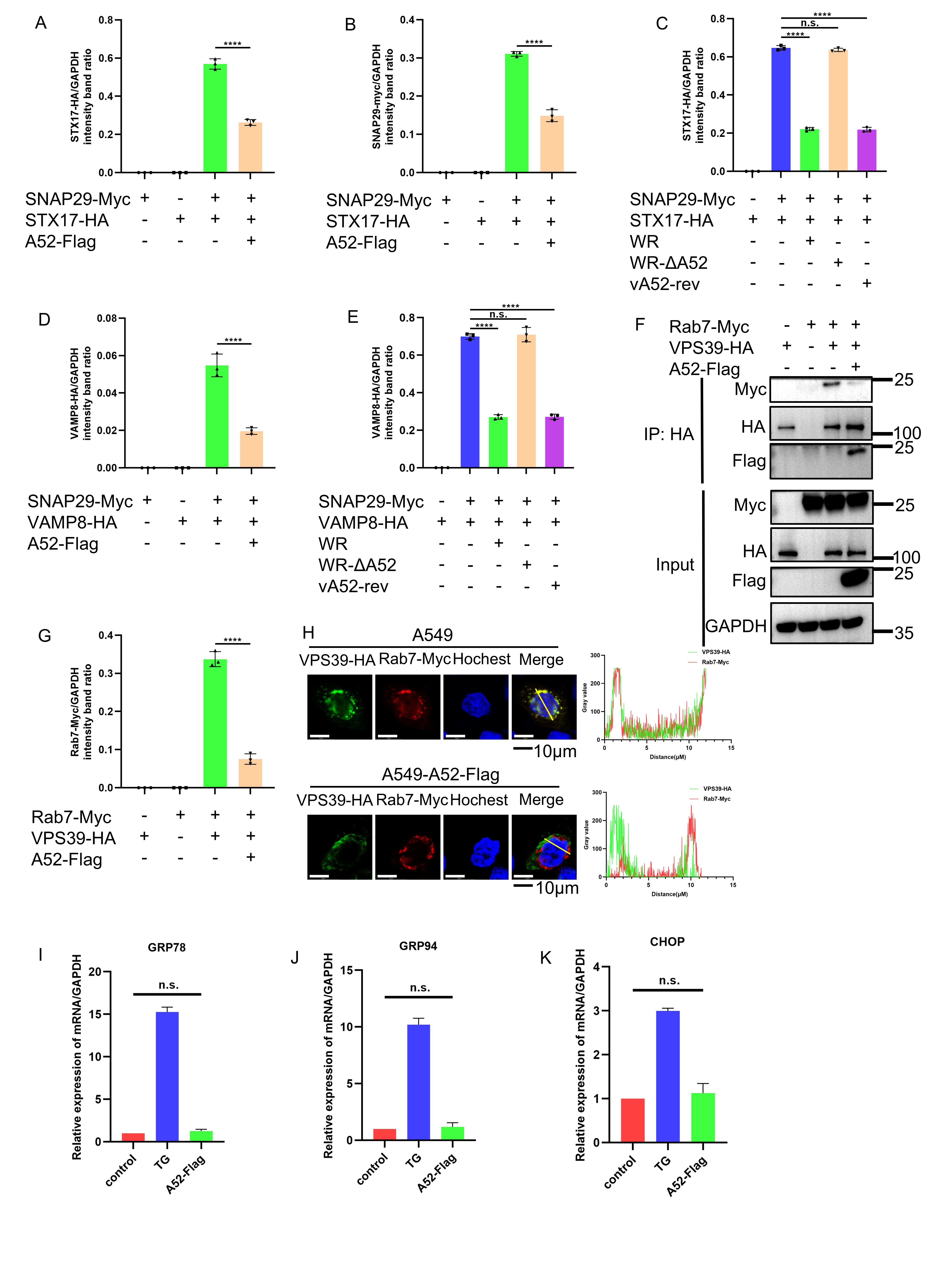

Supplement: S5 Fig — (A)The quantification of STX17-HA. (B) The quantification of SNAP29-Myc. (C) The quantification of STX17-HA. (D-E) The quantification of VAMP8-HA. (F) A549 cells were co-transfected with Rab7-Myc, VPS39-HA and Flag tagged A52 from VACV. Cell lysates were pre-cleared with control magnetic beads and then incubated with HA-conjugated beads at 4˚C for 18 h. Beads were extensively washed, and proteins were eluted with SDS loading buffer and resolved by SDS-PAGE and Western blotting analysis. (G) The quantification of Rab-Myc. (H) A549 cells on coverslips were co-transfected with Rab7-Myc, VPS39-HA and A52-Flag for 24 h. Cells were then fixed, stained with anti-Myc, anti-HA antibodies and Hoechst and images were taken with a fluorescent confocal microscope. The right panels show the fluorescence intensity profile of VPS39-HA (green) and Myc-tagged Rab7 (red) measured along the line drawn by Image J. Scale bars represent 10 μm. (I-K) A549 cells were transfected with A52-Flag. Cells treated with TG (5 μM) for 4 h served as positive controls. Total RNA was harvested at 36 hours post-transfection, and the relative expression of GRP78 (I), GRP94 (J), CHOP (K) was detected by qPCR. Data in S5A-S5K Fig are representative of three independent experiments (N = 3 biological replicates). (TIF) [file ppat.1014137.s005.tif]
