# Supplementary material for: Poxvirus A52 protein subverts autophagy flux by blocking autophagosome–lysosome fusion to promote viral replication
Source: PLoS Pathog. 2026 Apr 13;22(4):e1014137. doi: 10.1371/journal.ppat.1014137 (PMC13075716; doi:10.1371/journal.ppat.1014137)
Supplement: S1 Table — (DOCX) [file ppat.1014137.s006.docx]

**Table S1.** The main targets of Baf.A1.

| Gene | Log_2_FC | Functions | Reference |
| --- | --- | --- | --- |
| A3L | -1.97595 | Participate in the assembly and maturation process of viruses | ^1,2^ |
| A10L | -1.75117 | Participate in the formation of the viral membrane and the maturation of the viral particles | ^1,2^ |
| A12L | -1.02428 | Participate in the formation and stability of virus particles | ^3^ |
| A14L | -2.07525 | Participate in the formation of the viral membrane and the maturation of the viral particles | ^4^ |
| A17L | -1.79275 | Participate in the formation of the viral membrane and the maturation of the viral particles | ^5^ |
| G6R | -1.8273 | Participate in the replication of the virus and the regulation of its virulence | ^6^ |
| G7L | -2.34258 | Participate in the formation of the viral membrane and the assembly of viral particles. | ^7^ |
| H3L | -1.39292 | Participate in the formation and stability of virus particles | ^8^ |
| I7L | -2.08597 | Cutting the substrate containing the AG*X conserved motif. Participate in the processing and maturation process of viral proteins | ^9^ |

**References:**

[1] Chung C, Chen C, Ho M, Huang C, Liao C, Chang W, Vaccinia virus proteome: identification of proteins in vaccinia virus intracellular mature virion particles, J VIROL 2006; 80: 2127-40;PMID:16474121;10.1128/JVI.80.5.2127-2140.2006

[2] Pedersen K, Snijder EJ, Schleich S, Roos N, Griffiths G, Locker JK, Characterization of vaccinia virus intracellular cores: implications for viral uncoating and core structure, J VIROL 2000; 74: 3525-36;PMID:10729126;10.1128/jvi.74.8.3525-3536.2000

[3] Yang SJ, Hruby DE, Vaccinia virus A12L protein and its AG/A proteolysis play an important role in viral morphogenic transition, VIROL J 2007; 4: 73;PMID:17625005;10.1186/1743-422X-4-73

[4] Rodriguez JR, Risco C, Carrascosa JL, Esteban M, Rodriguez D, Characterization of early stages in vaccinia virus membrane biogenesis: implications of the 21-kilodalton protein and a newly identified 15-kilodalton envelope protein, J VIROL 1997; 71: 1821-33;PMID:9032312;10.1128/JVI.71.3.1821-1833.1997

[5] Rodriguez D, Esteban M, Rodriguez JR, Vaccinia virus A17L gene product is essential for an early step in virion morphogenesis, J VIROL 1995; 69: 4640-8;PMID:7609028;10.1128/JVI.69.8.4640-4648.1995

[6] Coffman KA, Burke GR, Genomic analysis reveals an exogenous viral symbiont with dual functionality in parasitoid wasps and their hosts, PLOS PATHOG 2020; 16: e1009069;PMID:33253317;10.1371/journal.ppat.1009069

[7] Szajner P, Jaffe H, Weisberg AS, Moss B, Vaccinia virus G7L protein Interacts with the A30L protein and is required for association of viral membranes with dense viroplasm to form immature virions, J VIROL 2003; 77: 3418-29;PMID:12610117;10.1128/jvi.77.6.3418-3429.2003

[8] Da Fonseca FG, Wolffe EJ, Weisberg A, Moss B, Effects of deletion or stringent repression of the H3L envelope gene on vaccinia virus replication, J VIROL 2000; 74: 7518-28;PMID:10906205;10.1128/jvi.74.16.7518-7528.2000

[9] Moerdyk MJ, Byrd CM, Hruby DE, Analysis of vaccinia virus temperature-sensitive I7L mutants reveals two potential functional domains, VIROL J 2006; 3: 64;PMID:16945137;10.1186/1743-422X-3-64
