# Supplementary material for: Poxvirus A52 protein subverts autophagy flux by blocking autophagosome–lysosome fusion to promote viral replication
Source: PLoS Pathog. 2026 Apr 13;22(4):e1014137. doi: 10.1371/journal.ppat.1014137 (PMC13075716; doi:10.1371/journal.ppat.1014137)

Original Images for Blots and IF

Poxvirus A52 Protein Subverts Autophagy Flux by Blocking Autophagosome–Lysosome Fusion to Promote Viral Replication

Kang Niu, Yongxiang Fang, Yining Deng, Ziyue Wang, Shijie Xie, Junda Zhu, Baifen Song, Wenxue Wu, Zhizhong Jing, Chen Peng

Figure 1K

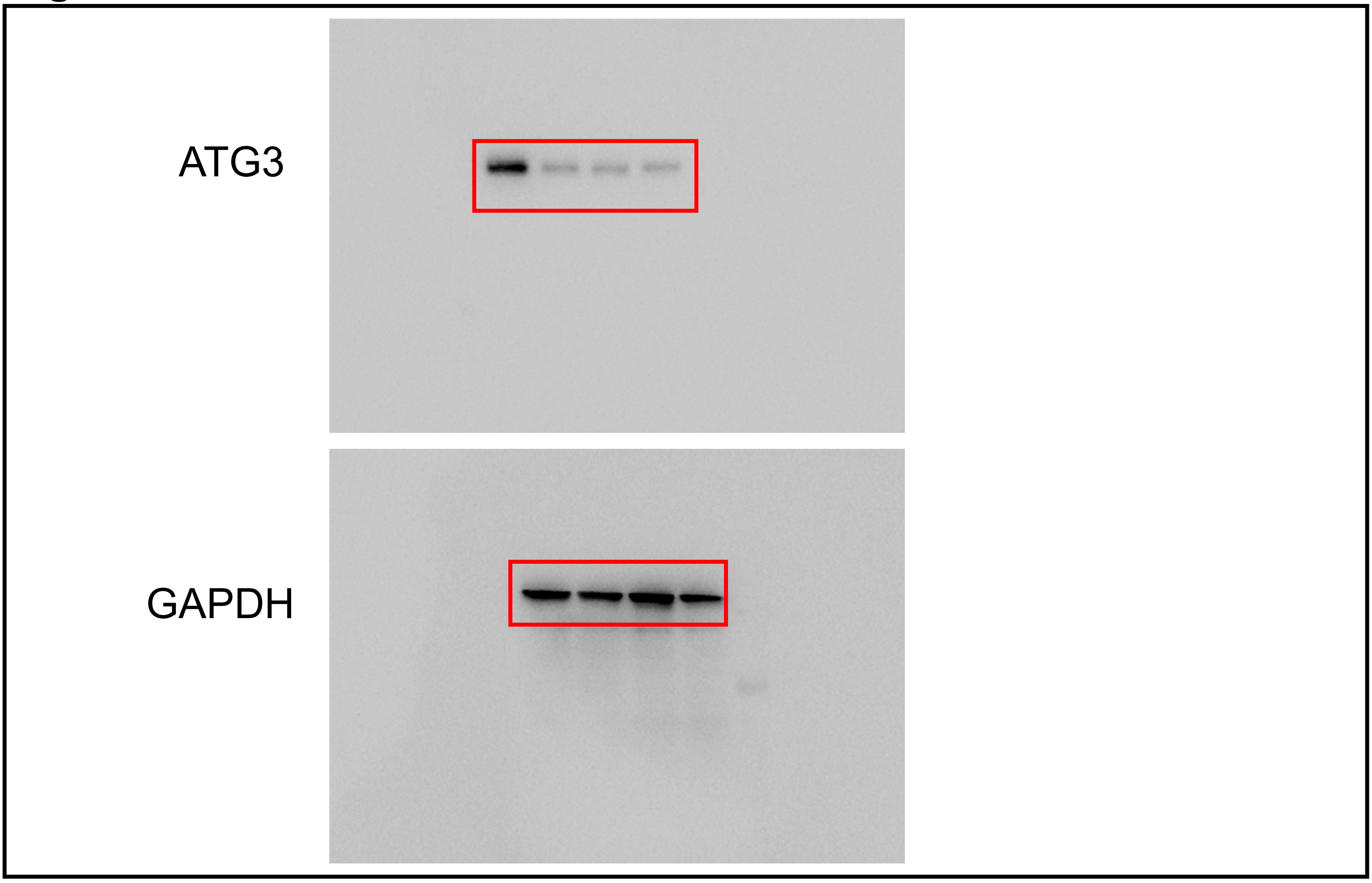

Figure 1L

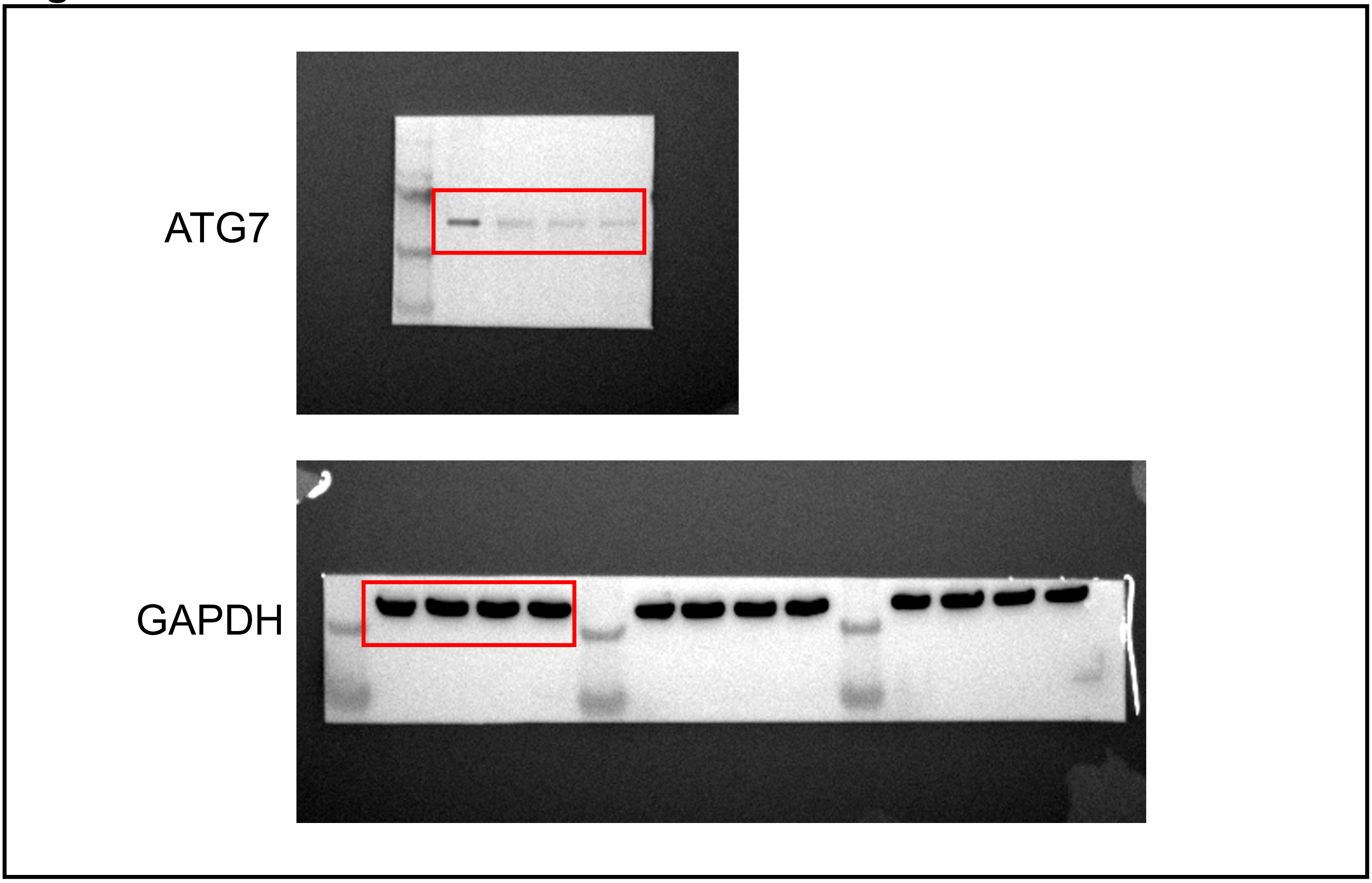

Figure 1M

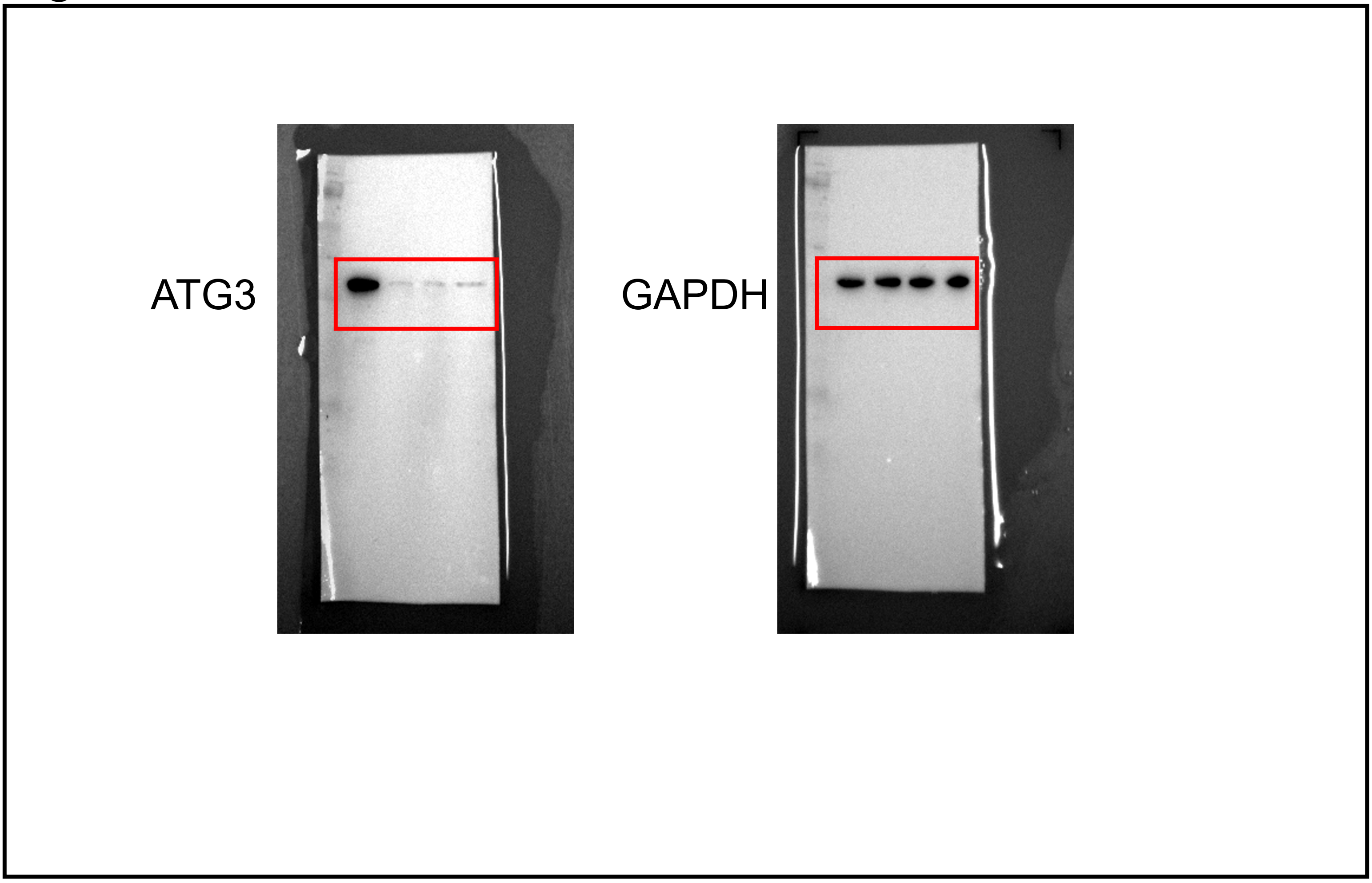

Figure 1N

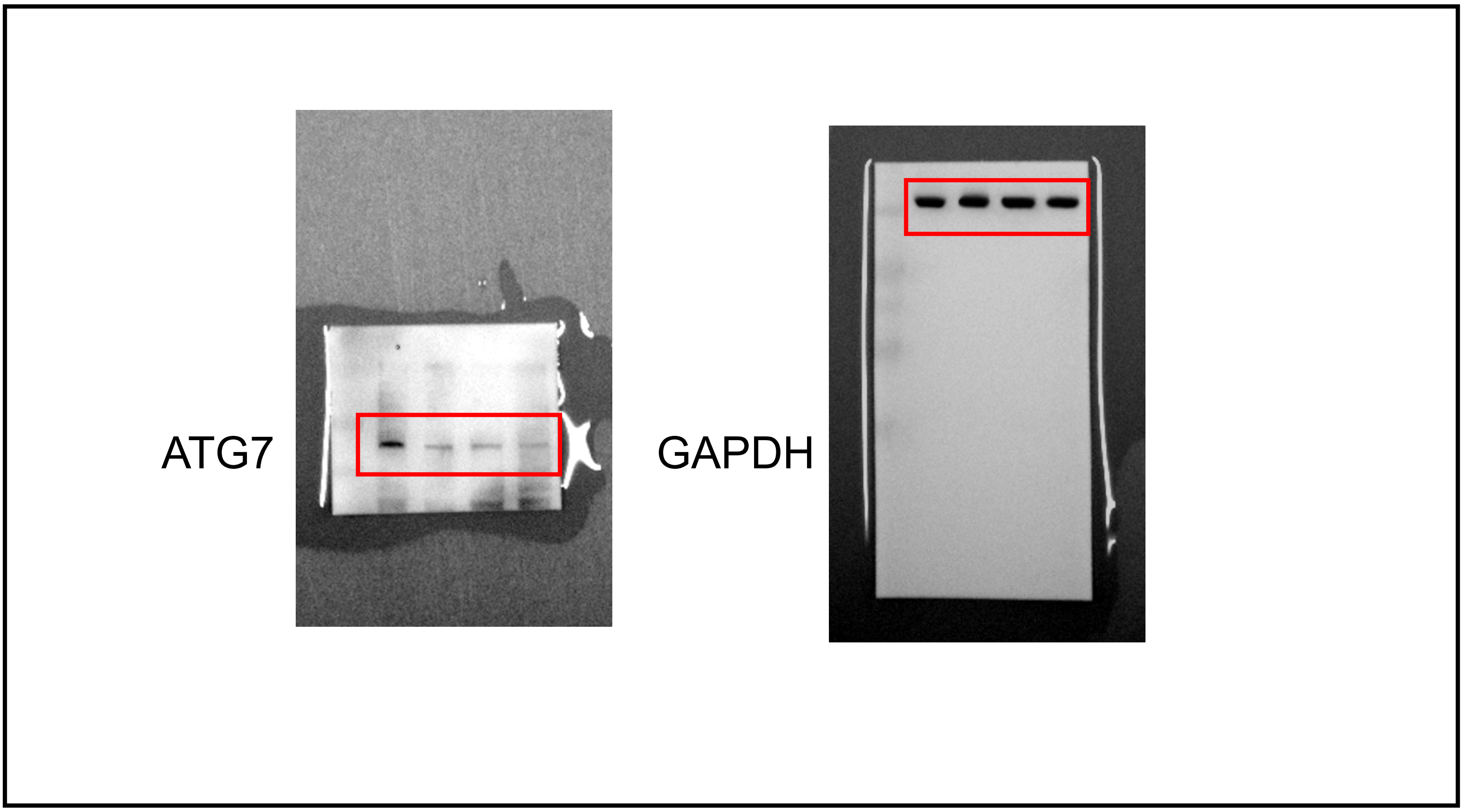

Figure 1O

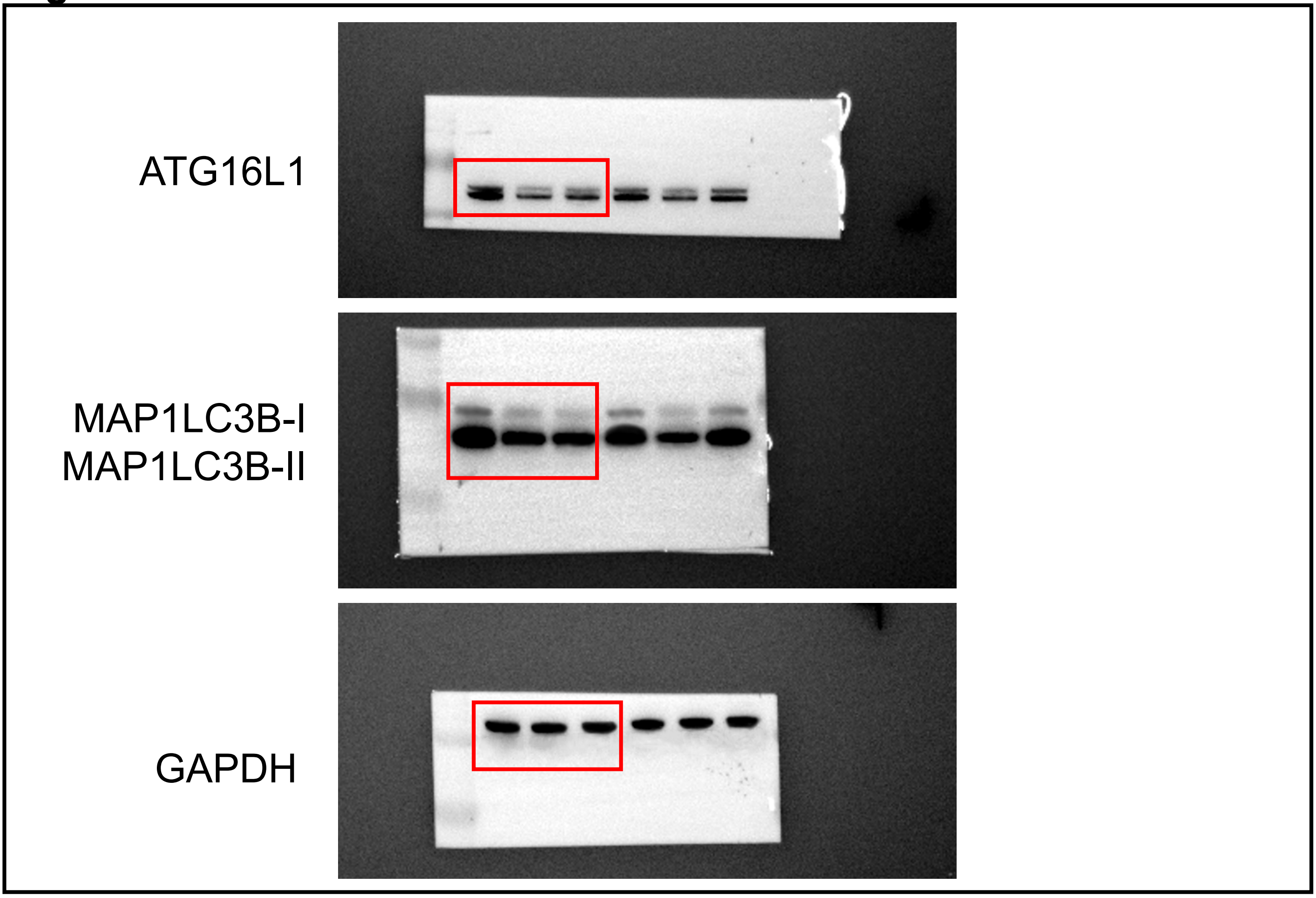

Figure 1P

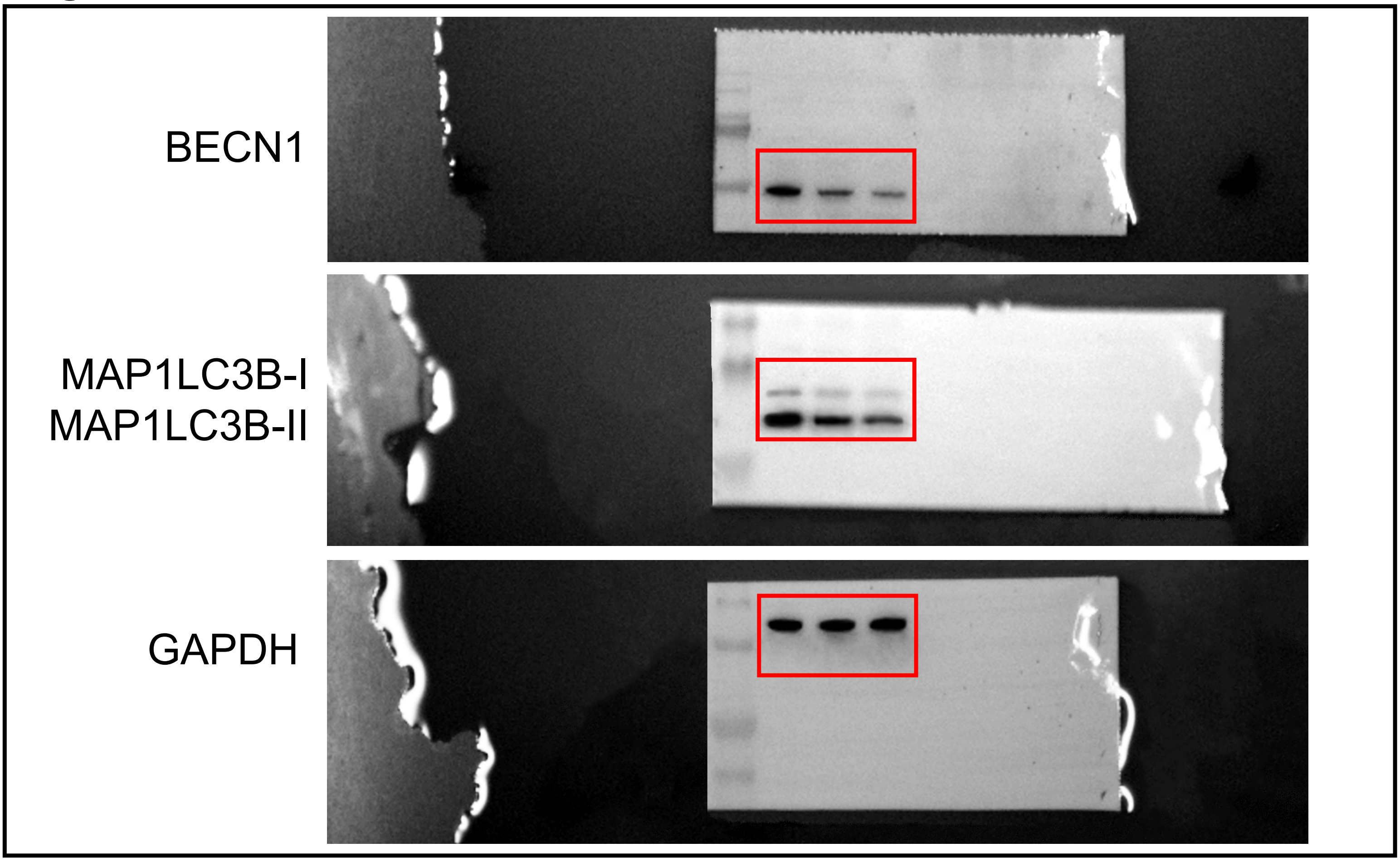

Figure 1Q

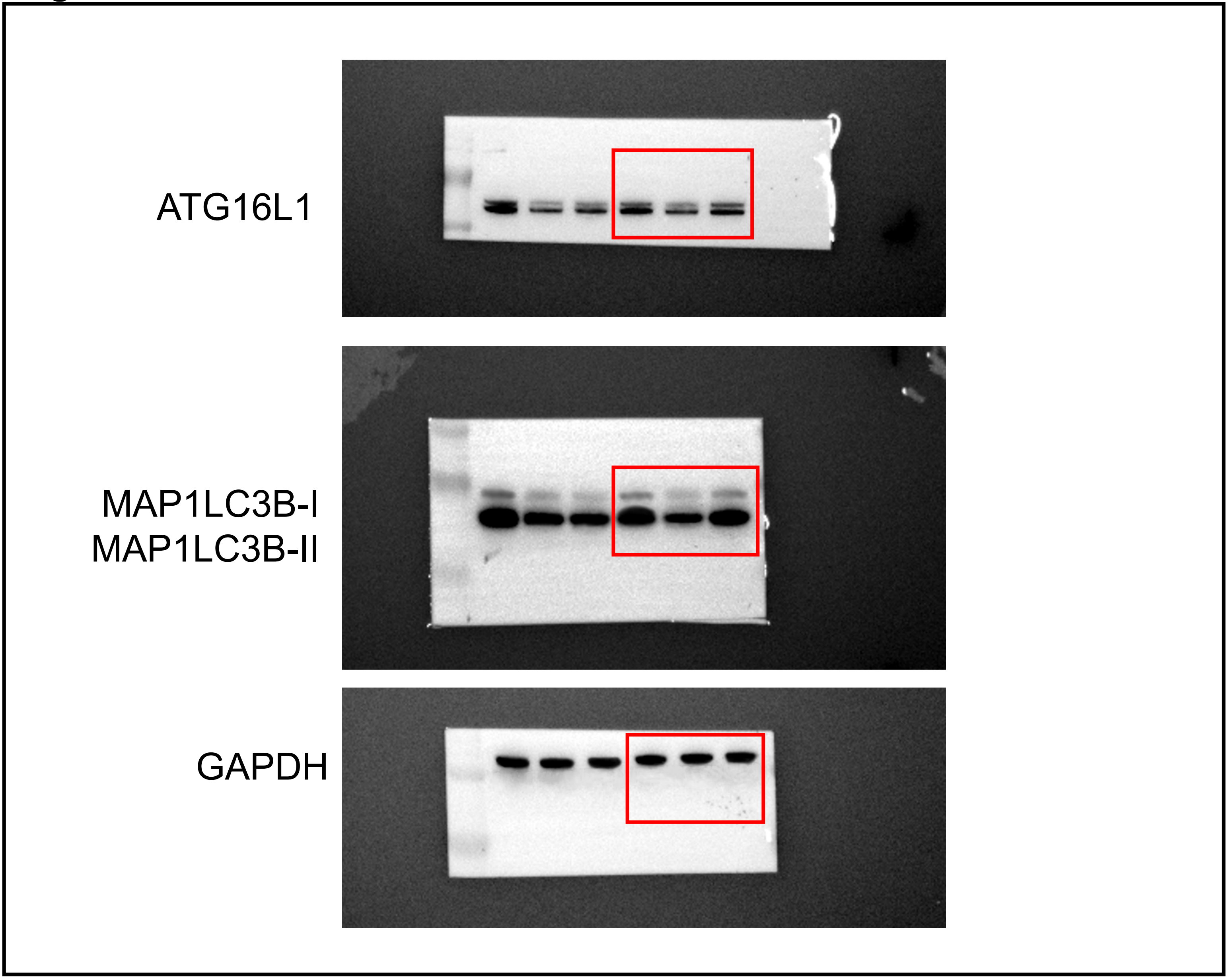

Figure 2A

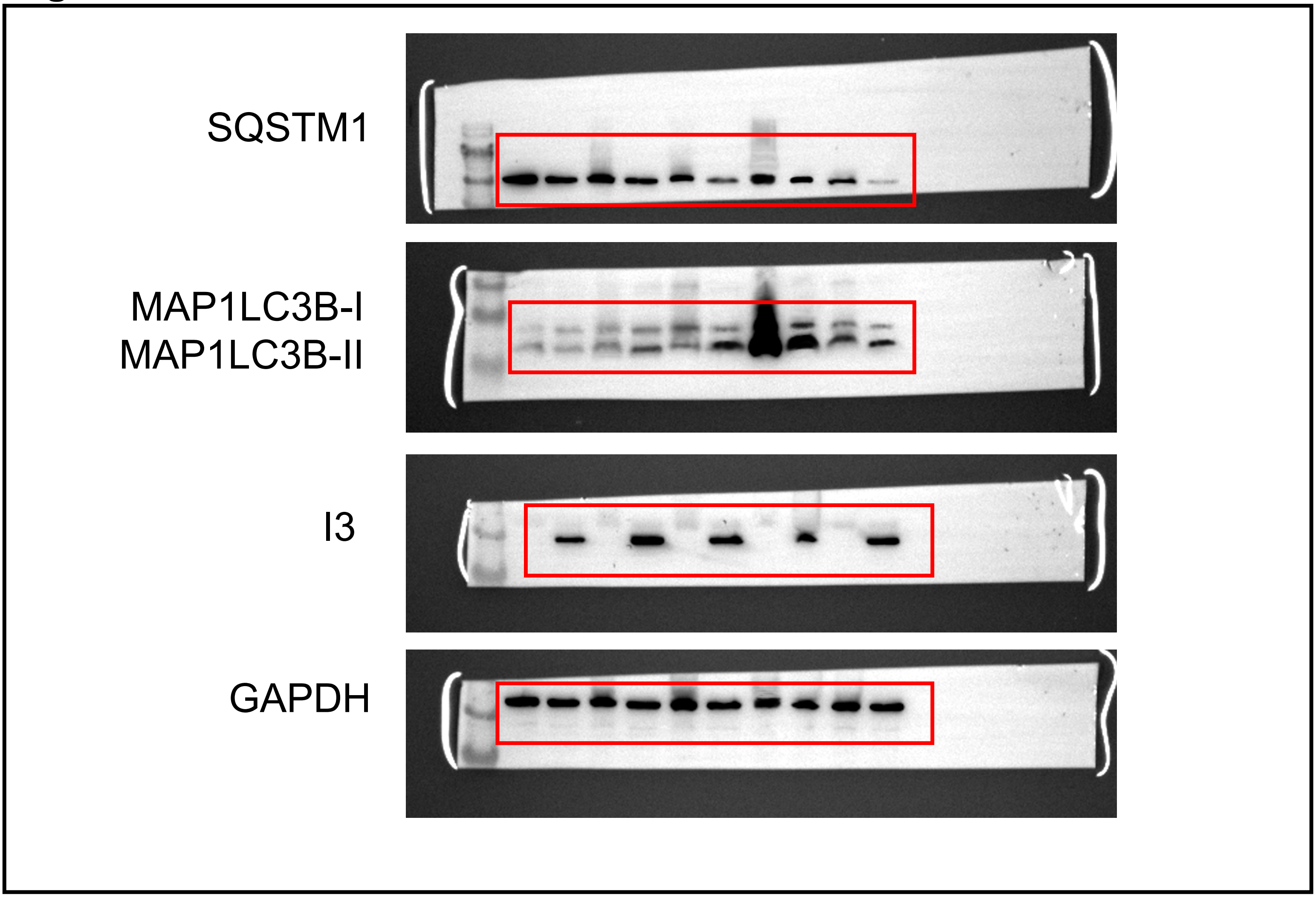

Figure 2B

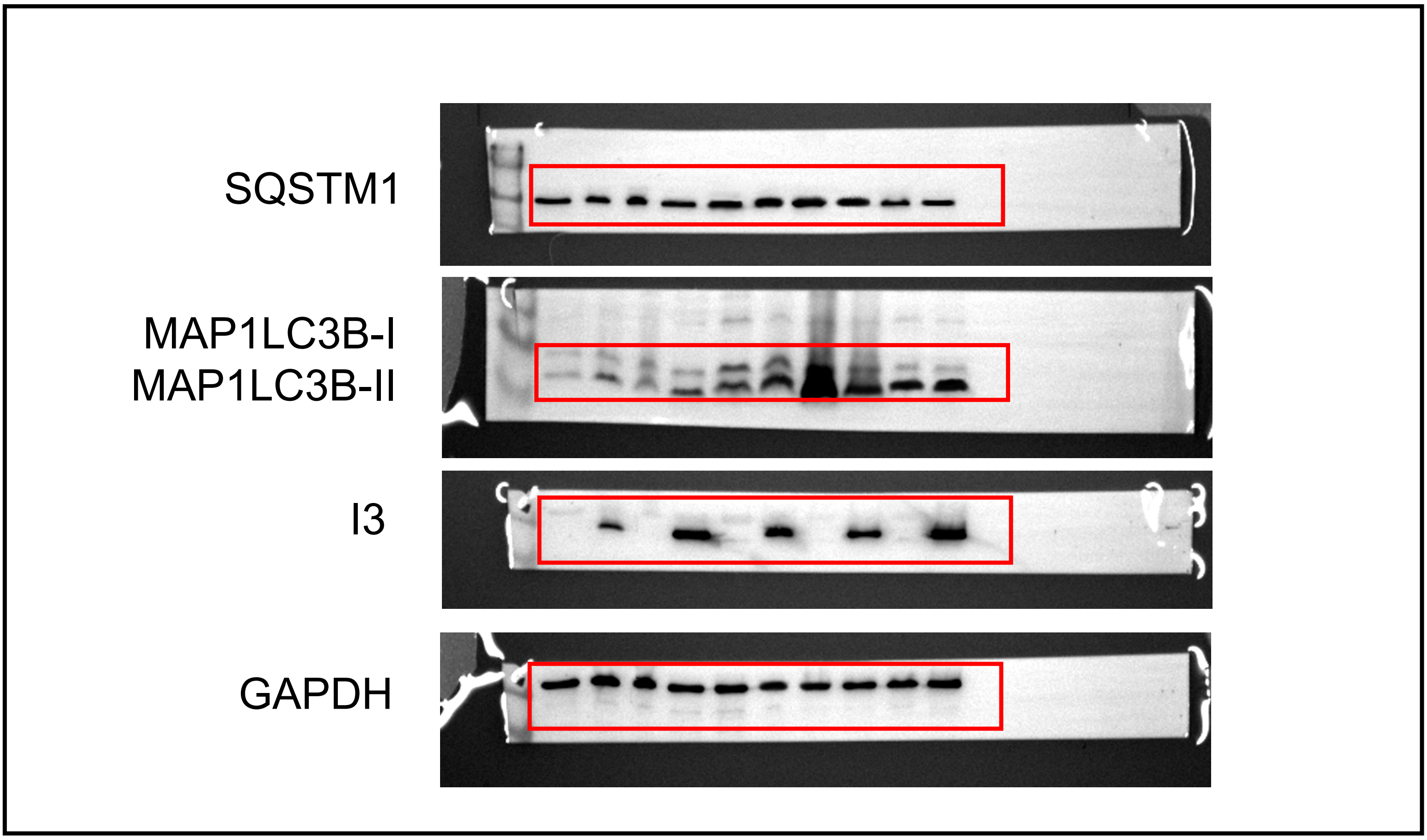

Figure 4A

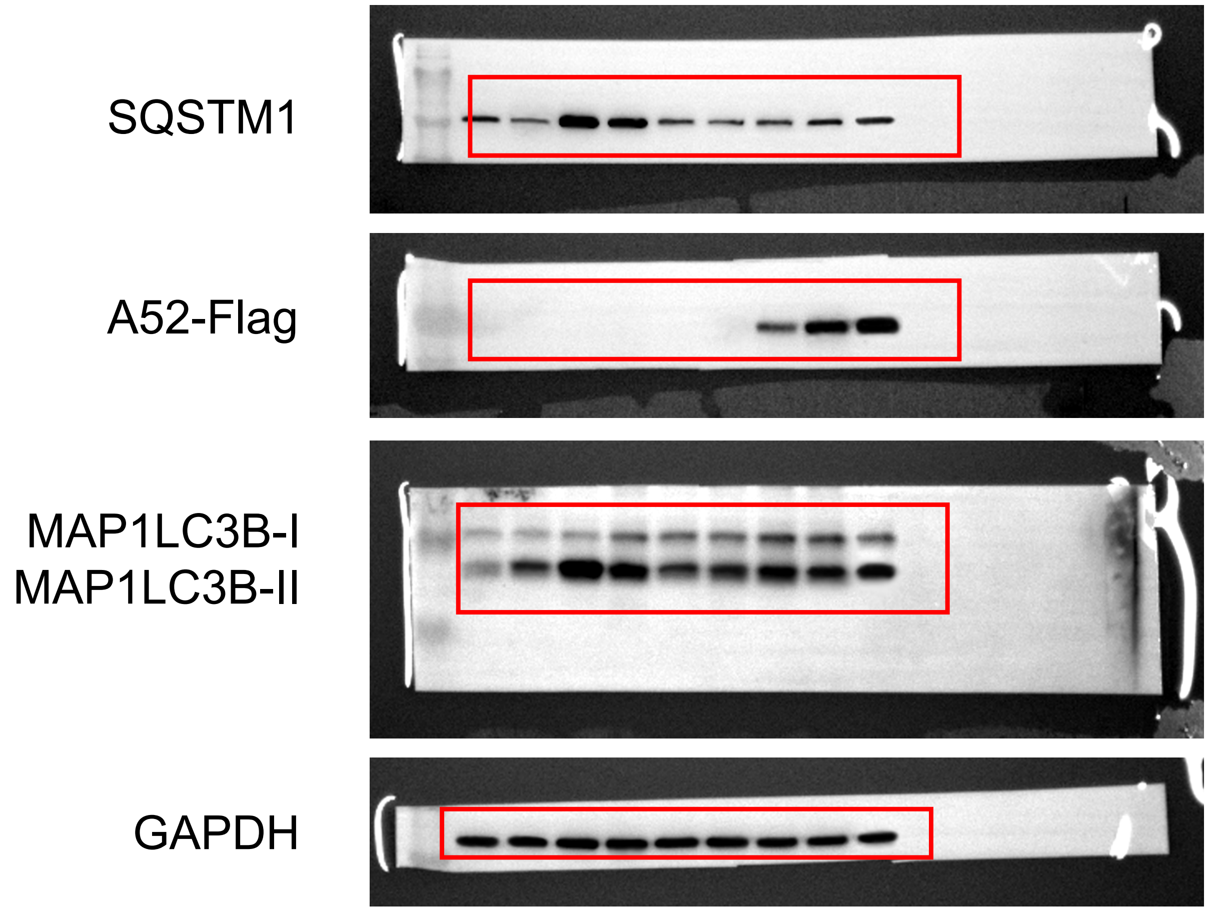

Figure 4B

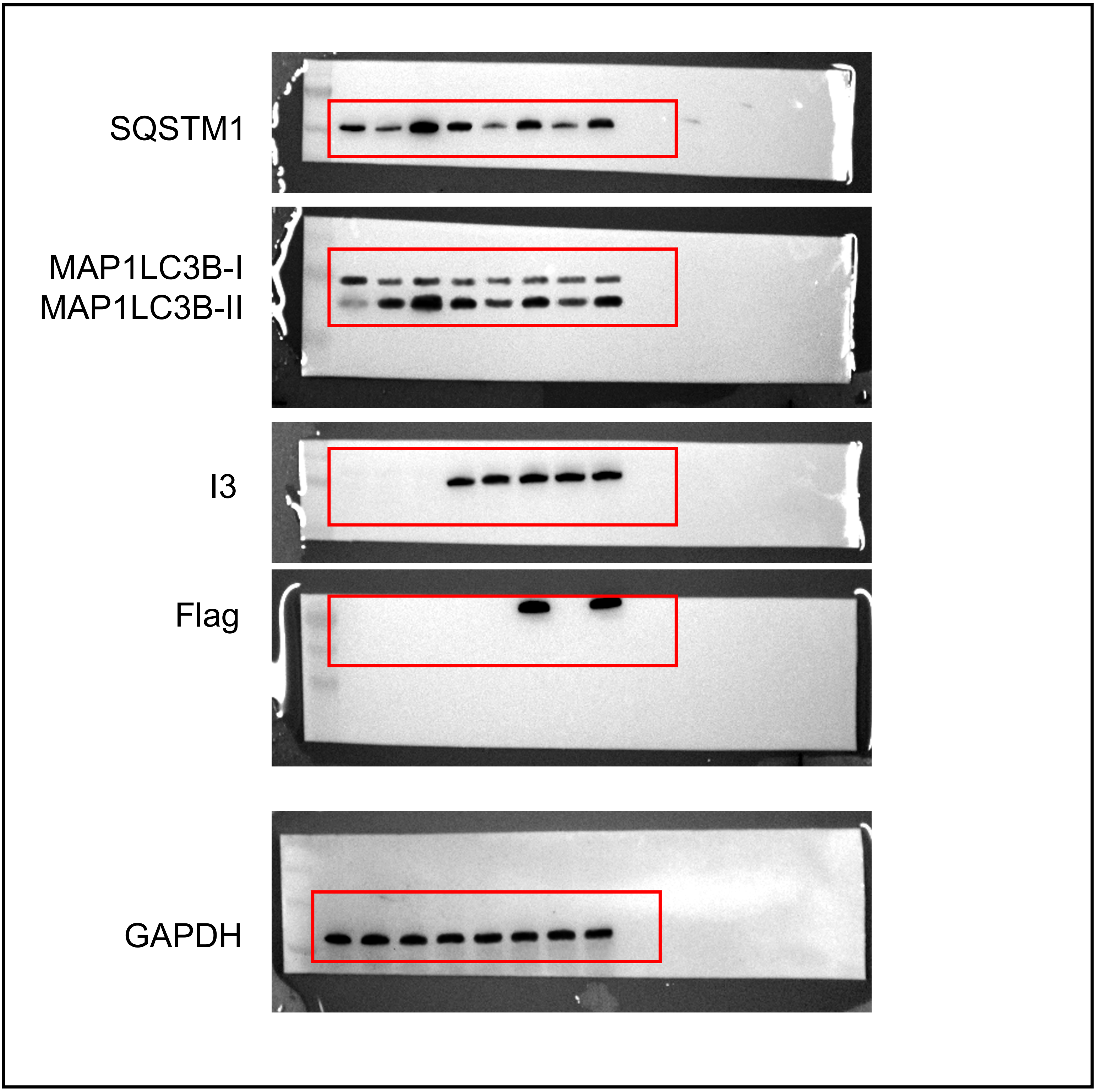

Figures 5A and 5B

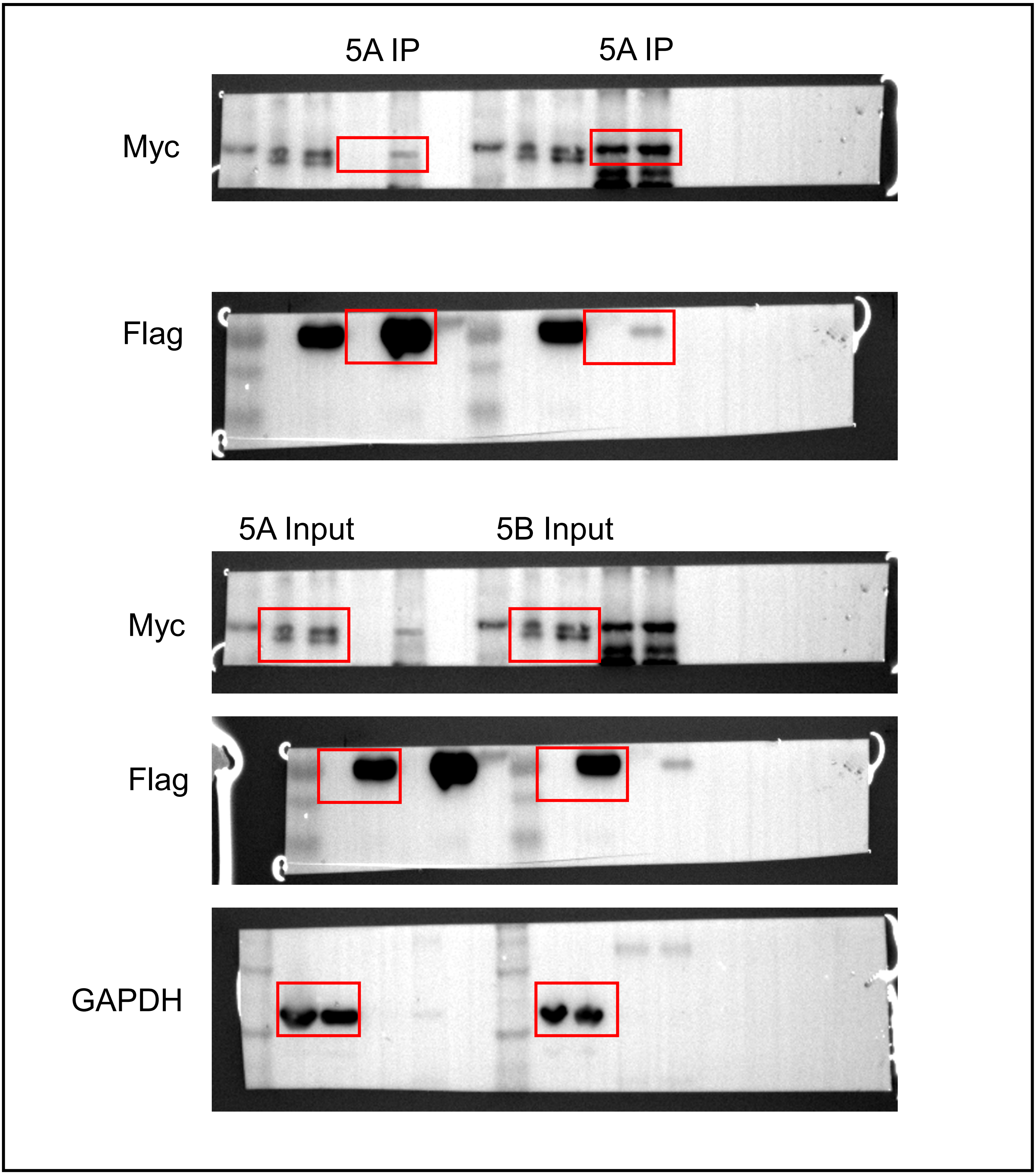

Figure 5C

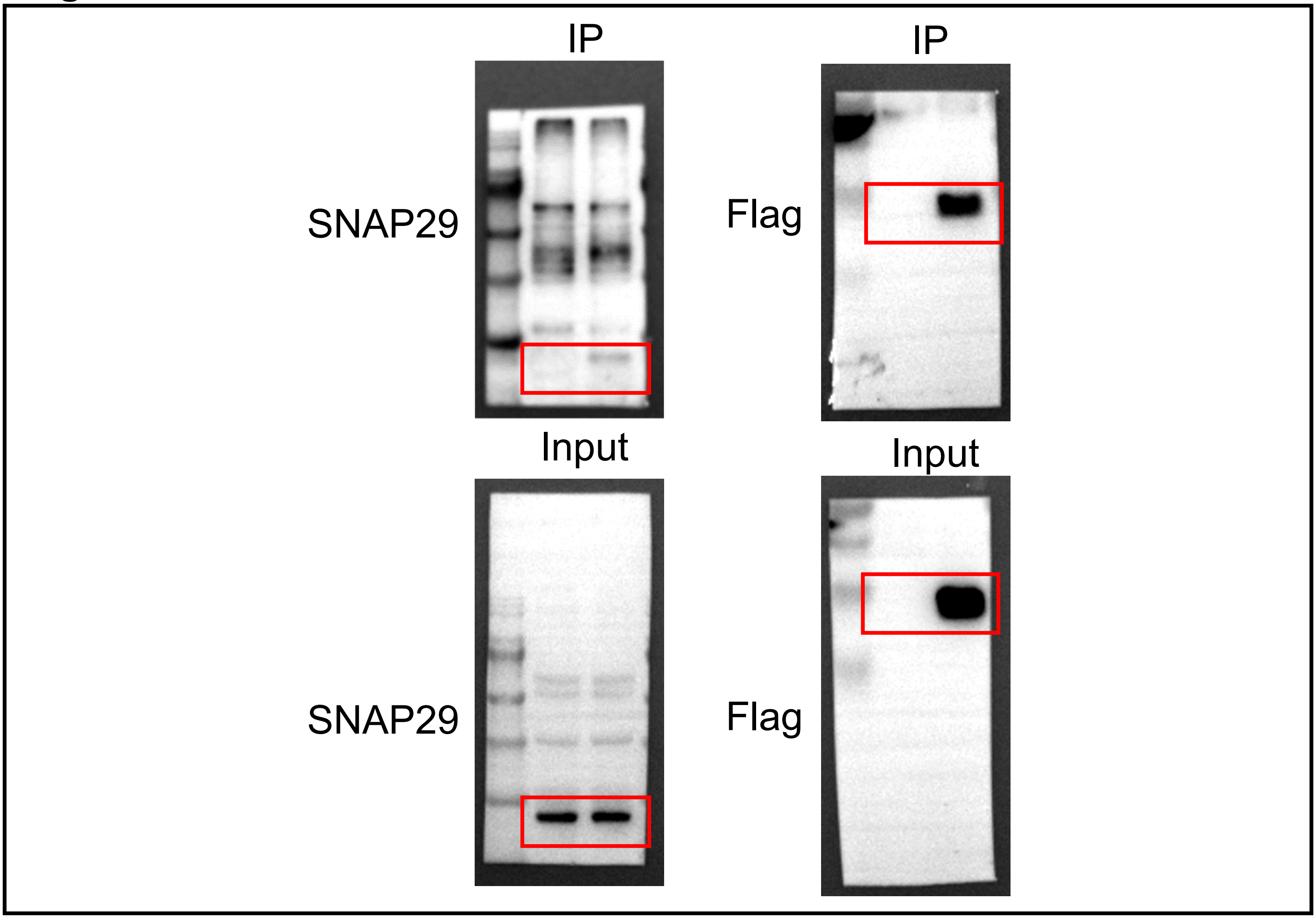

Figure 5F

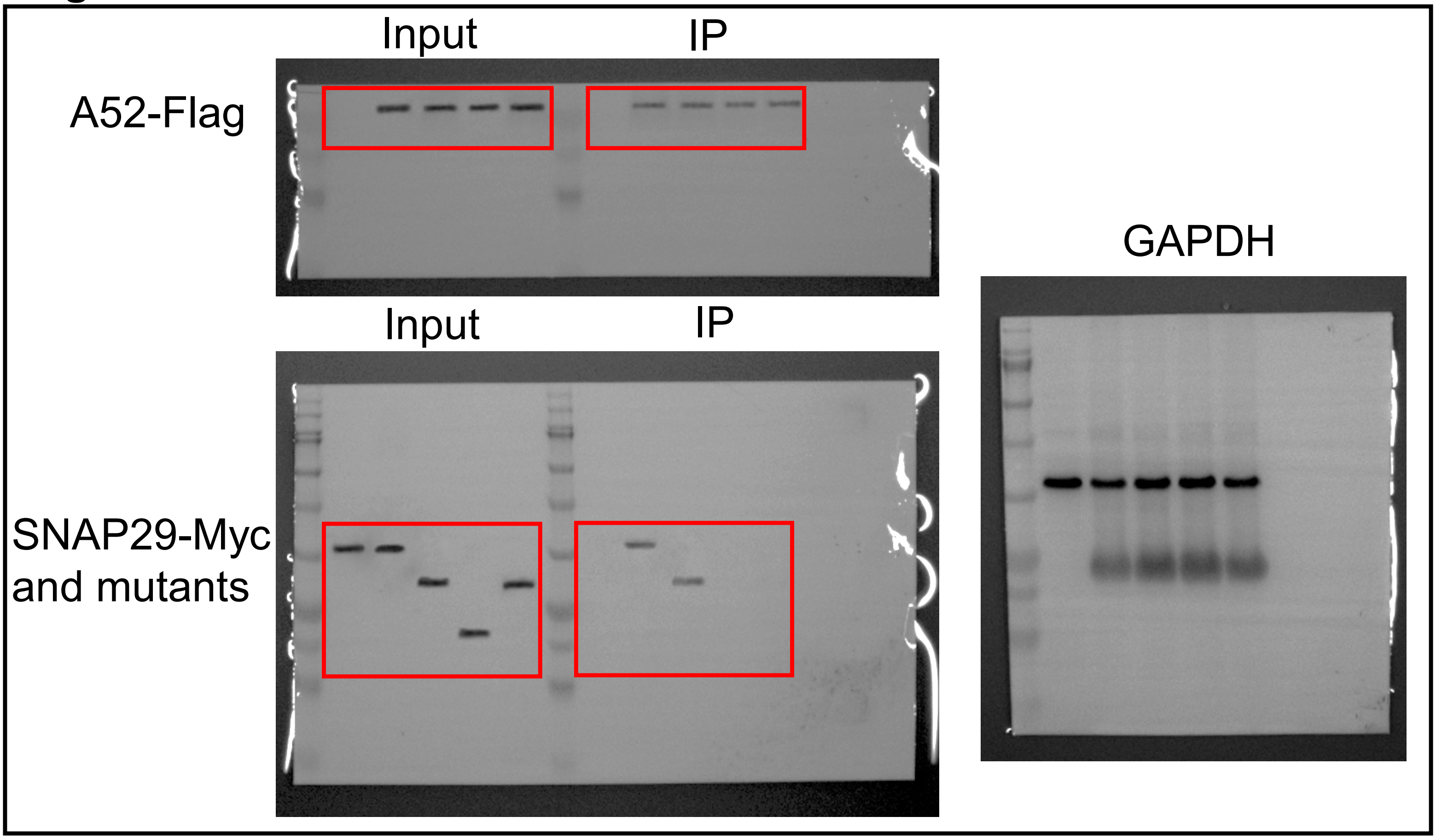

Figure 6A

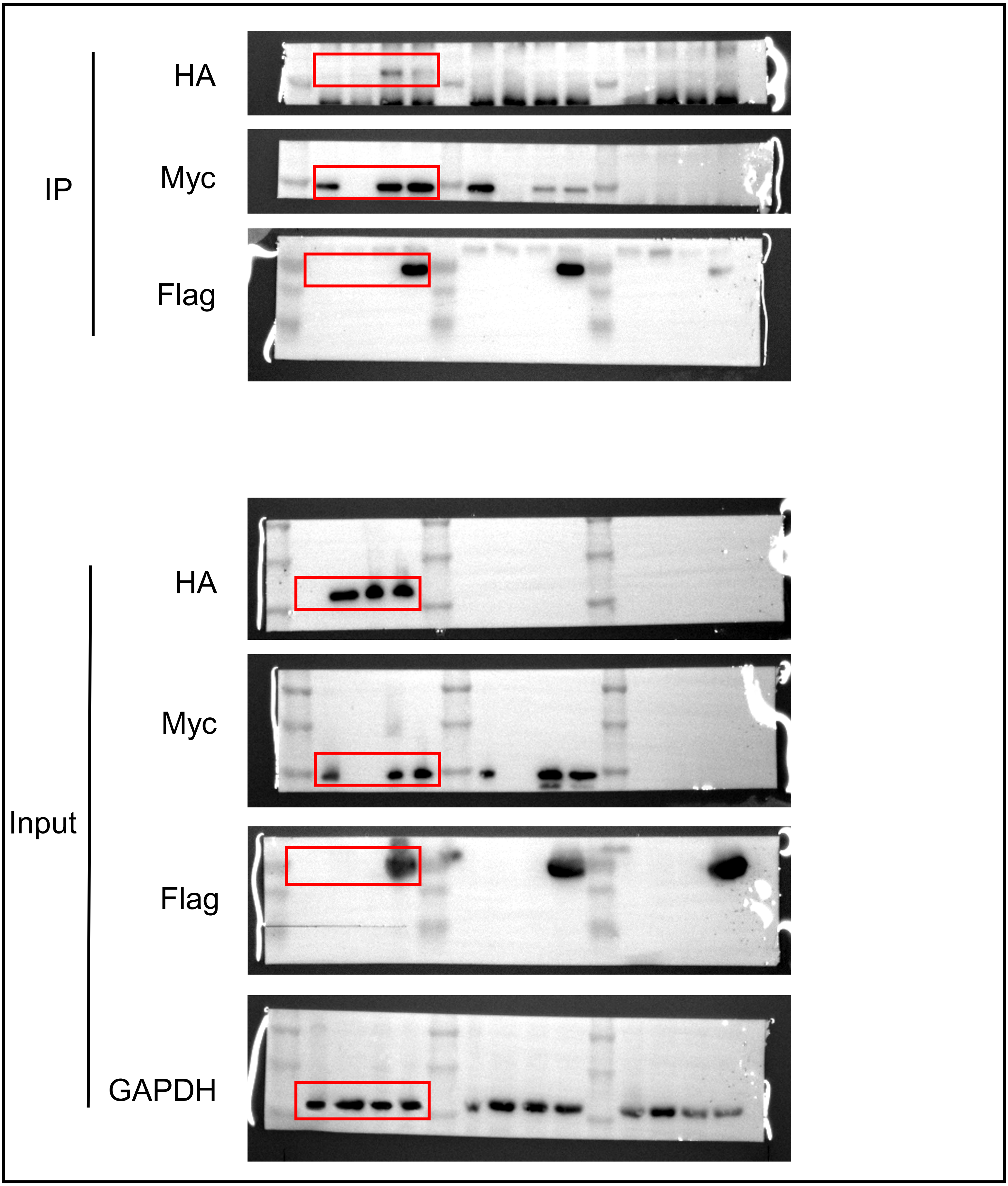

Figure 6B

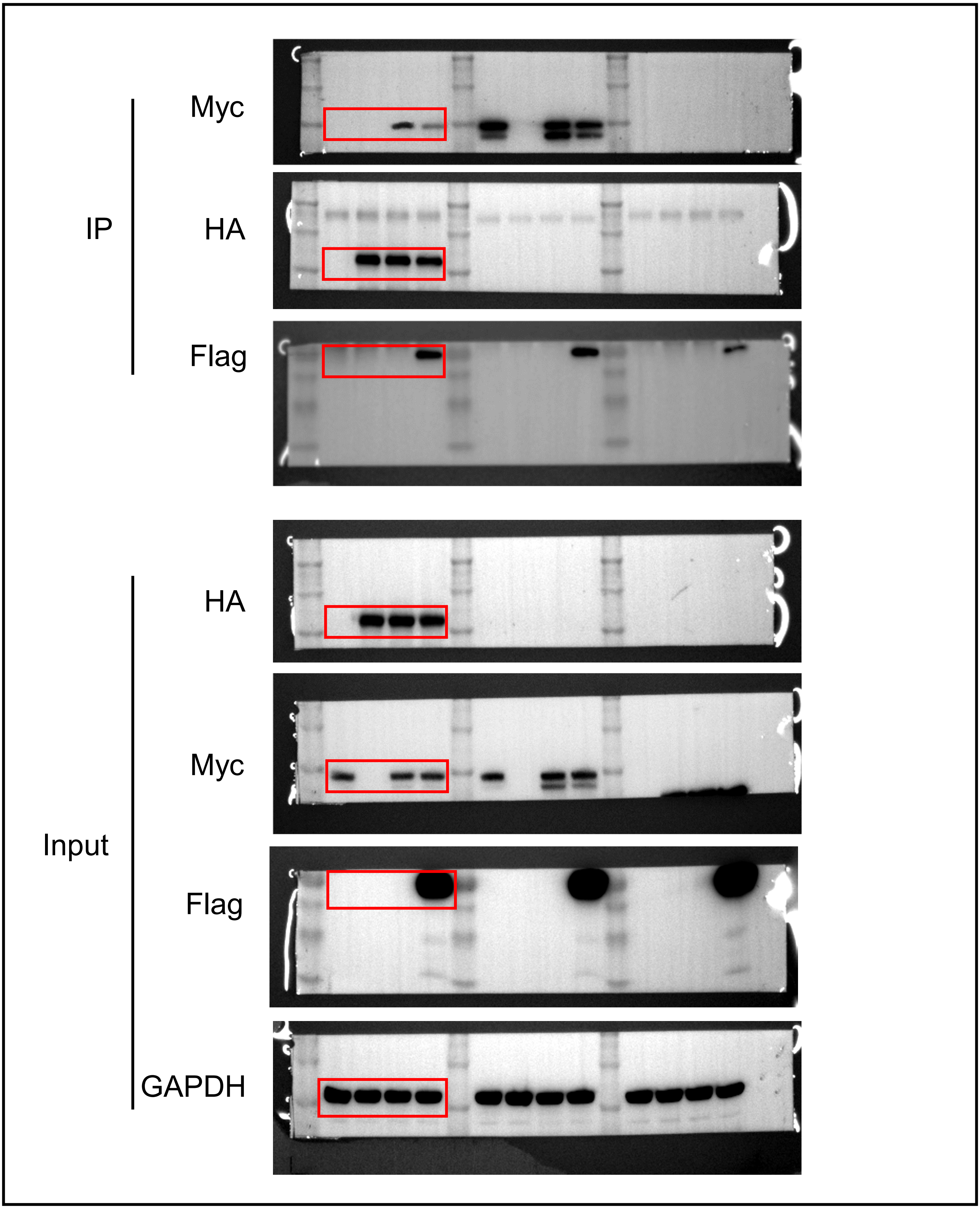

Figure 6D

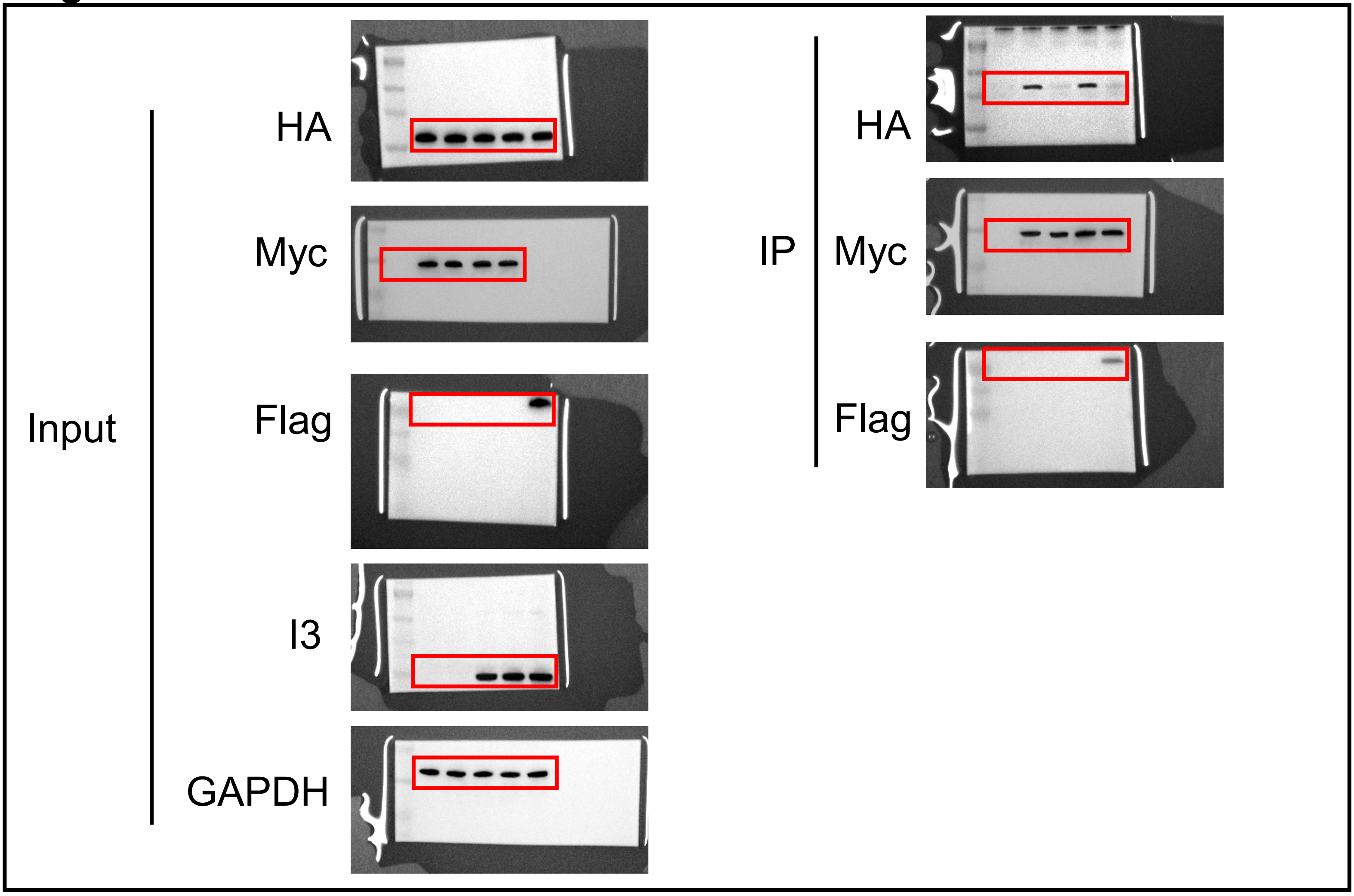

Figure 6E

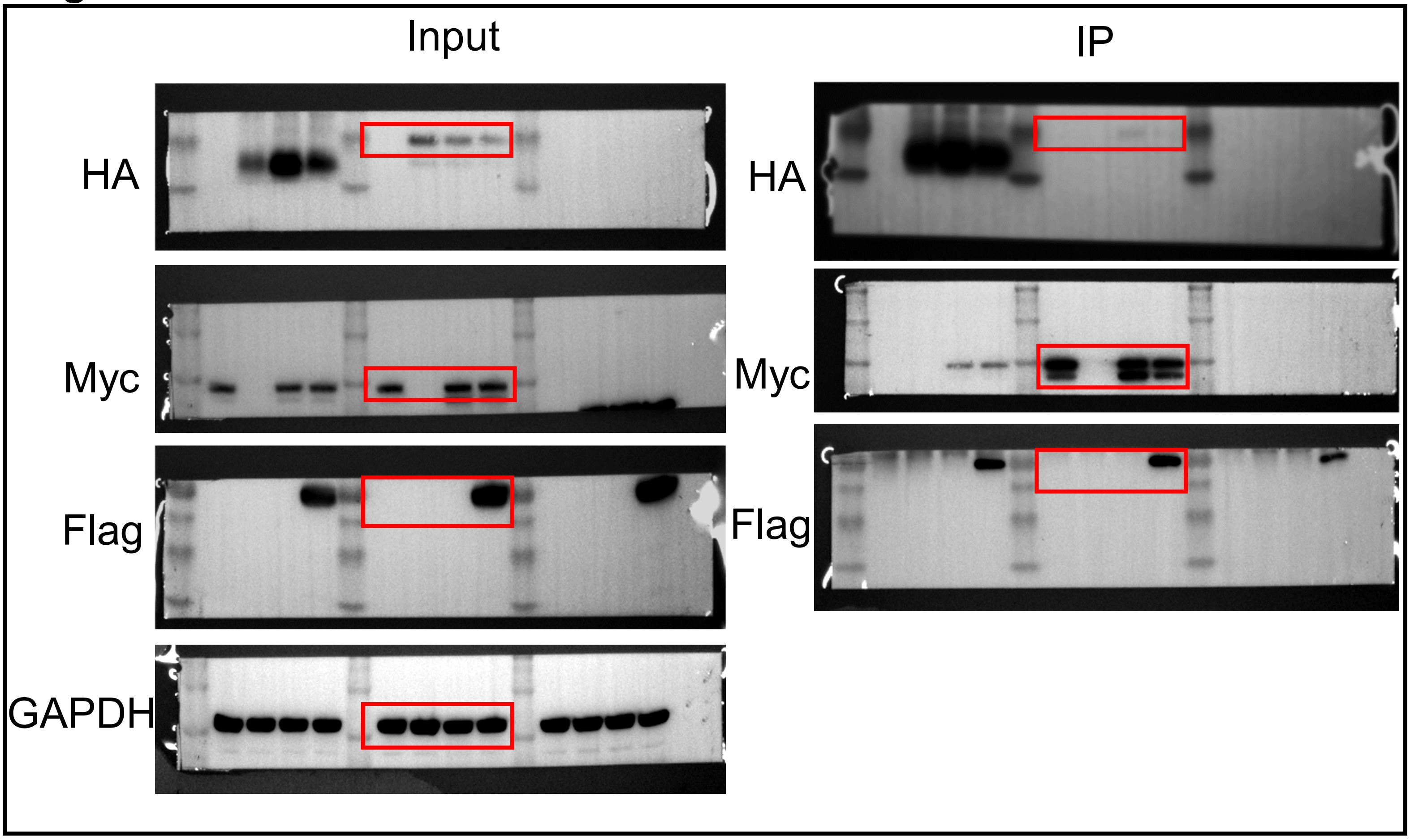

Figure 6F

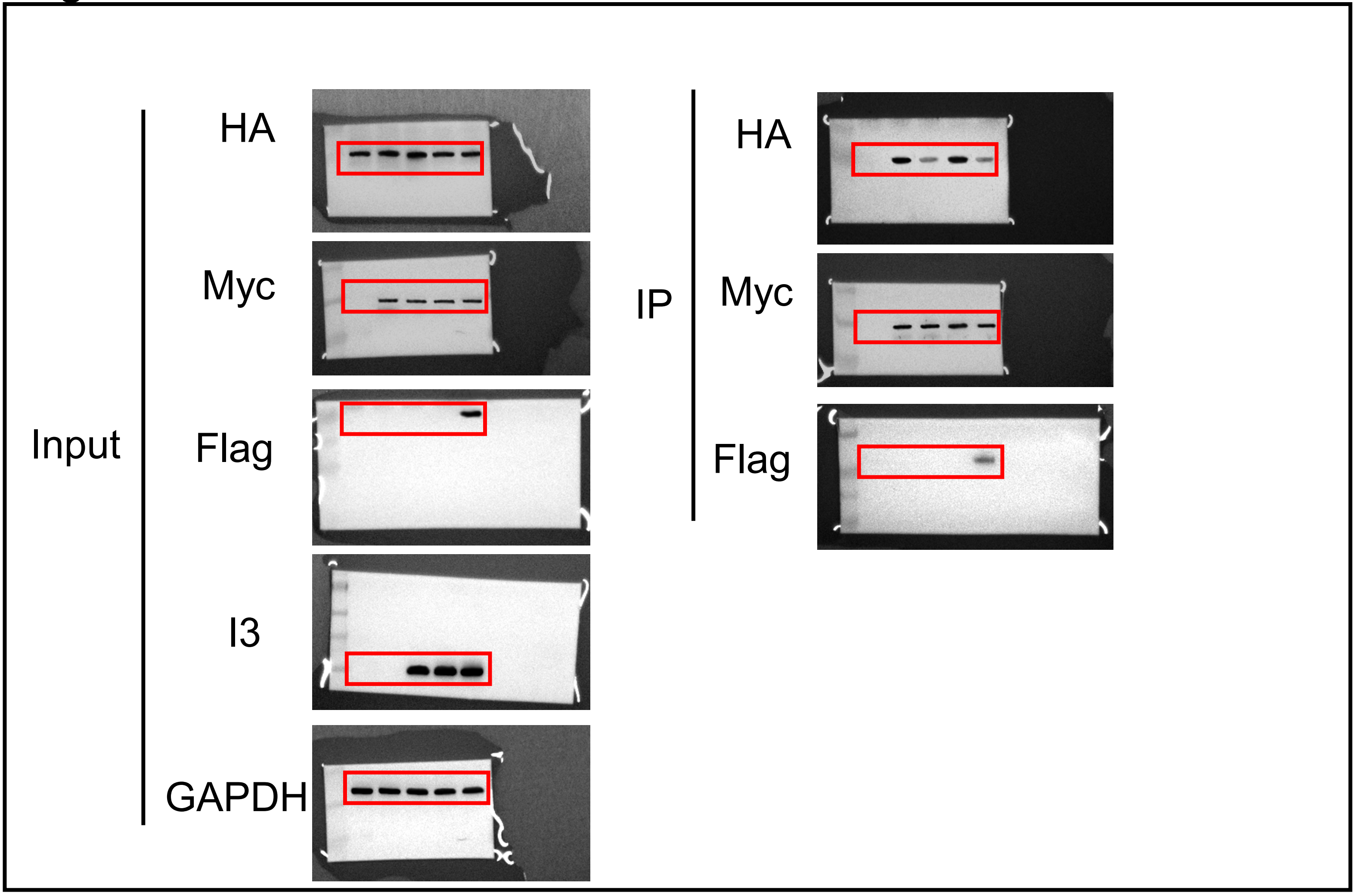

Figures 7A，7B and 7C

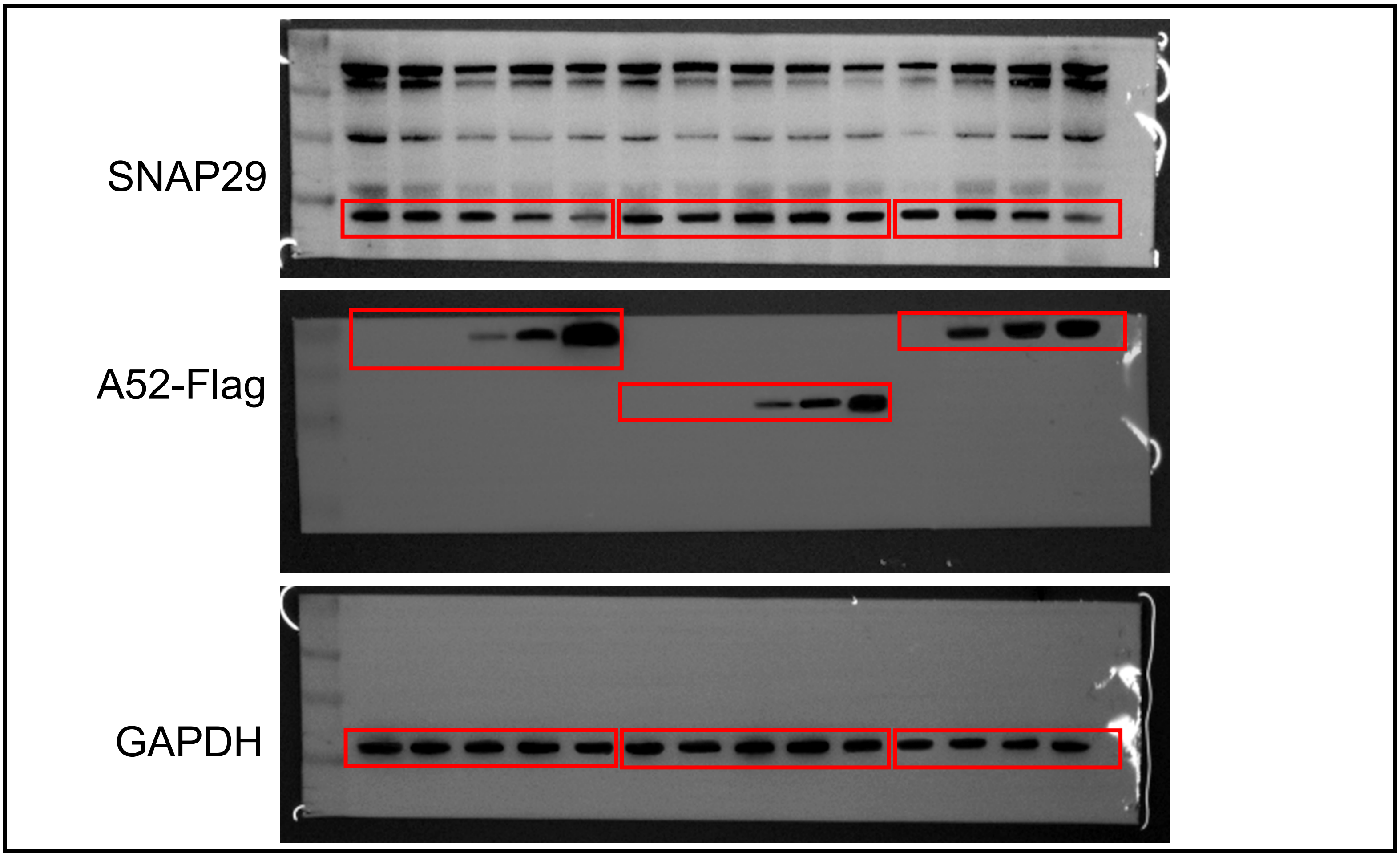

Figures 7D and 7F

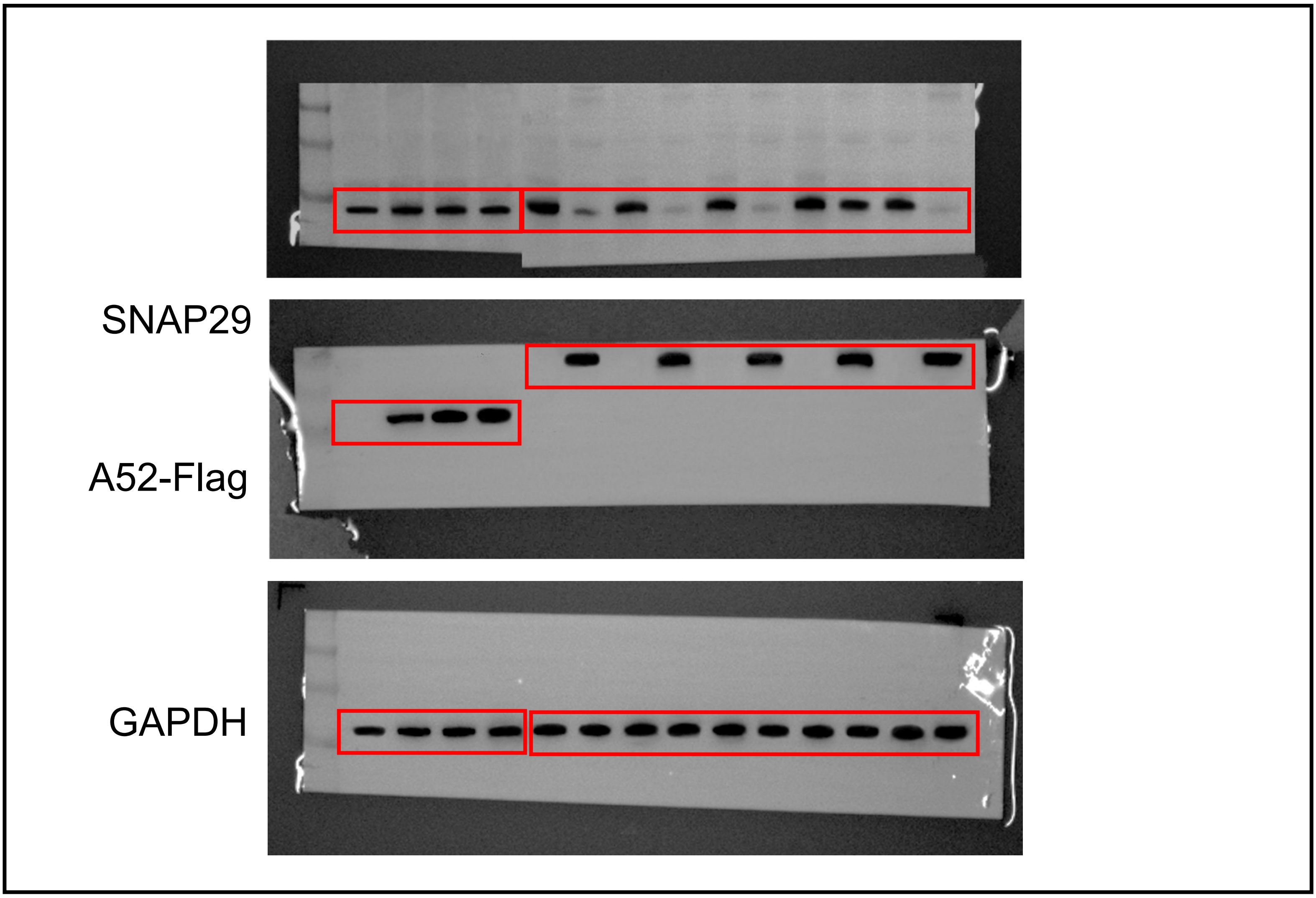

Figure 7E

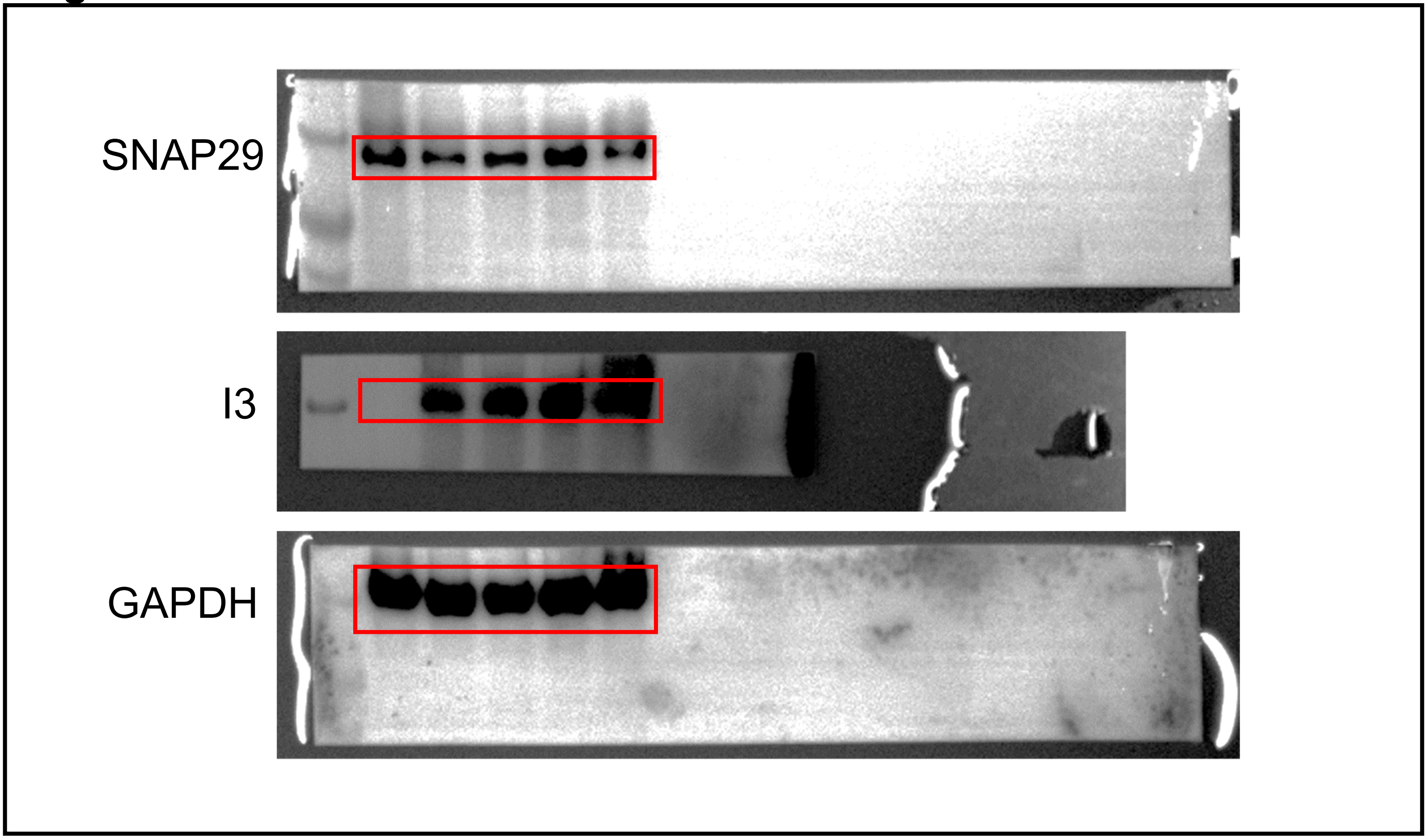

Figure 7G

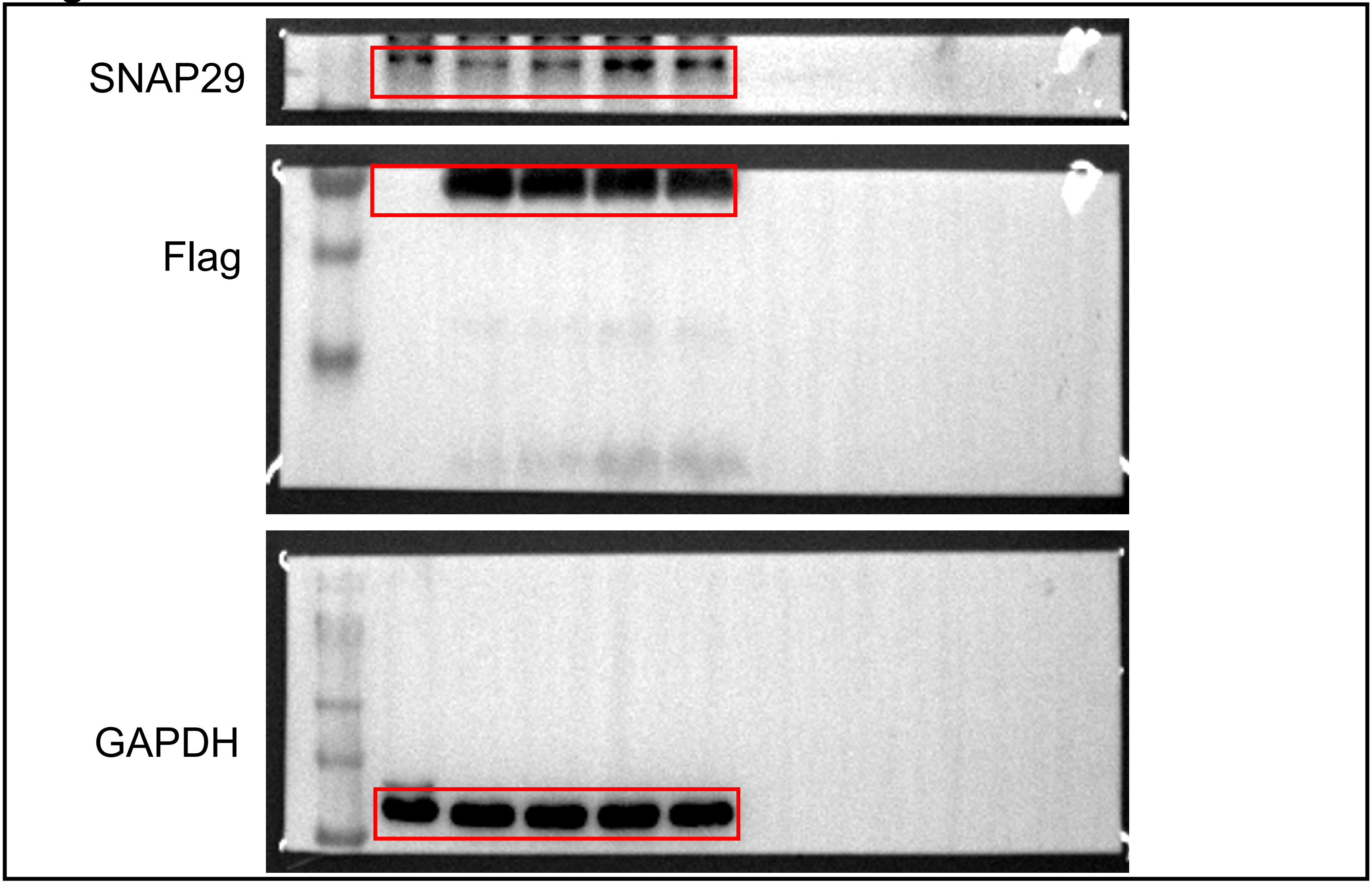

Figure 7H

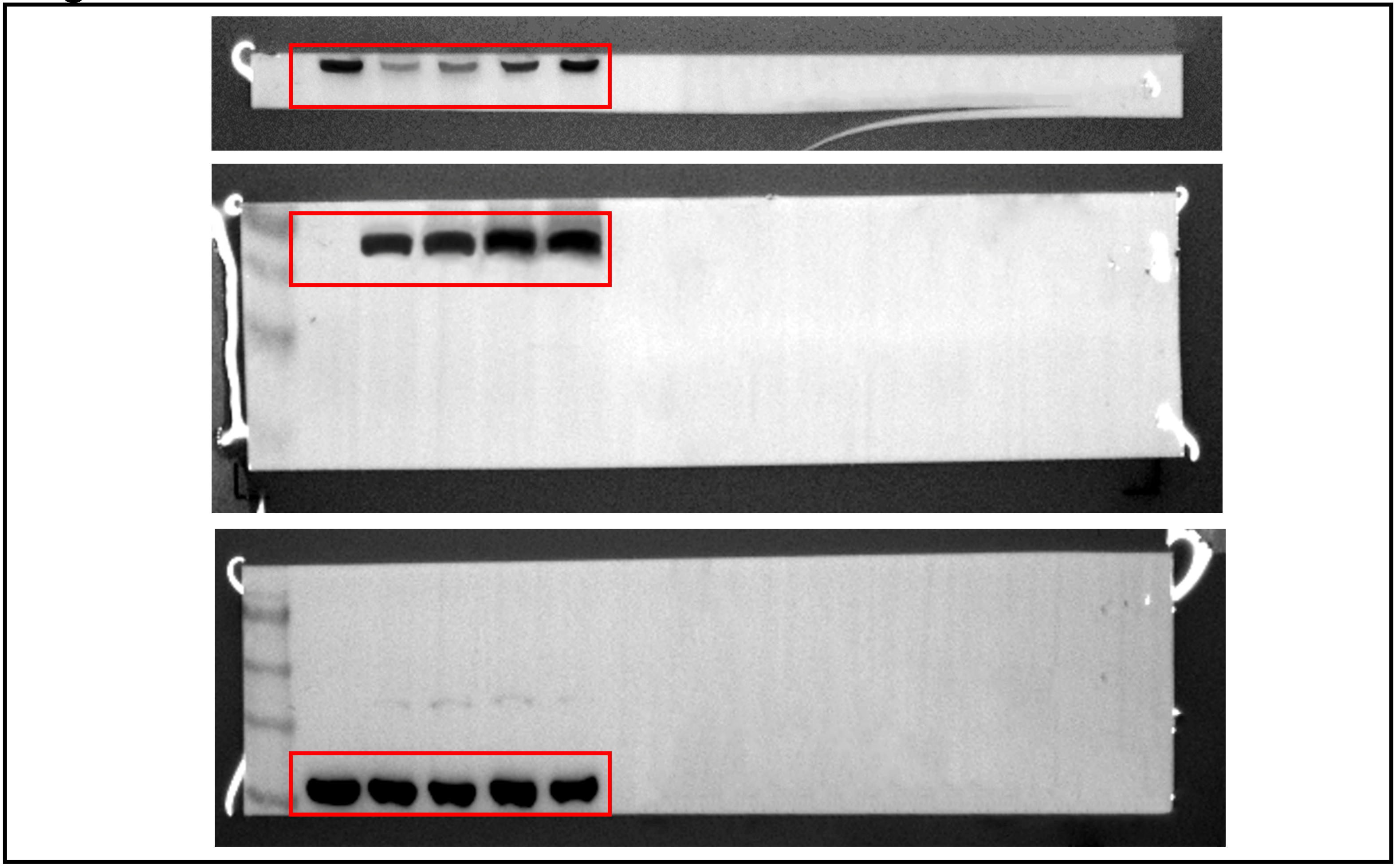

Figure 7I

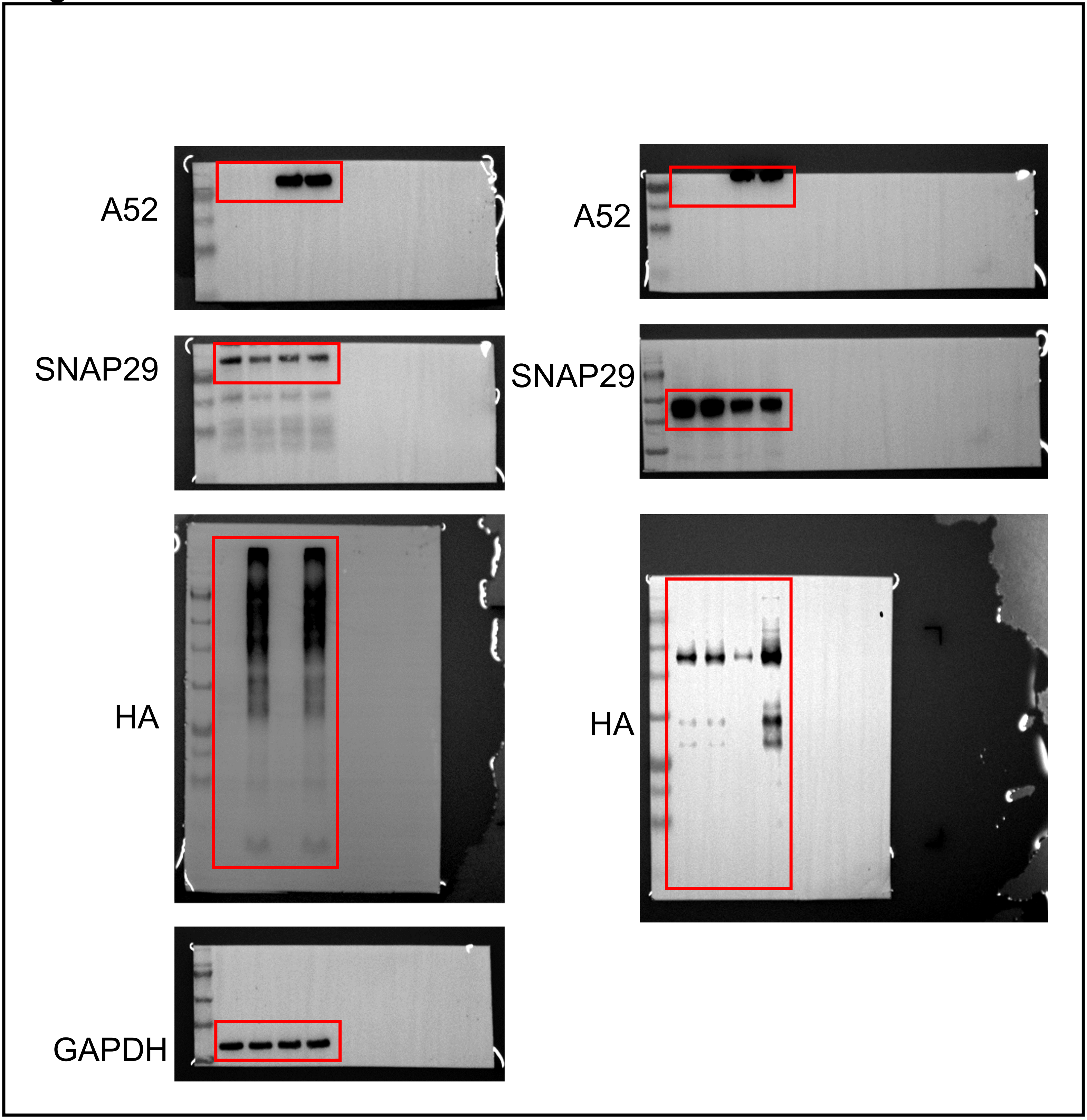

Figure 8A

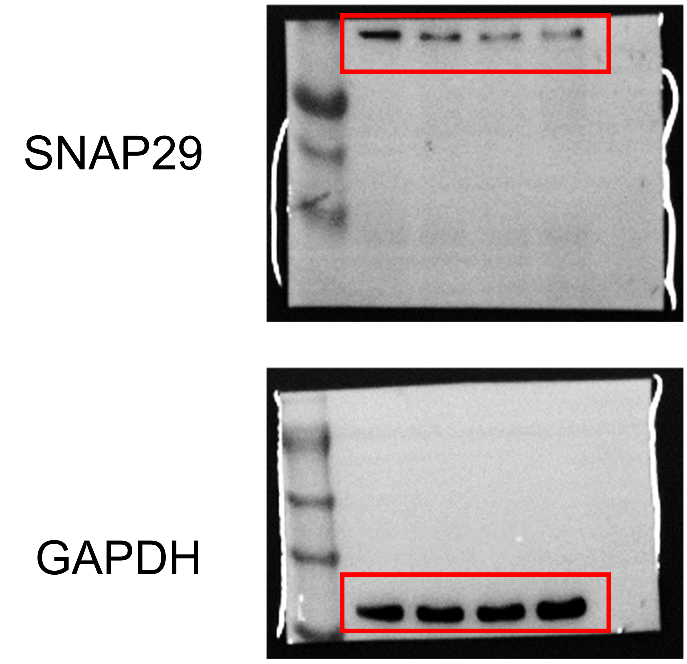

Figures 8C and 8G

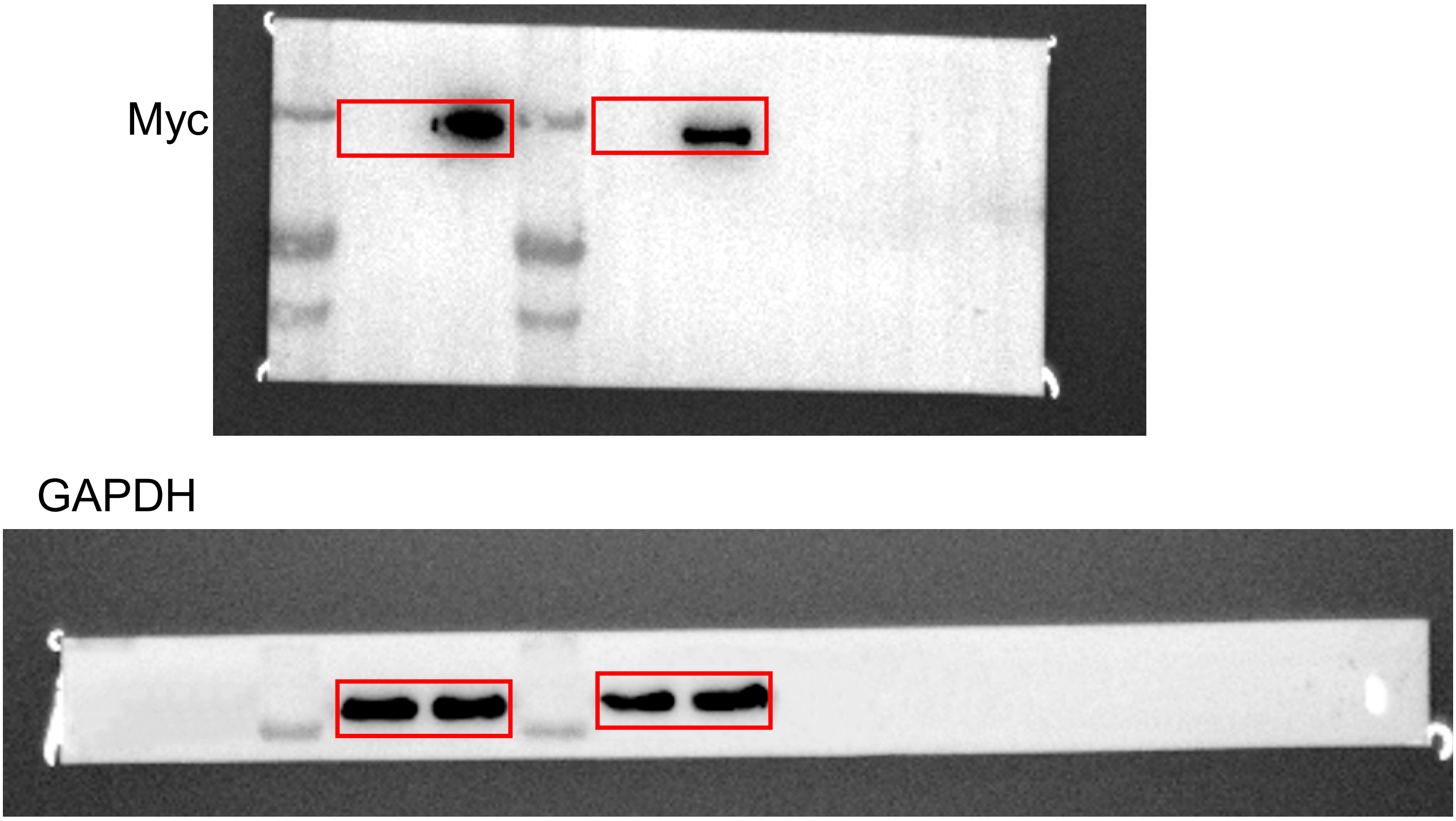

Figure 8J

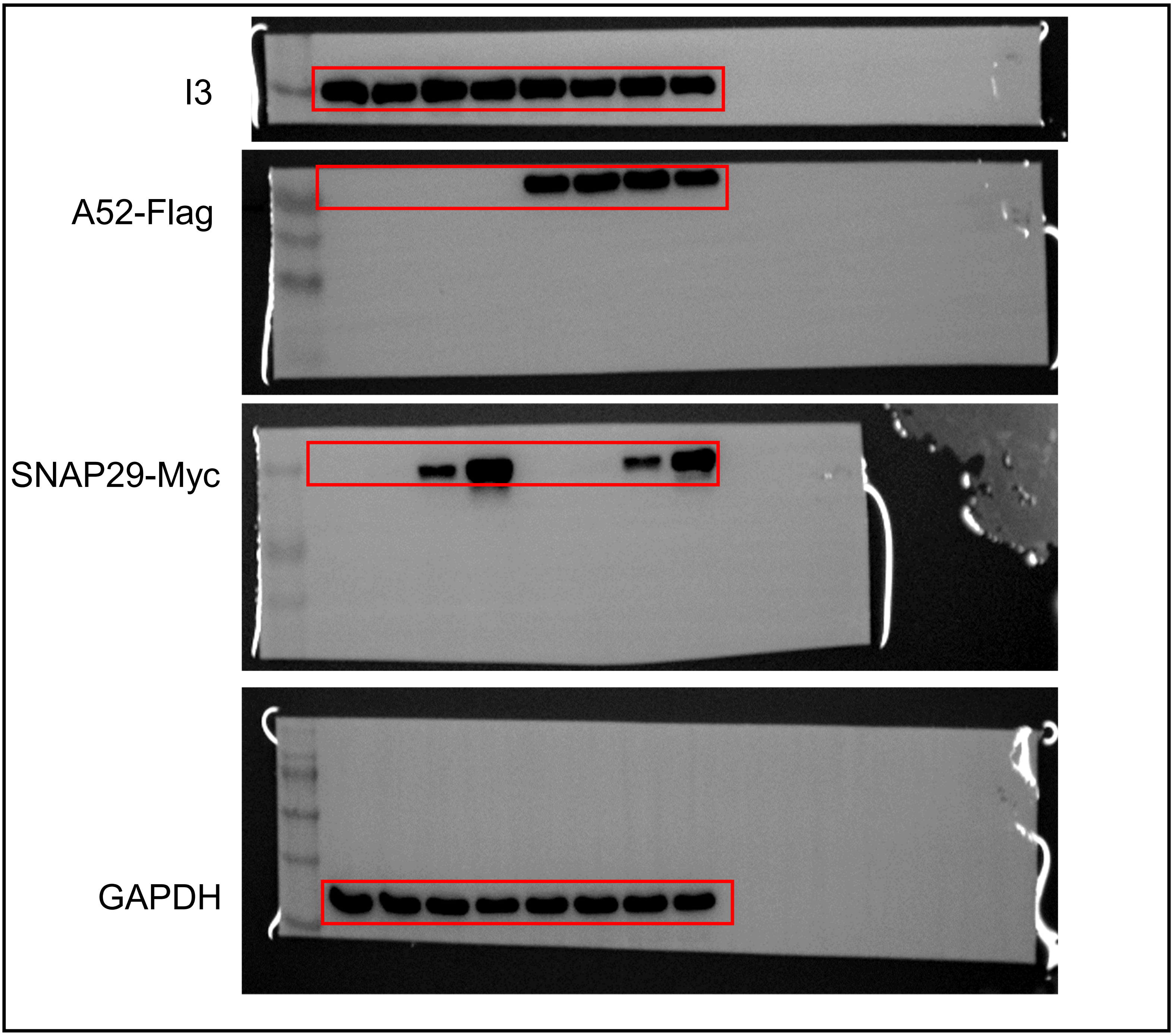

Figures S1J, S1K,S1L

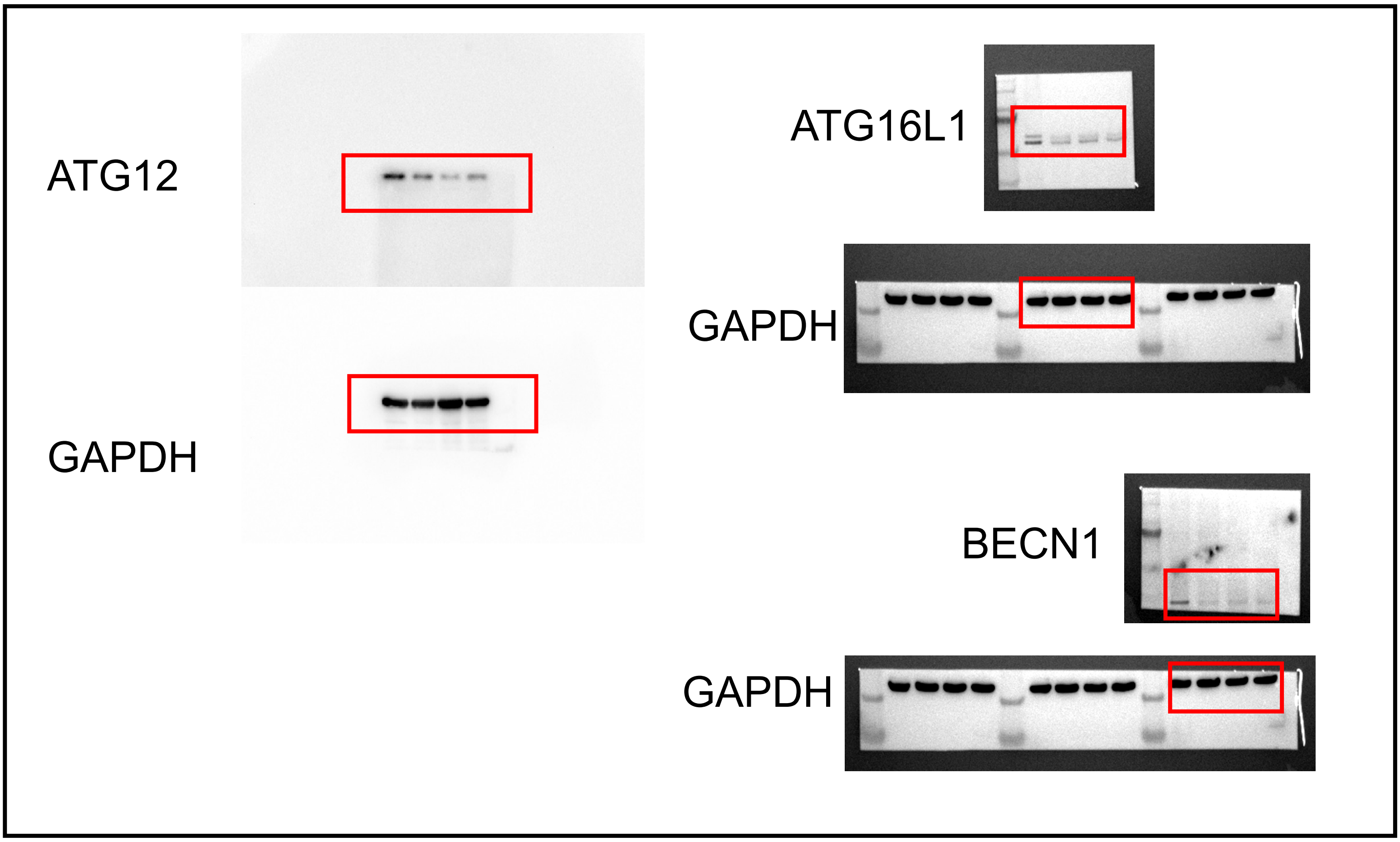

Figures S1M, S1N

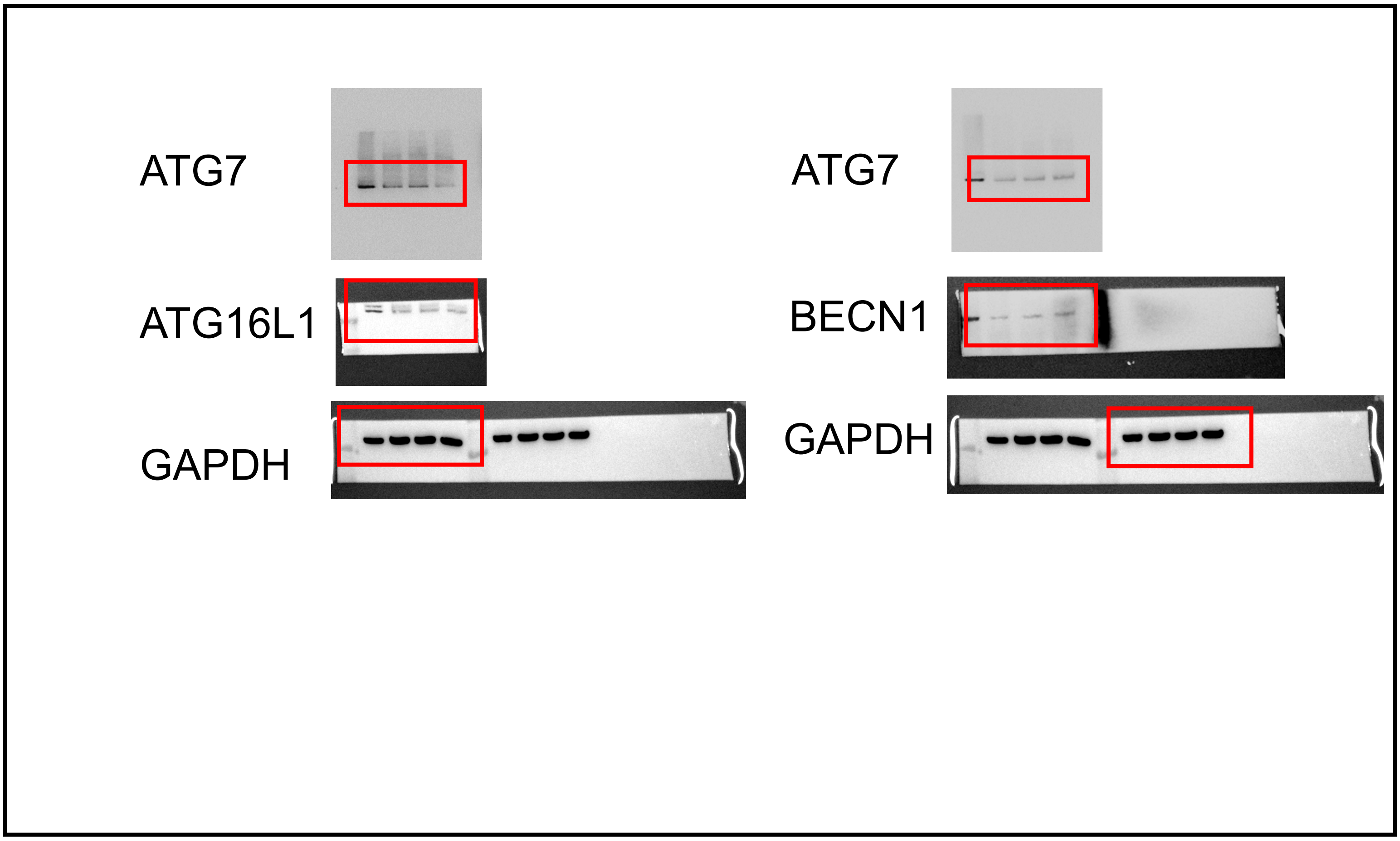

Figures S3B, S3C

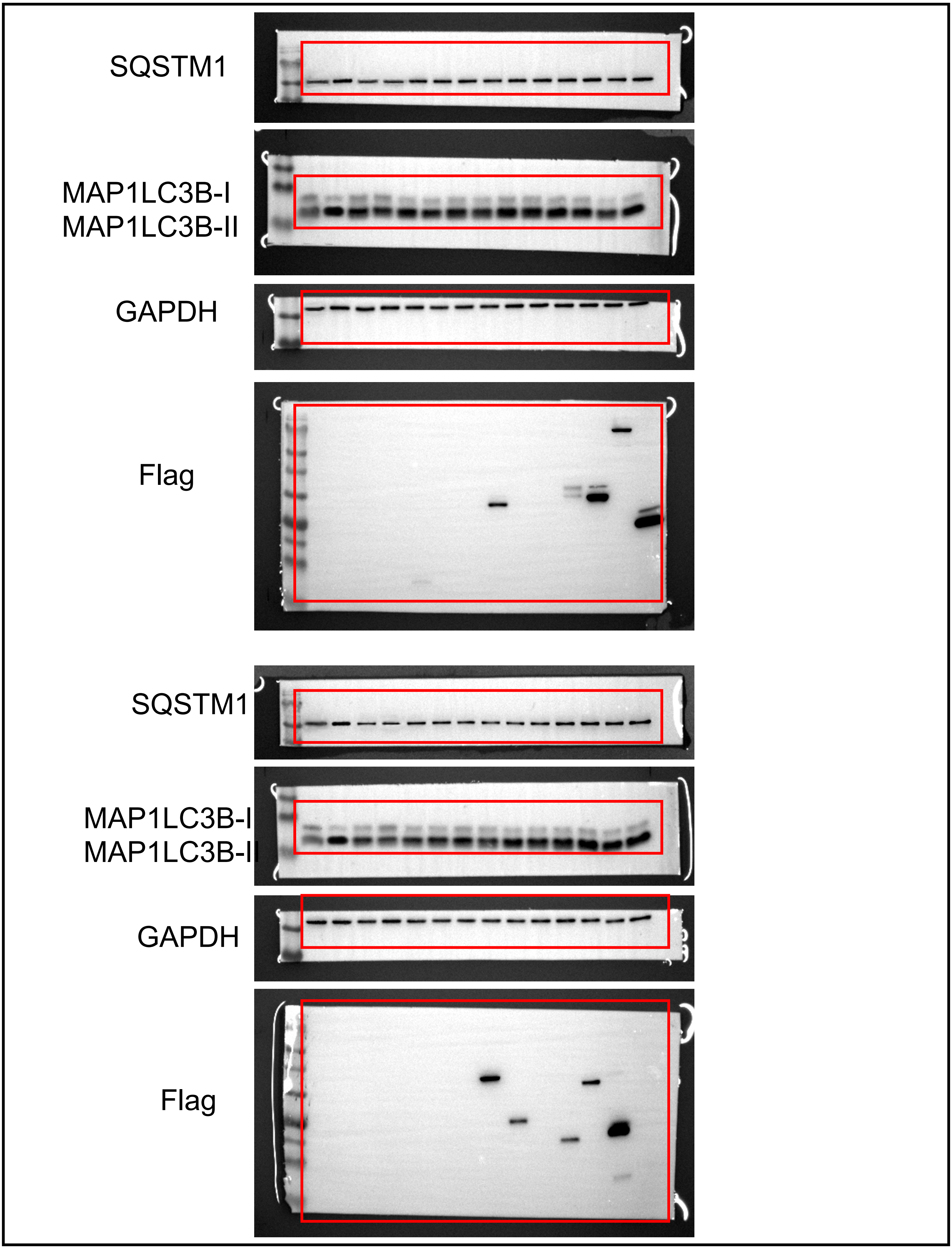

Figure S4B

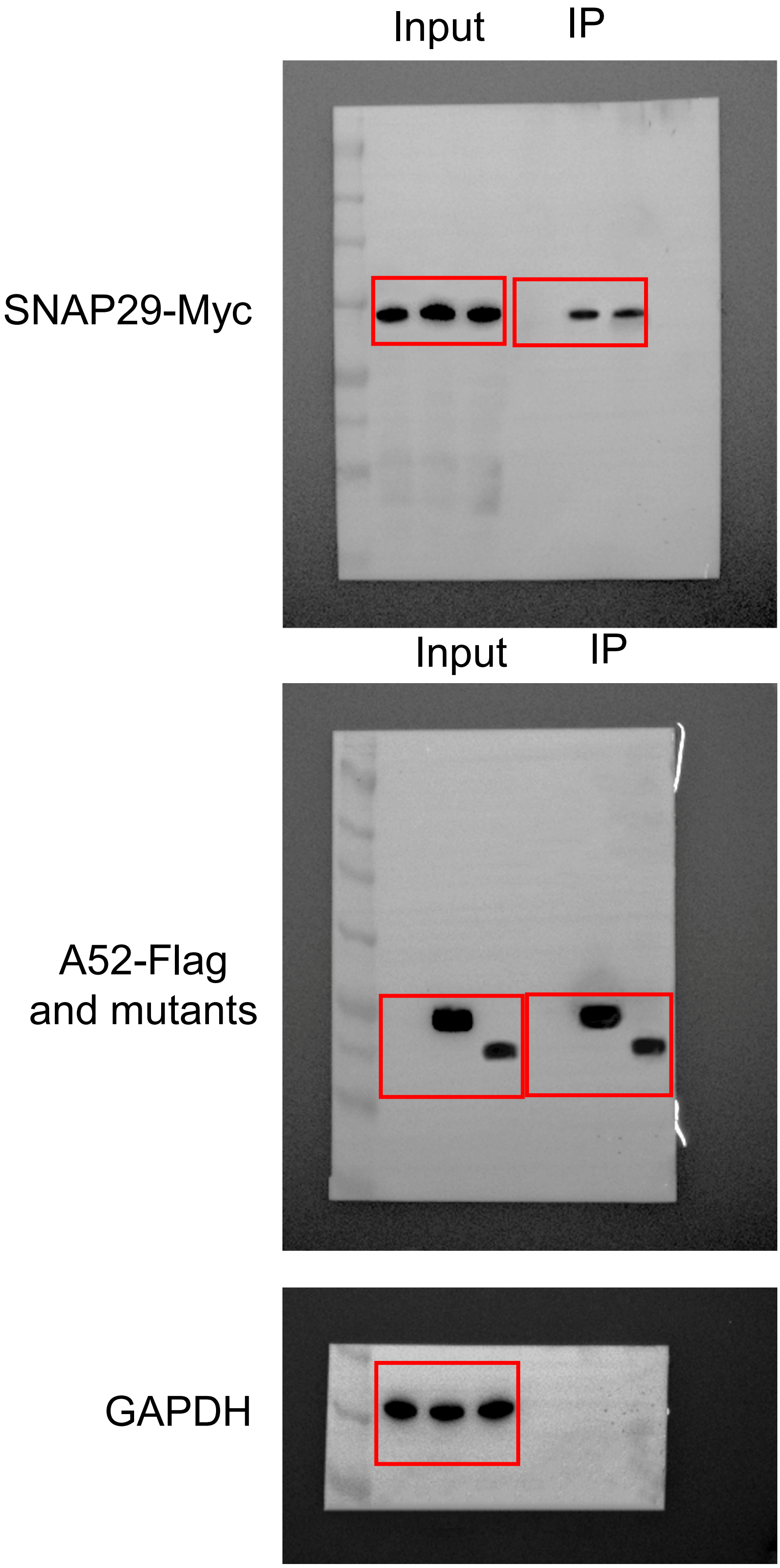

Figures S5F

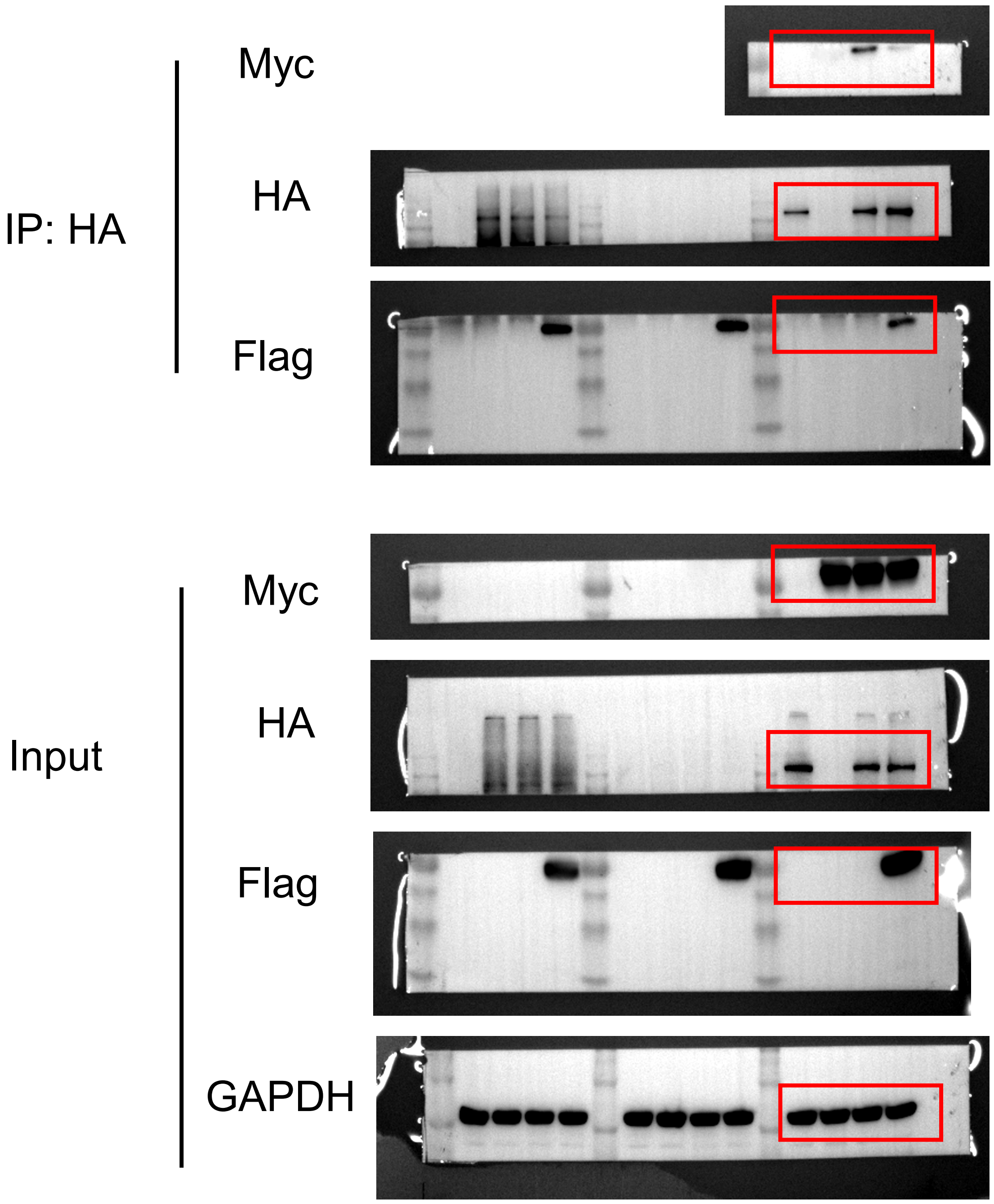

Figure 2G

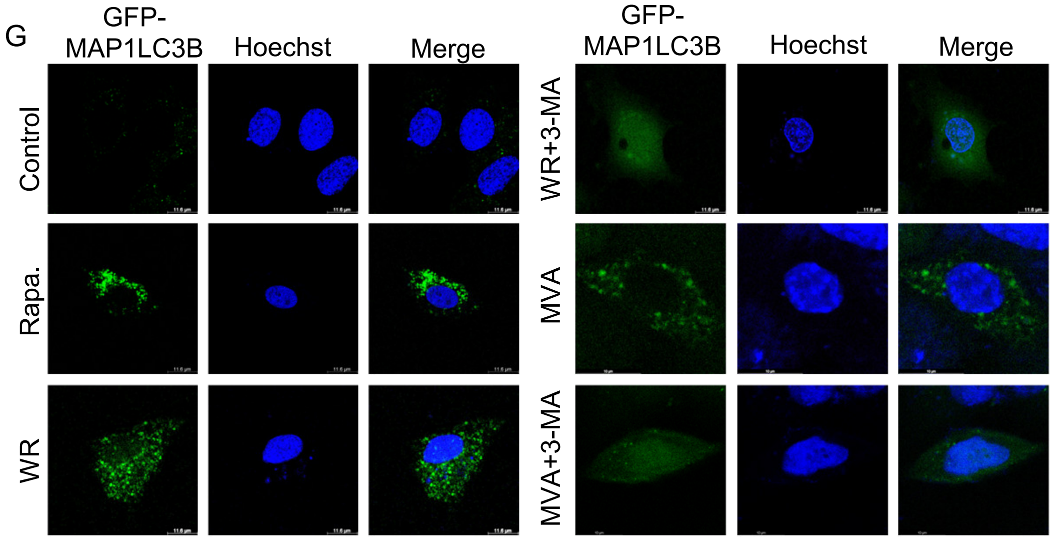

Figures 3B and 3D

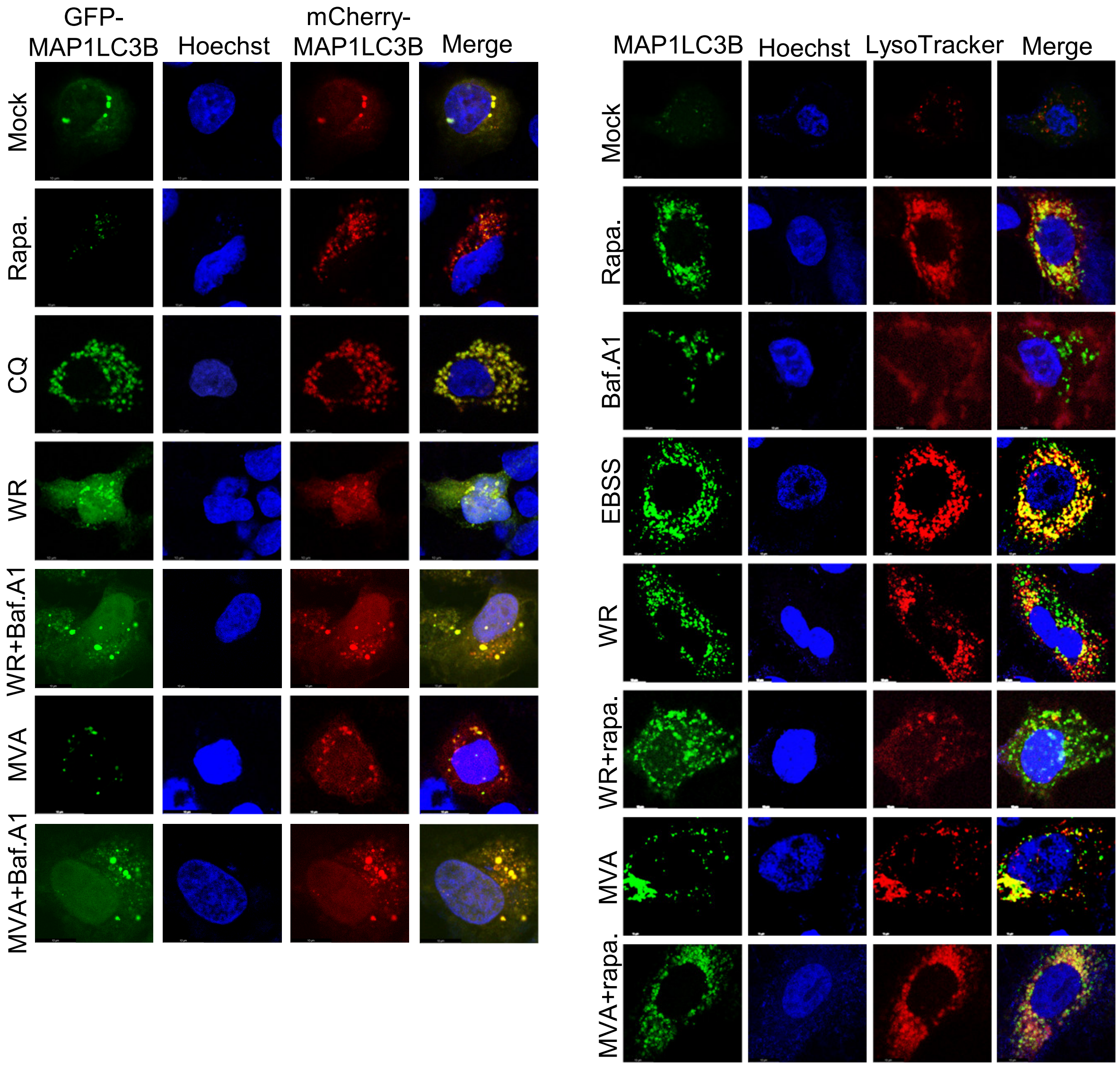

Figures 4A and 4G

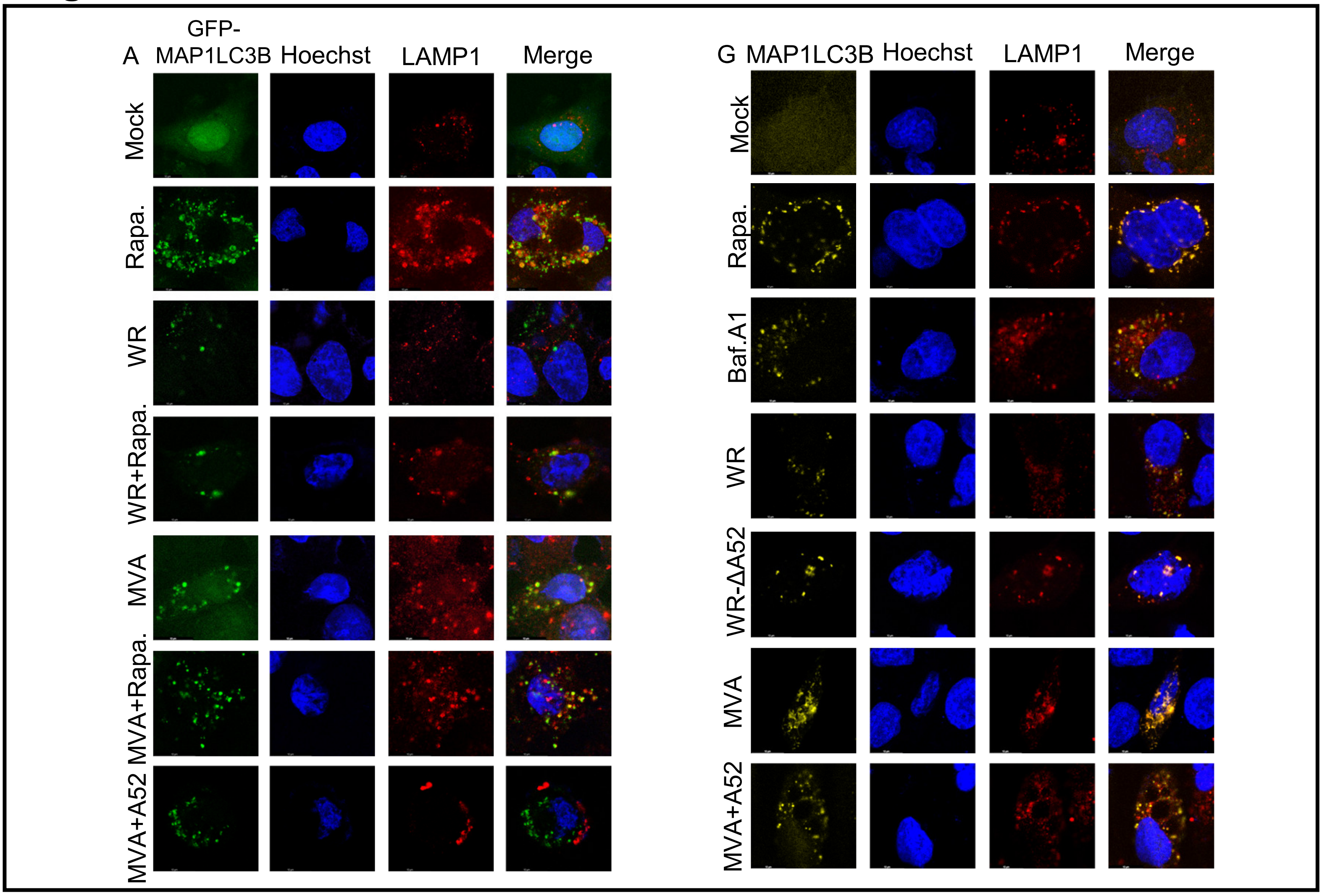

Figures 5D and 5G

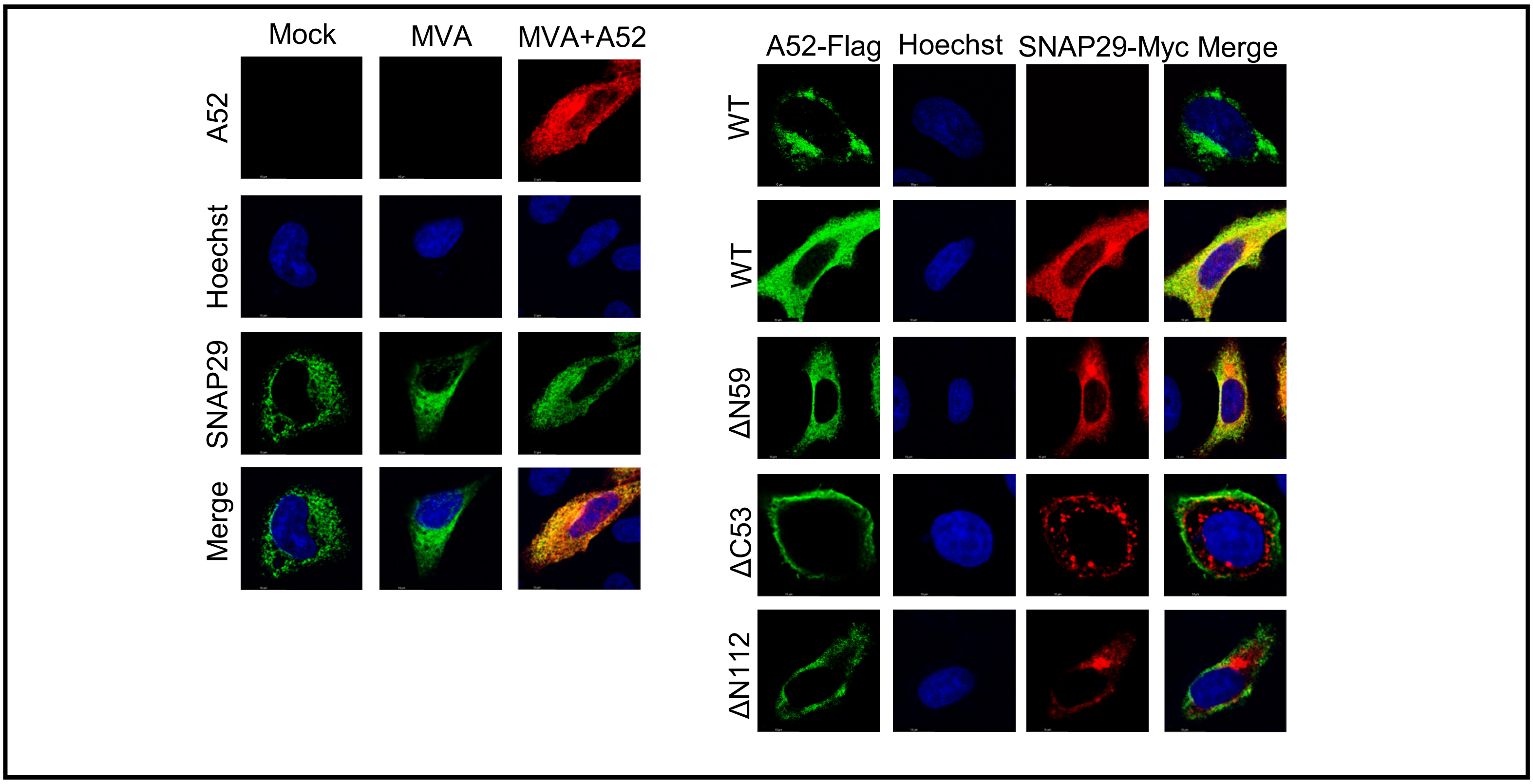

Figure 6C

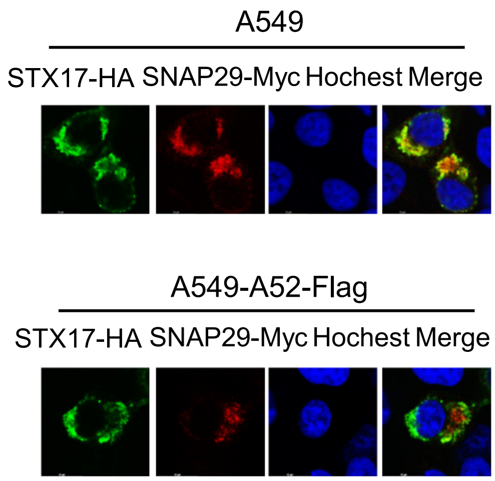

Figure 6H

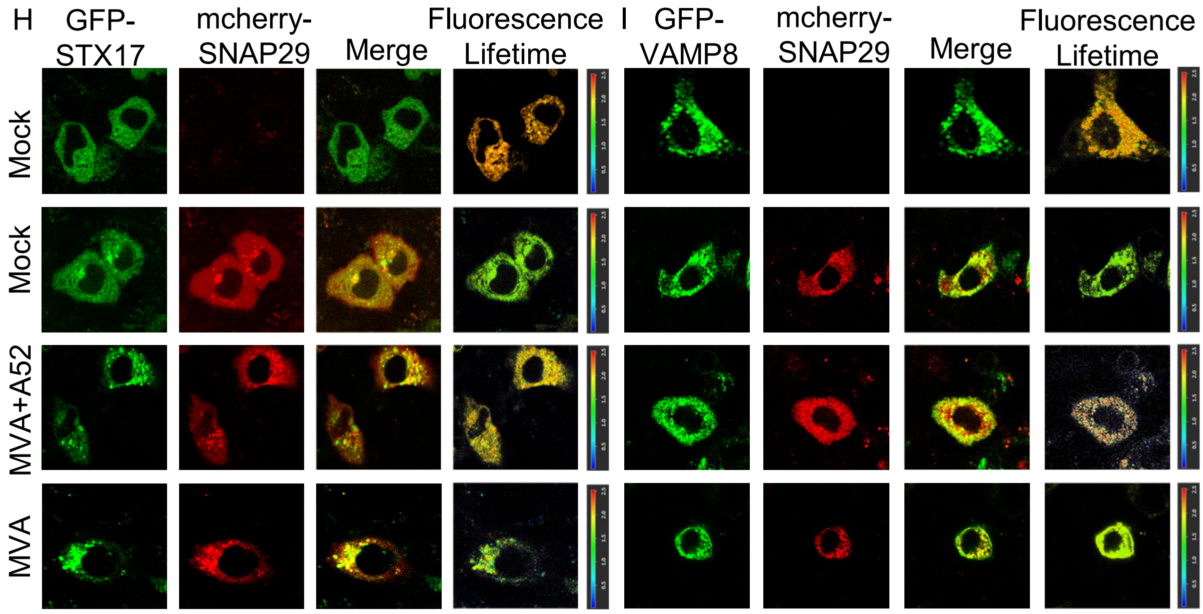

Figures S2A and S2C

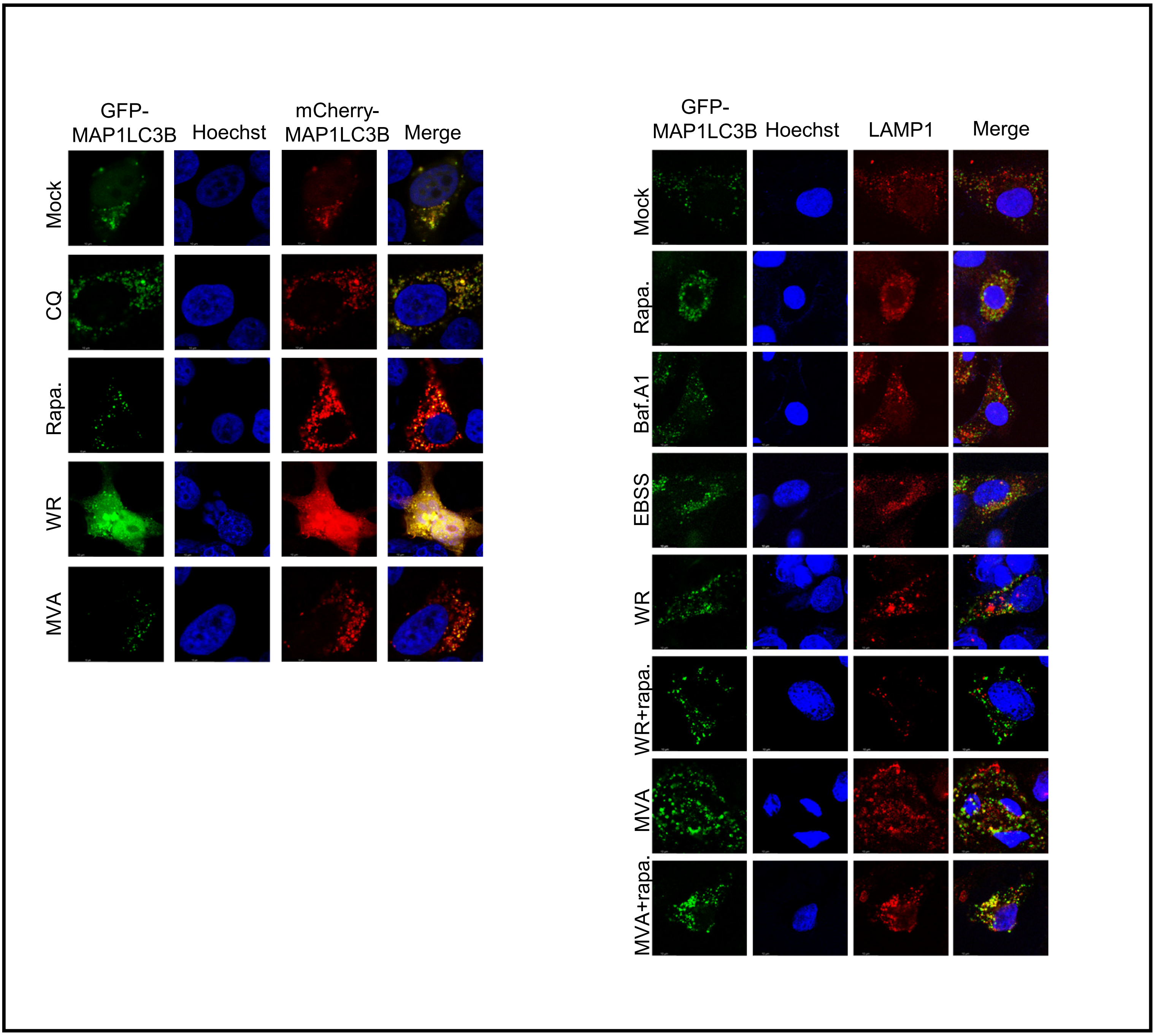

Figure S3A

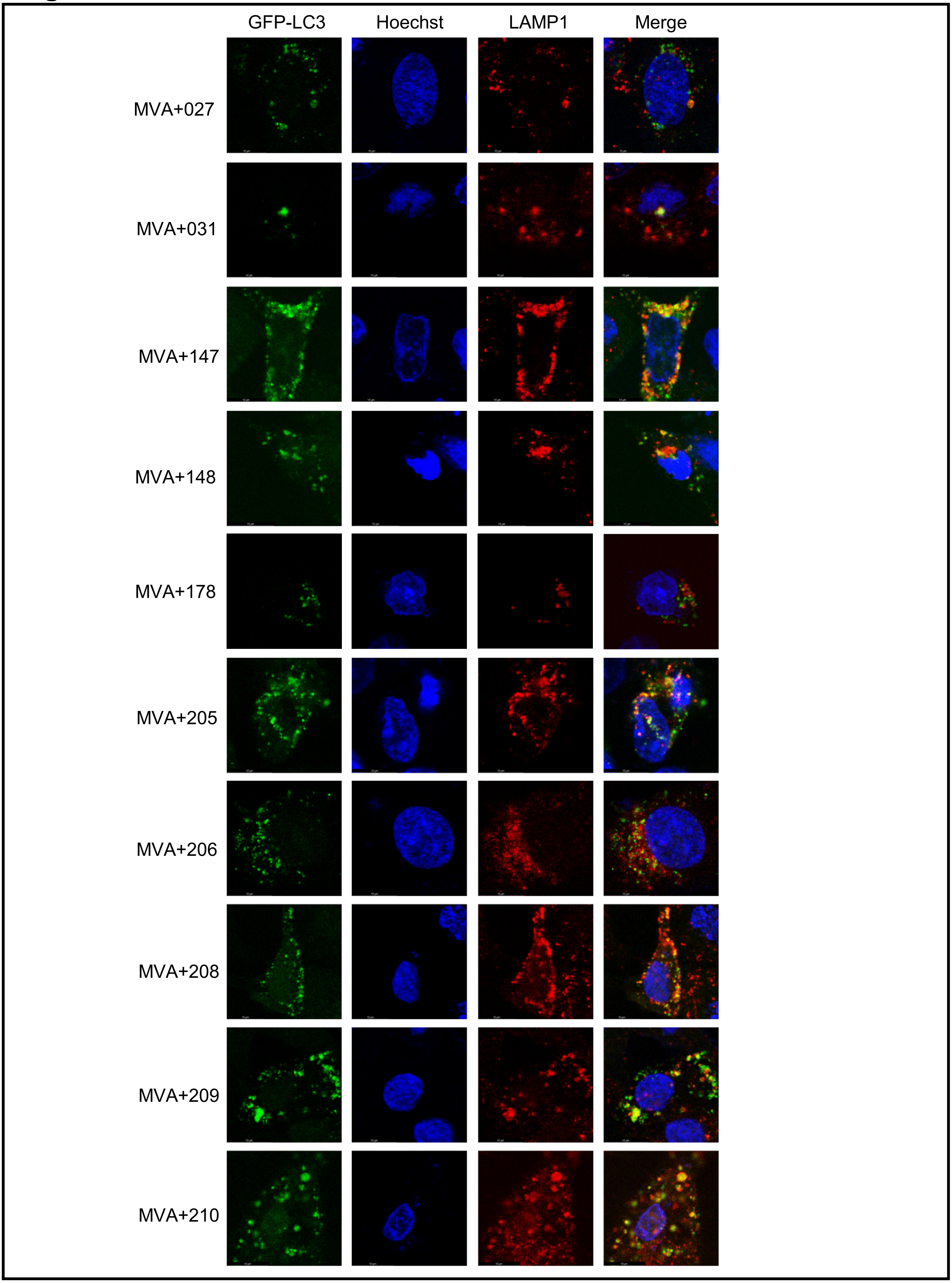
GFP-LC3
Hoechst
LAMP1
Merge
MVA+027
MVA+031
MVA+147
MVA+148
MVA+178
MVA+205
MVA+206
MVA+208
MVA+209
MVA+210

Figures S4A and S4C

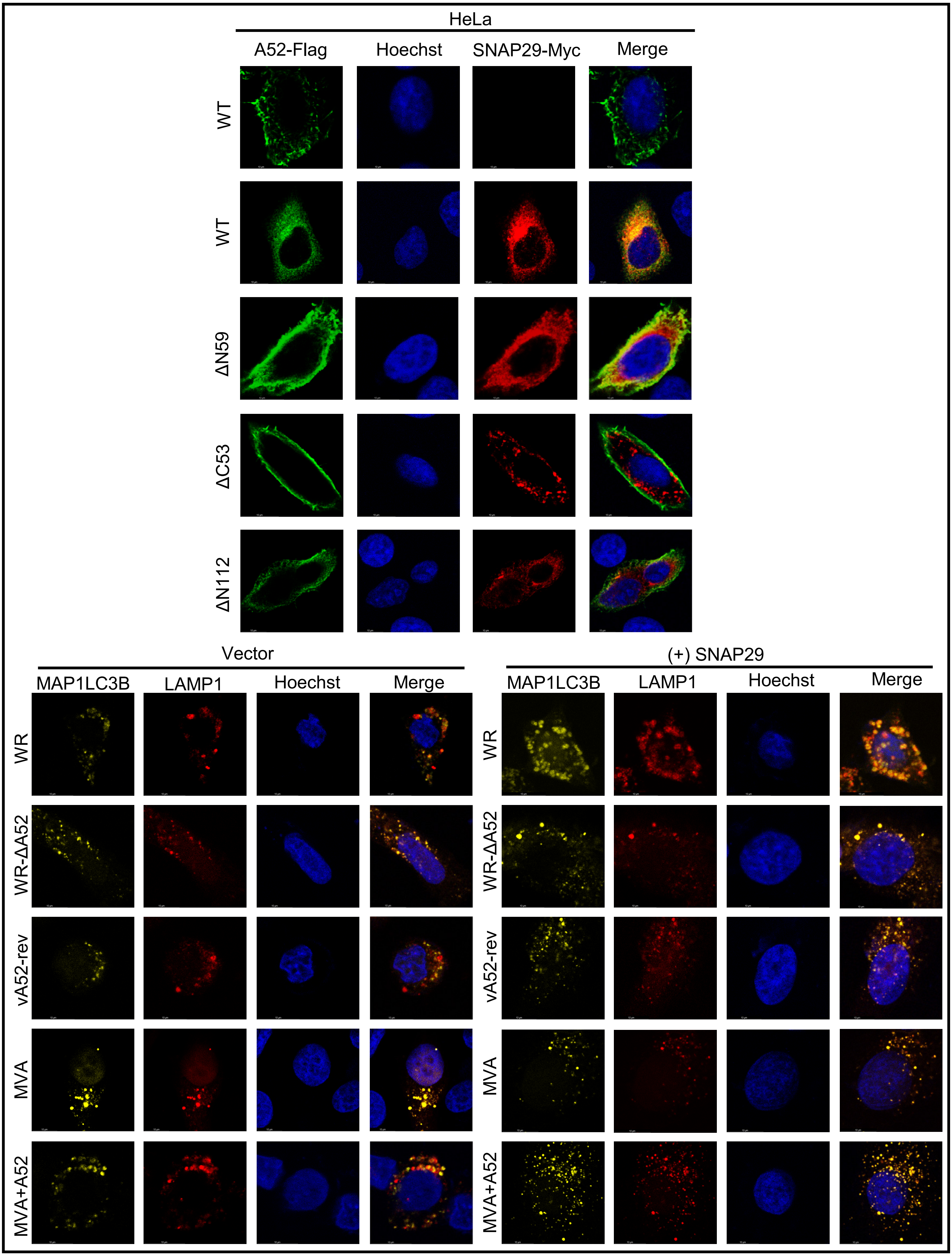

Figure S4D

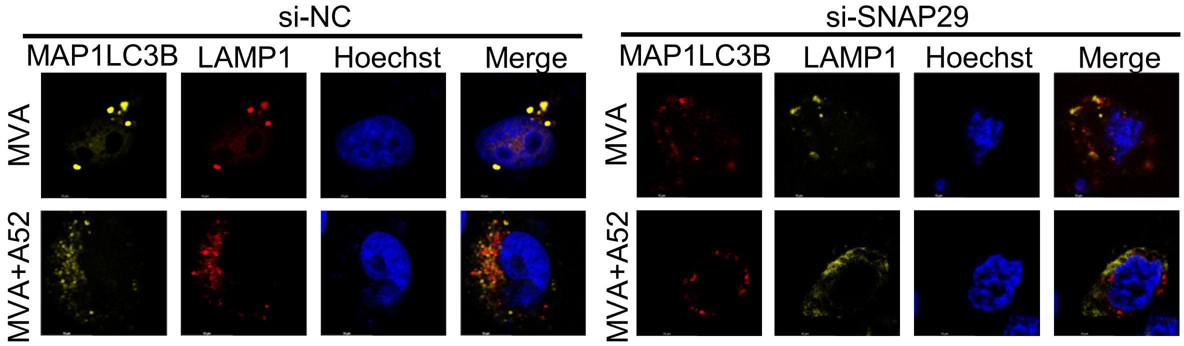

Figure S5H

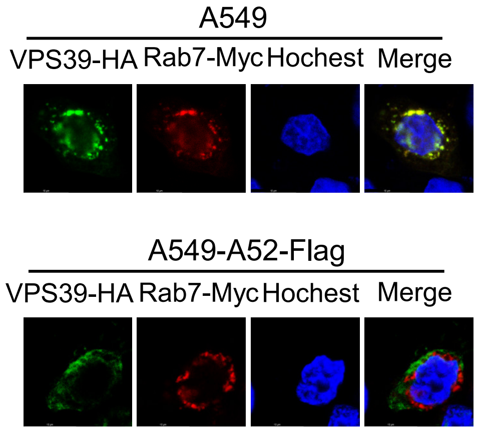

Supplement: S1 Data — (PDF) [file ppat.1014137.s008.pdf]
